# Partially unraveling mechanistic underpinning and weight loss effects of time-restricted eating across diverse adult populations: A systematic review and meta-analyses of prospective studies

**DOI:** 10.1371/journal.pone.0314685

**Published:** 2025-01-15

**Authors:** Duc Tran Quang, Nguyen Di Khanh, Linh Le Cu, Huyen Nguyen Thi Hoa, Chi Vu Thi Quynh, Quang Phan Ngoc, Thuy Bui Thi

**Affiliations:** 1 Faculty of Technology, Dong Nai Technology University, Bien Hoa City, Vietnam; 2 College of Health Sciences, VinUniveristy, Hanoi, Vietnam; 3 School of Medicine and Pharmacy, The University of Danang, Danang, Vietnam; 4 The Center Service For Technology Science Of Medi-Phar, Thai Binh University of Medicine and Pharmacy, Thai Binh, Vietnam; 5 National Institute of Nutrition, Hanoi, Vietnam; Federal University of ABC, BRAZIL

## Abstract

Time-restricted eating (TRE) is a promising and cost-effective dietary approach for weight management. This study aimed to evaluate the effects of TRE on weight loss in three adult populations using pre- and post-intervention analyses while also investigating its underlying mechanism. A systematic search was conducted across four databases (PubMed, Web of Science, Scopus, and the CENTRAL) up until January 28, 2024, specifically focusing on prospective studies that examined the efficacy of TRE in achieving weight loss. A random effects model was employed to conduct meta-analyses, while heterogeneity was assessed using the *I*^*2*^ statistic (PROSPERO: CRD42023439317). The study encompassed 36 selected studies involving 44 effect sizes and 914 participants. The effectiveness of the TRE was found to vary across health conditions, with modest weight loss observed in healthy individuals (pooled effect size -1.04 Kg, 95% CI: -1.42 to -0.65) and more significant weight reduction seen in participants with chronic diseases (pooled effect size -3.33 Kg, 95% CI: -5.05 to -1.62) and overweight/obesity (pooled effect size -4.21 Kg, 95% CI: -5.23 to -3.10). The observed decrease in body weight could be partially attributed to factors influencing energy balance, as evidenced by the significantly lower mean calorie intake at the end of the intervention (1694.71 kcal/day, 95% CI: 1498.57–1890.85) compared to the baseline intake (2000.64 kcal/day, 95% CI: 1830–2172.98), despite the absence of intentional efforts to restrict energy intake by the participants. These findings support the efficacy of this lifestyle intervention for weight loss maintenance and guide the development of its clinical guidelines.

## Introduction

In the face of a global obesity epidemic, the quest for effective and sustainable weight management strategies remains a paramount challenge in public health. Over the last four decades, the incidence of overweight and obesity has escalated dramatically worldwide, transcending economic boundaries and affecting populations across all income levels. The World Health Organization (WHO) estimated that in 2016, more than 1.9 billion adults were overweight, of which over 650 million were obese [1]. These figures are not merely statistical but represent a significant public health concern, given the association of overweight and obesity with increased risk of premature mortality, cardiovascular diseases, cancers, and type 2 diabetes [2].

Lifestyle-based interventions are recognized as foundational for effective weight management [3]. Recent studies emphasize the critical role of meal timing for metabolic health and well-being, while also highlighting the significant effects of manipulating the feeding-fasting cycle on various physiological and metabolic processes [4]. Among these, time-restricted feeding ([TRF] in animals) or time-restricted eating ([TRE] in humans), a salient approach within intermittent fasting (IF), has emerged as notably popular. TRE, restricting daily food consumption to a 4–10 hour window, is considered a feasible fasting regimen for most individuals due to its demonstrated clinical efficacy in promoting weight loss and ease of adherence by eliminating the need for calorie tracking during the eating window [5]. Alternatively, TRF has also been shown to increase insulin sensitivity, lower blood pressure, and to have other metabolic benefits in humans [6], with no serious documented side effects observed in previous studies of TRE [6, 7]. Despite the mounting evidence of the extensive health benefits of TRE for humans, the physiological and molecular mechanisms driving these effects remain incompletely understood [8]. To date, our understanding of TRF primarily stems from rodent studies, which suggest that the molecular mechanisms underlying the effects of altered meal patterns on metabolic health may be partly linked to synchronizing fasting-feeding times with the circadian rhythm [9].

Prior meta-analytic studies have explored the benefits of TRE across diverse adult demographics, yet the efficacy of TRE for weight management exhibits considerable variability, attributed to the lack of standardized TRE protocols [10–12]. This variability encompasses a broad spectrum of intervention durations (ranging from days to months) [10, 12, 13], fasting durations (12 to 20 hours) [10, 11, 13–15], and a predominant focus on fasting duration while neglecting dietary content in terms of type, quality, and quantity [4, 12]. Furthermore, research often targets singular demographic groups (such as healthy individuals, those overweight or obese, or those with chronic conditions) or exclusively employs specific methodological approaches (e.g., Randomized Controlled Trials) [10–12, 14, 15]. This heterogeneity obscures the comparative effectiveness of TRE across different adult demographic characteristics. Enhancing our understanding of TRE’s effectiveness for particular health profiles could significantly inform clinical practice and elucidate the physiological mechanisms underpinning weight loss.

Our review seeks to address some of the previously mentioned research gaps by comparing the impact of TRE on weight loss across three distinct adult demographic groups: those of healthy weight, the overweight/obese, and individuals with chronic diseases. This involves a systematic analysis of both pre- and post-intervention data derived from prospective studies methodologies. Therefore, our study aims to support clinical practice and improve our knowledge of the physiological mechanism involved in weight loss through the application of the TRE.

## Materials and methods

The study was designed, executed, and documented in accordance with the guidelines outlined by the Preferred Reporting Items for Systematic Reviews and Meta-Analyses (PRISMA) statement. Moreover, our protocol was prospectively registered with the International Prospective Register of Systematic Reviews (PROSPERO) under the registration number CRD42023439317.

### Search strategy

We integrated a multifaceted approach to identify relevant publications. First, a comprehensive literature search was performed across multiple electronic databases, including PubMed, Web of Science, Scopus, and Cochrane Central Register of Controlled Trials (CENTRAL). The search encompassed the entire available database content up to January 28, 2024, to identify relevant prospective studies focused on examining the impact of time-restricted eating on weight loss outcome among adult populations. Medical Subject Heading (MeSH) terms and relevant keywords were utilized for each database search, including terms such as *"intermittent fasting"*, *"time restricted eating"*, *“body weight”*. Only studies involving human participants and reported in the English language were included in the analysis. The ***S1 Table*** provides a depiction of the search strings and the corresponding searching strategy employed in this study. Second, a manual screening approach was employed to examine the reference lists of relevant original articles, review articles, and meta-analyses to identify any additional potentially eligible publications. Final, we sought consultation from subject matter experts to gather insights on pertinent studies related to the topic.

### Study selection

Three authors (PNQ, NDK, and VTQC) independently screened the titles and abstracts to evaluate the relevance of the articles according to the inclusion and exclusion criteria detailed in ***Table 1***. Subsequently, they thoroughly examined the full texts of the potentially eligible articles and made the final selection of studies. In cases where discrepancies arose during the literature screening process between the two authors, a third author (TQD) was involved to reach a consensus.

**Table 1:** Criteria for study selection.

	Inclusion	Exclusion
**Participant**	Adult individuals enrolled in the study, irrespective of their health conditions.	Studies conducted among children, adolescents, and lactating/pregnant populations.
**Intervention**	All types of TRE interventions delivered through various formats (e.g., 12:12, 14:10, 16:8) were considered eligible. However, prior to conducting the study, stringent criteria and well-defined procedures were established to ensure the clear implementation of the TRE intervention and to maintain intervention homogeneity within the intervention group. Furthermore, interventions combining TRE with other dietary or exercise approaches were also included, encompassing interventions aimed at reducing the consumption of unhealthy foods, enhancing nutrient intake, providing individualized dietary counseling, conducting group dietary classes, implementing standardized dietary prescriptions, and/or targeting calorie restriction for the purpose of inducing weight loss.	Other types of intermittent fasting include complete alternate day fasting, the 5:2 diet and religious fasting.
**Control**	No control groups or any control groups.	-
**Outcomes**	Firstly, the mean values of body weight (kg) changes from baseline, accompanied by their respective standard deviations (SDs) or other relevant statistical measures, such as standard error (SEs) or 95% confidence intervals (CIs), were computed within the intervention groups. Secondly, certain characteristics of TRE intervention. Lastly, the quantification of energy intake in kilocalories (calories) and macronutrient composition in percentage (%) was collected during the pre- and post-intervention period.	The percentage change in body weight from baseline was calculated within the intervention groups.
**Study design**	Nonrandomized or randomized controlled trials (RCTs) comparing pre- and post-intervention body weight in adult populations undergoing TRE interventions. No specific criterion was established for the duration of following the TRE.	Non-human studies, review, systematic review and meta-analyses, umbrella review, conference abstract, letter, case report, editorials and observational studies.
**Other**	No restrictions were imposed regarding publication date, genders or setting. In cases where multiple studies reported weight change estimates from the same cohort, we selected the study with the largest sample size or longest follow-up time for inclusion.	Non-english publications and duplicated populations.

### Data extraction

The data extraction process was carried out independently by two pairs of authors (LCL and PNQ, NDK and VTQC). A pre-established data extraction sheet was developed and pilot-tested using three studies. The extraction of data was further refined and standardized through multiple discussions among all authors. For each study, relevant information was extracted and recorded in a standardized Excel spreadsheet. This included the following details: (1) general study characteristics (first author, publication year, geographical location, trial registration, and funding details), (2) study population characteristics (sample size at the end of intervention, mean age, participant type), (3) TRE intervention characteristics (study design, type of TRE intervention, duration, overnight fasting, pre-post intervention data on calorie intakes and macronutrient compositions), and (4) intervention effects (mean body weight loss with corresponding 95% CIs between pre- and post-intervention, as well as any reported side effects). If an outcome was assessed at multiple follow-up time points, we included the effect intervention data separately for each time point. If 95% CIs were not provided, they were calculated based on the SEs or SDs of the mean. Studies with unavailable or insufficient data to calculate a 95% CIs were excluded from the meta-analyses and attempts to contact the authors for additional information were not made during the data collection process.

### Risk of bias assessment

Three independent reviewers (BTT, NDK and LCL) utilized the licensed Excel tool to apply the revised Cochrane risk-of-bias 2 tool for RCTs in identifying the risk of bias for relevant outcomes [16]. The assessment of bias encompassed five domains, namely, (1) randomization process; (2) deviations from intended interventions; (3) missing outcome data; (4) measurement of the outcome; and (5) selection of the reported result, with judgments assigned as high, low, or some concerns. Any discrepancies were resolved through discussion with a third investigator (TQD) to achieve a consensus.

### Statistical analysis

The subject group was divided into three categories based on the characteristics of participants undergoing TRE in the included studies: (1) Healthy adults (defined as individuals who are neither overweight/obese nor suffering from specific chronic diseases as per the selection criteria of the included studies), (2) Overweight/Obese (defined as individuals who are overweight/obese but do not have specific chronic diseases according to the selection criteria), and (3) Chronic diseases (defined as individuals with specific chronic diseases, with or without overweight/obesity, as per the selection criteria). Additionally, the included studies were stratified into two categories based on the mean weight loss: those with a weight reduction of less than 3 kg and those with a reduction of 3 kg or more. This threshold is clinically significant, as prior research has demonstrated that a 3–5% reduction in body weight is typically associated with clinical benefits [17]. Thus, this cut-off point serves as a critical benchmark for clinicians, researchers and participants to evaluate the minimal effectiveness of weight loss interventions. Time frame fasting, as delineated by the TRE implementation protocols in the methodologies of each included study, encompasses three primary types: unrestricted time frame fasting, overnight fasting, and daytime fasting. The unrestricted time frame fasting is defined as participants who are permitted to select their own eating and fasting periods based on their individual preferences and lifestyle. In contrast, some studies require all participants to adhere to a single, specified fasting period. Overnight fasting entails abstaining from food intake from evening until morning, generally aligning with an individual’s natural sleep cycle (e.g., fasting for approximately 20 hours, from 3:00 PM to 7:00 AM). In contrast, daytime fasting restricts food intake to specific hours within the day, with fasting periods occurring primarily during daylight hours (e.g., fasting for approximately 12 hours, from 6:00 AM to 6:00 PM).

Descriptive statistics, including frequencies and percentages, were calculated for participant demographics and intervention characteristics. The efficacy of TRE interventions in adults for body weight management was evaluated as a continuous variable, taking into account the pre-post intervention mean values, 95% CIs, and sample sizes reported in each individual trial. Data synthesis was conducted utilizing a random-effects model with inverse variance weighting, enabling the calculation of effect sizes with corresponding 95% CIs. Given the expected heterogeneity in TRE definitions, intervention protocols, and sample characteristics, the application of random-effects models was recommended [18]. Furthermore, this analytical approach is regarded as more robust for generating reliable estimates of treatment effects in clinical trials [19]. The presence of heterogeneity among the studies was evaluated using Cochran’s Q statistic and the *I*^*2*^ test. A subgroup analysis was performed to investigate the essential features of the diet intervention and clearly delineate its effectiveness across different geographical regions, age groups, characteristics of TRE intervention, research designs as well as changes in calorie intake and macronutrient composition among the three distinct categories of adult participants. A sensitivity analysis was conducted to evaluate the potential impact of individual studies on the overall findings by employing the "leave-one-out" approach, thereby assessing whether any specific study exerted disproportionate influence on the results. Specifically, if the exclusion of a particular study (k) resulted in a substantial alteration in the statistical significance of the effect, transitioning from P < 0.05 to P > 0.05 (or P > 0.05 to P < 0.05), we classified it as an influential case [20]. Using univariate meta-regression analysis, we assessed the effects of trial duration, participant age, changes in calorie intake, and mean differences in weight loss. To assess publication bias, a funnel plot was constructed and visually inspected for asymmetry. Additionally, publication bias was evaluated using Egger’s regression and Begg’s rank correlation tests. Quantitative two-group data were subjected to analysis utilizing an unpaired two-tailed t-test, wherein statistical significance was determined at a P value less than 0.05. The meta-analysis was using univariate meta-regression analysis, we assessed the effects of trial duration, participant age, changes in calorie intake, and mean differences in weight loss. In instances of missing data or to validate the reliability of data within the included meta-analyses, we opted to extract and compute the missing data or directly verify questionable data from the individual primary studies [18]. The meta-analyses were conducted using the STATA software (Version 14, Stata Corporation, College Station, TX, USA).

### Patient and public involvement

The development of research questions, study design, and outcome measures did not involve any patients or members of the public. Patient input was not sought for result interpretation or writing.

## Results

### Literature search and study characteristics

The initial database search yielded 2,574 articles, which were then screened based on predefined exclusion criteria. After this screening process, 119 articles remained for full-text evaluation for eligibility. Among these, 36 studies with 44 effect sizes were included in the subsequent meta-analyses [21–56].

**Fig 1:**
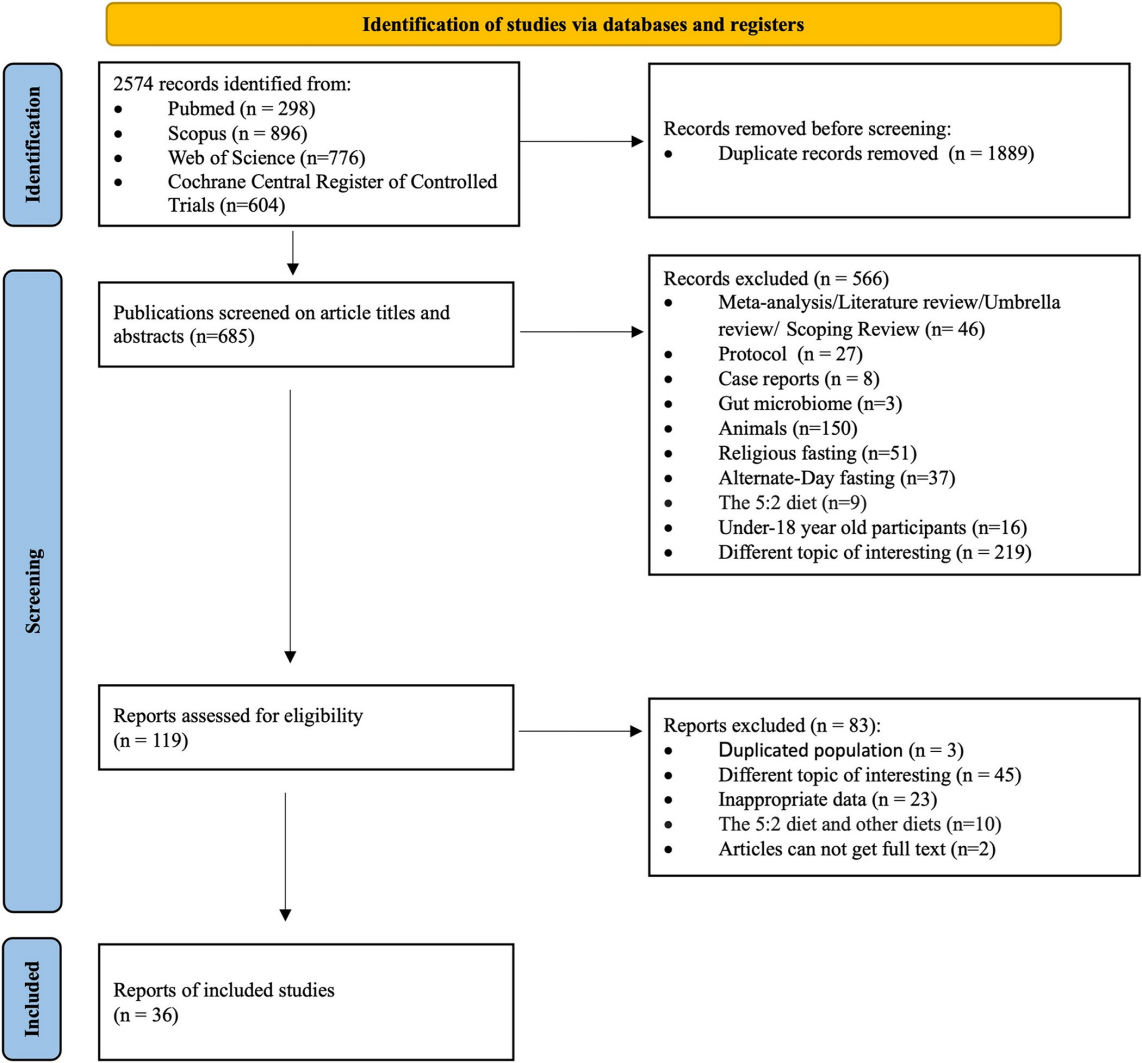
Flowchart depicting the process of study search and selection.

***Fig 1*** visually presents the study selection procedure. ***Tables 2*** and ***S2*** provide a concise summation of the principal characteristics exclusively observed within the intervention arm of the included studies. The studies encompassed a diverse range of countries and territories, with a predominant focus on non-Asian regions, specifically Australia (1), Brazil (2), Canada (2), China (4), Germany (2), Iran (1), Italy (3), Kenya (1), South Korea (2), the Netherlands (1), Switzerland (1), Taiwan (1), Turkey (1), the United Kingdom (1), and the United States [13]. Within the selected studies, the sample sizes varied, ranging from 32 to 626 participants. The age distribution encompassed a broad spectrum, spanning from 19 to 77 years old, with a mean age of 41.61 ± 6.66 years. At baseline, the total participant cohort consisted of 914 individuals, among whom 241 subjects (mean age 50.03 ± 8.9 years) exhibited chronic diseases, 151 subjects (mean age 26.69 ± 3.75 years) comprised healthy adults, and 522 subjects (mean age 44.42 ± 7.0 years) were characterized by overweight or obesity. The age distribution within the two groups, classified based on weight loss of less than 3 kg and weight loss of 3 kg or more, demonstrates a comparable distribution of ages, with a prominent concentration observed within the age range of 30–50 years. Concerning the salient features of TRE regimen within these two groups, there was a similarity in the duration of daily fasting, with both groups observing an average of 15.9 ± 1.50 and 15.6 ± 2.01 hours, respectively. Notably, these fasting periods primarily transpired overnight, spanning a temporal range encompassing 9.79 ± 9.43 weeks to 15.7 ± 12.0 weeks.

**Table 2:** Summary of participant characteristics in studies included in meta-analyses.

Characteristics	Healthy adults		Overweight/Obesity		Chronic diseases		Overall	
	< -3 kg (N = 10)	> = -3 kg (N = 0)	> = -3 kg (N = 7)	> = -3 kg (N = 16)	< -3 kg (N = 7)	> = -3 kg (N = 4)	< -3 kg (N = 24)	> = -3 kg (N = 20)
**Locations**								
Asia	4 (40.0%)	-	0 (0%)	2 (12.5%)	2 (28.6%)	3 (75.0%)	6 (25.0%)	5 (25.0%)
Non—Asia	6 (60.0%)	-	7 (100%)	14 (87.5%)	5 (71.4%)	1 (25.0%)	18 (75.0%)	15 (75.0%)
**Age**								
Mean (SD)	26.7 (8.12)	-	44.7 (11.2)	44.3 (8.05)	57.3 (12.8)	35.9 (4.45)	40.9 (16.6)	42.6 (8.12)
**Age groups**								
Under Age 30	9 (90.0%)	-	0 (0%)	1 (6.3%)	0 (0%)	0 (0%)	9 (37.5%)	1 (5.0%)
Age 30–50	1 (100%)	-	5 (71.4%)	11 (68.8%)	3 (42.9%)	4 (100%)	9 (37.5%)	15 (75.0%)
Age 51 and above	0 (0%)	-	2 (28.6%)	4 (25.0%)	4 (57.1%)	0 (0%)	6 (25.0%)	4 (20.0%)
**Fasting windows**								
Mean (SD)	16.2 (0.632)	-	16.6 (1.90)	15.8 (2.05)	14.9 (1.57)	15.0 (2.00)	15.9 (1.50)	15.6 (2.01)
**Fasting weeks**								
Mean (SD)	10.4 (13.7)	-	8.57 (2.51)	14.0 (9.85)	10.1 (7.03)	22.5 (18.6)	9.79 (9.43)	15.7 (12.0)
**Fasting weeks by groups**								
< 4 weeks	5 (50.0%)	-	0 (0%)	0 (0%)	2 (28.6%)	0 (0%)	7 (29.2%)	0 (0%)
5–8 weeks	2 (20.0%)	-	5 (71.4%)	4 (25.0%)	2 (28.6%)	1 (25.0%)	9 (37.5%)	5 (25.0%)
9–12 weeks	2 (20.0%)	-	2 (28.6%)	8 (50.0%)	2 (28.6%)	1 (25.0%)	6 (25.0%)	9 (45.0%)
> 12 weeks	1 (10.0%)	-	0 (0%)	4 (25.0%)	1 (14.3%)	2 (50.0%)	2 (8.3%)	6 (30.0%)
**Fast Time**								
Unrestricted frame time fasting	3 (30.0%)	-	3 (42.9%)	2 (12.5%)	2 (28.6%)	1 (25.0%)	8 (33.3%)	3 (15.0%)
Overnight fasting	7 (70.0%)	-	4 (57.1%)	14 (87.5%)	5 (71.4%)	2 (50.0%)	16 (66.7%)	16 (80.0%)
Daytime fasting	0 (0%)	-	0 (0%)	0 (0%)	0 (0%)	1 (25.0%)	0 (0%)	1 (5.0%)

Abbreviations: SD, standard deviation; N, number; kg, kilogram; (-), not applicable; %, percentage.

### The effect of time-restricted eating on weight loss in general

**Fig 2:**
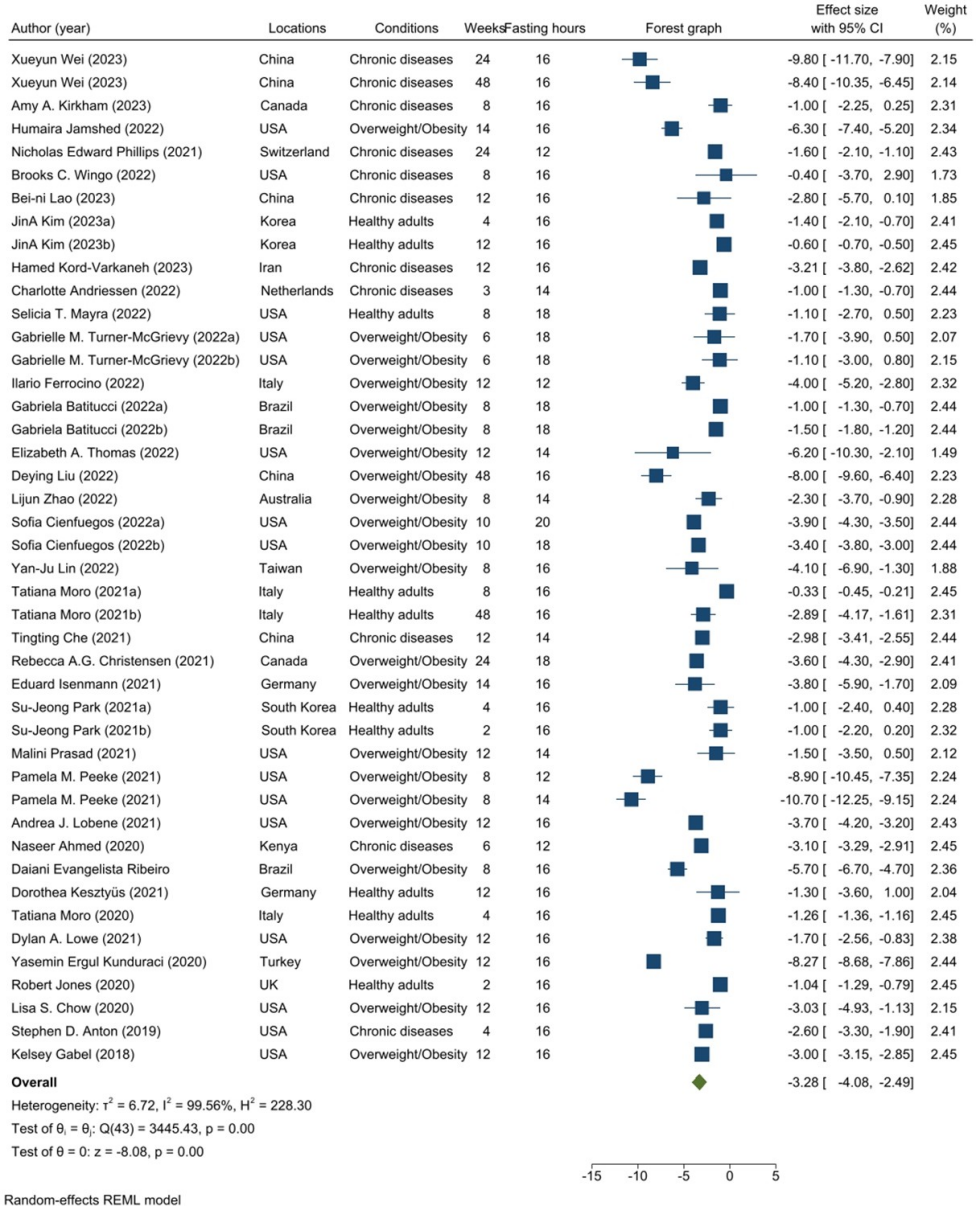
Random meta-analysis of the effects of TRE on weight loss in multiple intervention groups.

In aggregate, a total of 36 trials, comprising 44 effect sizes, were encompassed in the primary meta-analyses investigating the alterations in weight from baseline to the 48-week mark. Employing a random-effects model, our finding of the meta-analyses reveals a statistically significant overall reduction in weight within the intervention group of the TRE, with a mean difference of -3.28 Kg (95% CI: -4.08, -2.49; I^2^ = 99.56%, P < 0.001; ***Fig 2***) observed across diverse participant cohorts. Remarkably, all studies consistently reported the significant efficacy of the TRE intervention, resulting in varying degrees of weight loss among participants. The application of leave-one-out analyses, wherein each study was systematically excluded from the dataset, did not yield any significant impact on the overall effect observed (pooled weight loss ranging from −3.11 to −3.34, p < 0.001, ***S1 Fig***). The visual examination of the funnel plot ***(refer to S2 Fig)*** did not exhibit significant asymmetry. Moreover, the assessment of potential bias through the utilization of Egger’s test (p = 0.1274) and Begg’s test (p = 0.8634) yielded outcomes supporting the absence of publication bias within the meta-analyses examining the effects of the TRE on weight loss.

### Subgroups characterized by characteristics of participation

To elucidate more profound and robust conclusions, we undertook a systematic meta-analyses that scrutinized heterogeneity by comparing distinct subgroups categorized based on participant characteristics and the application of the TRE within the intervention group. Statistical assessments for subgroup differences based on health conditions and weight loss outcomes provided compelling evidence suggesting that the effectiveness of the TRE varies significantly across different health conditions (***refer to S3 Fig***). Notably, healthy individuals exhibited the most modest degree of weight loss, with a pooled effect size of -1.04 Kg (95% CI: -1.42, -0.65; I^2^ = 95.24%, P < 0.001). In contrast, participants afflicted with chronic diseases or those with overweight/obesity experienced more pronounced improvements in weight reduction, as evidenced by a pooled effect size of -3.33 Kg (95% CI: -5.05, -1.62; I^2^ = 99.11%, P < 0.001) and -4.21 Kg (95% CI: -5.23, -3.10; I^2^ = 98.88%, p < 0.001) respectively. These findings underscore the potential relevance of health status in influencing the efficacy of the TRE intervention. Furthermore, we identified that Asian participants exhibited a higher magnitude of weight reduction compared to their non-Asian counterparts, as evidenced by the pooled effect sizes of -3.87 Kg (95% CI: -5.82, -1.92, I^2^ = 98.93%, P < 0.001) and -3.10 Kg (95% CI: -3.96, -2.24, I^2^ = 99.56%, P < 0.001), respectively (***refer to S4 Fig***).

### Subgroups characterized by the TRE

In relation to the duration of the TRE, our findings indicate a positive correlation between the length of time following the diet and the magnitude of weight loss achieved. Specifically, we categorized the intervention period into four intervals: ≤4 weeks, 5–8 weeks, 9–12 weeks, and >12 weeks. The corresponding pooled effect sizes demonstrated an escalating pattern of impact, with values of -1.33 Kg (95% CI: -1.74, -0.92, I^2^ = 87.58%, P < 0.001), -3.08 (95% CI: -4.78, -1.38, I^2^ = 99.55%, P < 0.001), -3.27 Kg (95% CI: -4.26, -2.28, I^2^ = 99.01%, P < 0.001), and -5.48 Kg (95% CI: -7.54, -3.41, I^2^ = 95.55%, P < 0.001), respectively. These results highlight the progressive increase in the weight loss effect as the duration of the TRE extends (***refer to S5 Fig***). However, in the analysis of fasting durations throughout the day, it was revealed that fasting periods of 12 and 14 hours exhibited the most pronounced weight loss effects, as evidenced by the significant pooled effect sizes of -4.34 (95% CI: -7.38, -1.30, I^2^ = 99.07%, P < 0.001) and -4.02 (95% CI: -7.02, -1.00, I^2^ = 98.94%, P < 0.001) respectively. These outcomes suggest that relatively shorter fasting durations offer superior efficacy in promoting weight loss compared to extended fasting periods, encompassing intervals of 16 hours, 18 hours, and 20 hours. Ultimately, our analysis of various fasting regimens shows that individuals adhering to overnight fasting exhibited a more weight loss effect than those engaging in unrestricted frame time fasting or daytime fasting. Specifically, our findings revealed substantial pooled effect sizes of -3.76 (95% CI: -4.80, -2.71, I^2^ = 99.69%, P < 0.001), -2.02 (95% CI: -2.66, -1.37, I^2^ = 90.80%, P < 0.001), and -3.10 (95% CI: -3.29, -2.91, I^2^ = 99.69%, P < 0.01) for overnight fasting, unrestricted frame time fasting, and daytime fasting, respectively (***refer to S6 Fig***). These findings underscore the advantageous weight loss outcomes associated with the structured implementation of overnight fasting as a time-restricted approach. Interestingly, the included studies designed as RCTs exhibited higher pooled effect sizes of -3.9 kg (95% CI: -4.94, -2.87, I^2^ = 99.62%, P < 0.001) compared to non-RCTs, which had pooled effect sizes of -1.83 kg (95% CI: -2.48, -1.18, I^2^ = 94.44%, P < 0.001), (***refer to S7 Fig***).

### Subgroups characterized by the energy intake (calories) and macronutrient compositions (%)

**Fig 3:**
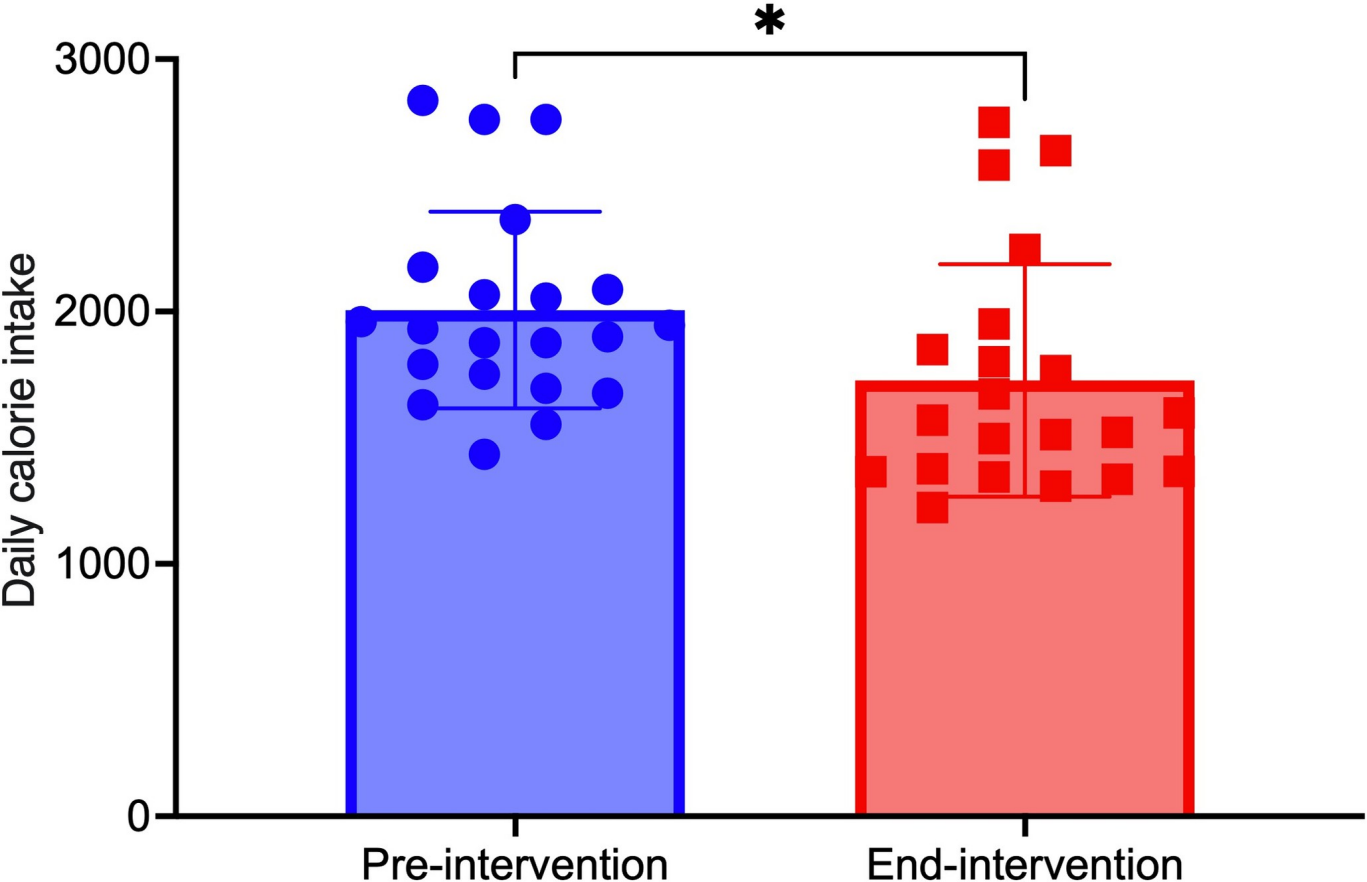
Comparative analysis of caloric intake between baseline and end of intervention (*p < 0.05, ^ns^ Non-significant results).

**Fig 4:**
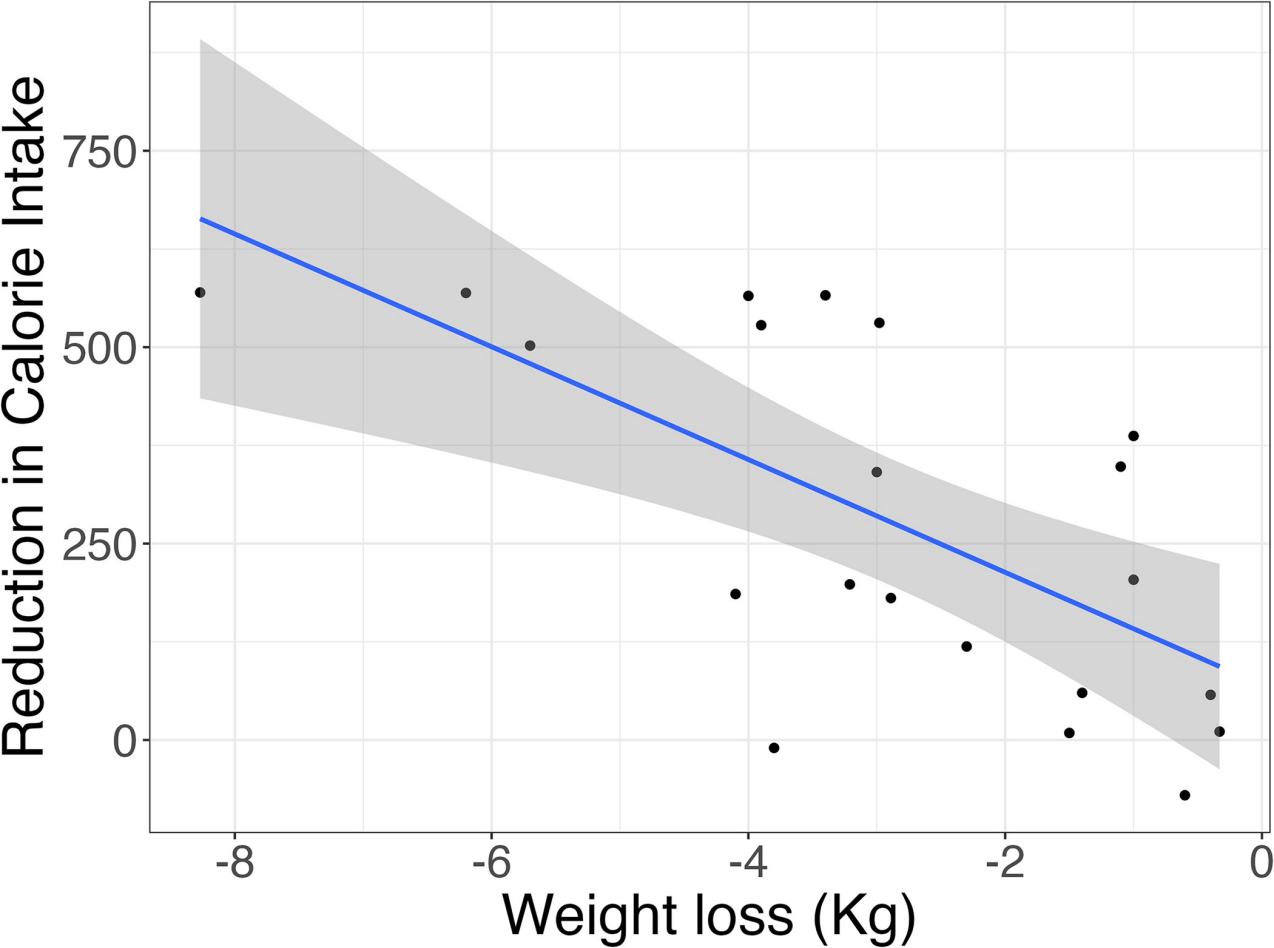
Univariate linear regression analysis of caloric intake in relation to mean differences in weight loss.

**Fig 5:**
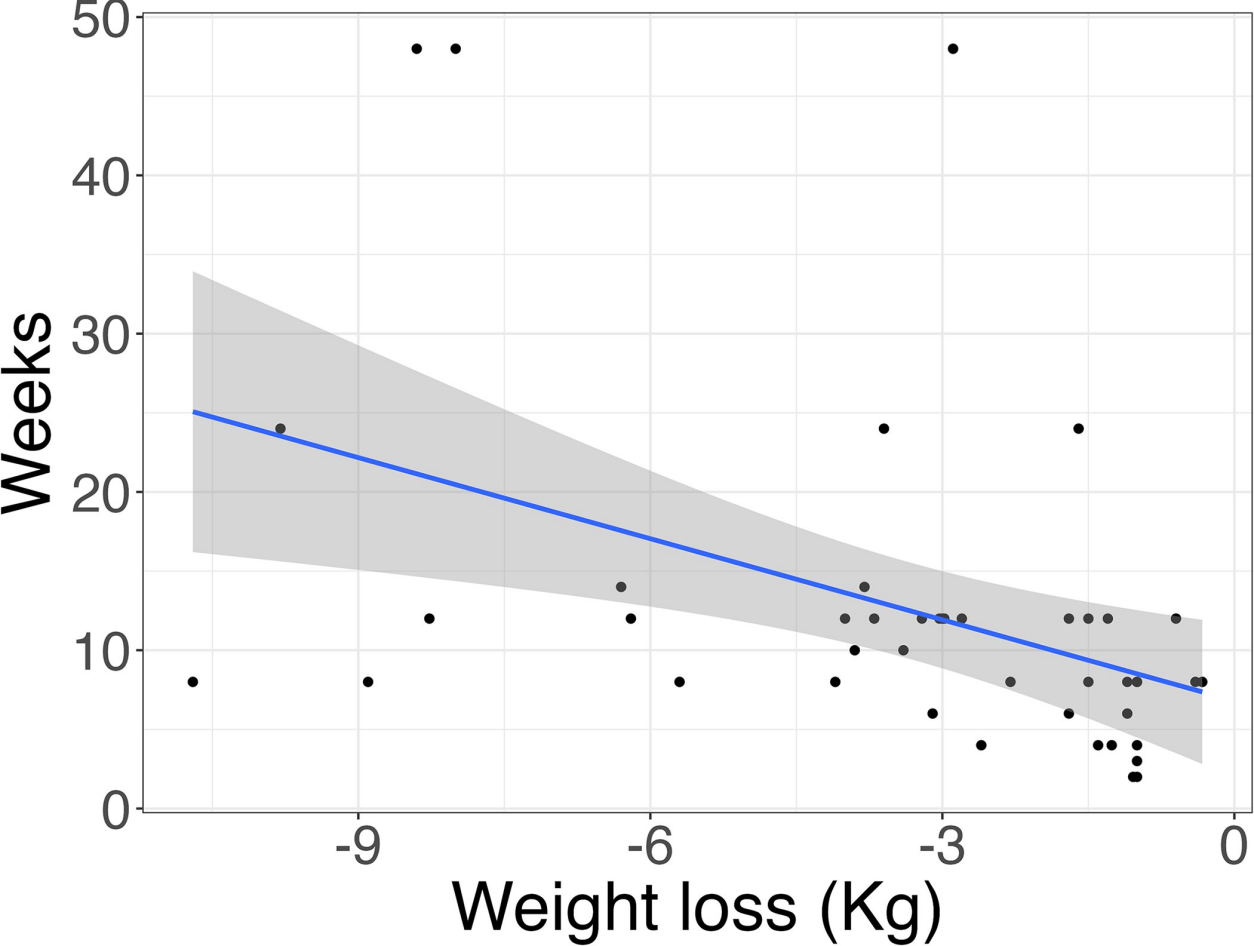
Univariate linear regression analysis of trial duration (in weeks) in relation to mean differences in weight loss.

Given the variability in food intake assessment methodologies and the considerable divergence in dietary patterns across different countries, we exclusively relied on pre- and post-intervention data regarding caloric and macronutrient composition to ascertain the efficacy of the TRE. The assessment of energy intake was conducted at both baseline and the end of the intervention in 17 studies with 21 effect sizes [23, 26, 28, 29, 33–40, 42, 48, 52, 56], which included a total of 365 participants with a mean age of 42.75±5.59 years. Of these, 13 studies were characterized by an ad libitum approach, indicating no requirement for participants to restrict energy intake based on eating timing [23, 26, 28, 29, 31, 34, 36–40, 42, 56], while four studies overtly implemented energy intake restrictions [33, 35, 48, 52]. The duration of these studies varied from 4 weeks to 48 weeks, with an average study duration of 11.5 weeks. In a random effects meta-analysis of eligible studies with available outcome data, our findings indicated a notable disparity in calorie intake between the end of the intervention and the baseline period. Specifically, the mean calorie intake at the end of intervention was pooled to be 1694.71 (95%CI: 1498.57, 1890.85, I^2^ = 82.07%, P<0.001), which was significantly lower than the baseline intake of 2000.64 (95%CI: 18230, 2172.98, I^2^ = 64.19%, P<0.001) (***refer to Figs 3 and S8***). A statistically significant correlation was observed between the extent of calorie intake reduction, the intervention weeks and the resulting weight loss magnitude (***refer to Figs 4 and 5)***. Despite the exclusion of the four studies that implemented calorie intake reduction in conjunction with the TRE, the weight loss outcomes in the remaining 13 studies exhibited a sustained statistical significance, as indicated by a P-value of less than 0.001 (***refer to S9 Fig)***. As participants progressively decreased their calorie intake and adhered to the TRE for an extended duration, a corresponding increase in weight loss was observed (P<0.05 and P<0.01, respectively). Additionally, we found a significant statistical relationship indicating that older individuals experienced greater weight loss effects with the TRE compared to their younger counterparts (P<0.05) (***refer to S9 Fig***).

Our analysis revealed a substantial reduction in calorie intake among participants who achieved a weight loss of 3 Kg or more (401 ± 210 kcal), whereas those with a weight loss of less than 3 Kg exhibited a comparatively more minor decrease in calorie intake (167 ± 186 kcal) at the end of the intervention (***refer to S3 Table***). Consistently, similar trends were observed in the overweight/obesity group, where a decline in calorie intake was evident, amounting to 424 ± 210 and 111 ± 97.8, respectively. Contrarily, among individuals with chronic diseases, those who achieved a weight loss of 3 Kg or more exhibited a lesser reduction in calorie intake (198 kcal) in comparison to those who lost less than 3 Kg (325 ± 243 kcal). Ultimately, among the healthy participants who adopted the TRE, their weight loss remained below the 3 Kg threshold, with the smallest decrease in calorie intake when contrasted with the other two groups.

**Fig 6:**
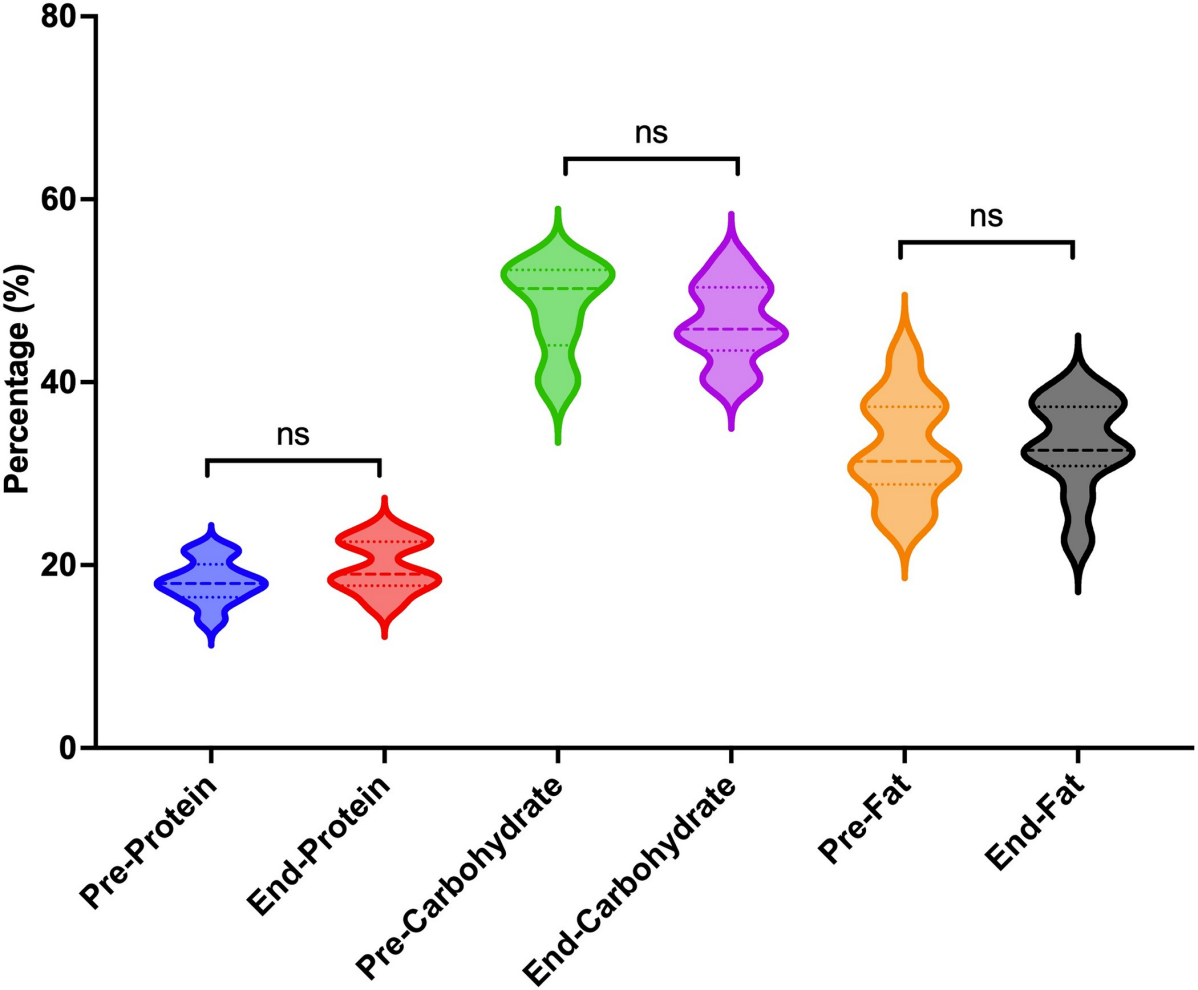
Comparative analysis of macronutrient compositions between baseline and end of intervention (*p < 0.05, ^ns^ Non-significant results).

With respect to the alteration of macronutrient compositions, our analysis unveiled no statistically significant disparity in the percentages of protein, carbohydrate, and fat before and after the intervention (***refer to Figs 6 and S10***) [23, 26, 28, 33, 39, 40, 52, 56].

### Risk of bias assessment

**Fig 7:**
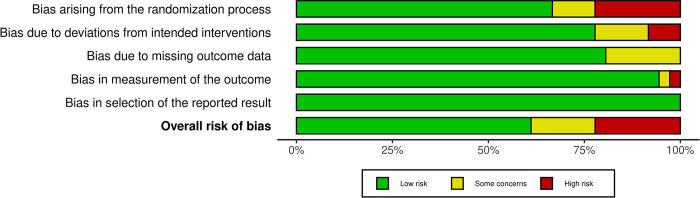
Risk of bias summary.

**Fig 8:**
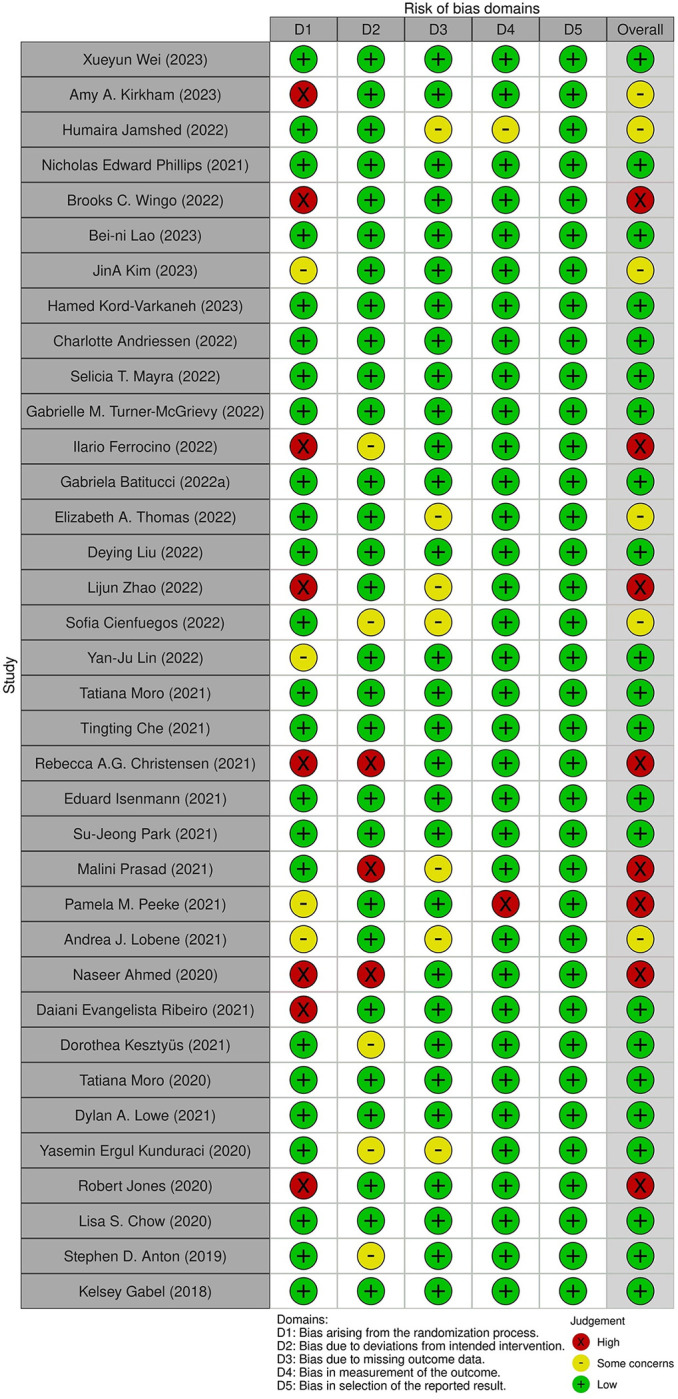
Quality assessment of the included study in meta-analysis.

***Fig 7*** depicts a graphical representation summarizing the risk of bias. ***Fig 8 and S4 Table*** illustrates the methodology employed to map bias judgments within domains to an overall judgment for the result. Most of the overall risk of bias aligns with the most critical risk of bias identified among any of the domains. Across most studies, a low risk of bias per domain was observed; however, six studies were found to have some concerns overall, while eight were deemed high risk.

### Adverse events

Of the 36 included studies, 14 reported on adverse effects, with none indicating any deaths during the intervention period. Furthermore, eight out of the 14 studies indicated that no adverse events were attributable to the intervention [30, 33, 37, 39, 40, 45–47]. Among the remaining six studies, the reported adverse events were generally minor, infrequent, and did not require medical intervention [22]. Mild symptoms, such as changes in appetite, stomach discomfort, dizziness, and fatigue, were observed at similar rates across both groups and resolved without the need for treatment [21]. Additional symptoms, including gas and bloating (33%), headaches (27%), light-headedness (23%), and hunger, were also reported but were mild and transient [23]. In one case, a patient with stage 4 diabetic nephropathy experienced a mild hypoglycemic episode, which was quickly managed with food [27]. Moreover, symptoms such as hunger, cravings, dizziness, and other side effects were typically mild and showed improvement over time [49, 55].

## Discussion

In the pursuit of improving population health, governments, researchers, and healthcare providers worldwide strive to identify the most effective weight-loss strategies. To the best of our knowledge, this study represents the inaugural systematic review and meta-analyses focused on investigating the weight loss outcome among groups in interventions involving TRE. In the findings, our meta-analyses of 36 prospective studies involving 914 participants revealed that while the TRE is generally effective in general adult populations, its efficacy varies among individuals with specific health conditions.

Consistent with earlier meta-analyses on TRE [10, 57, 58], our results corroborate the effectiveness of the TRE in weight management. Distinctively, our analysis reveals a pronounced average weight loss of 3.28 Kg due to the TRE, surpassing the smaller decreases observed in the aforementioned studies, specifically -1.60 Kg [57], 2.30 Kg [58], and -1.48 Kg [10]. The discrepancies in results between our study and prior literature are likely attributable to varied methodological frameworks. First, earlier reviews predominantly concentrated on a constrained assortment of study designs, specifically targeting weight loss maintenance through RCTs [10, 57]. Moreover, these studies typically imposed stringent inclusion criteria, focusing on specific conditions such as overweight or obesity [10], and their follow-up durations were often clinically limited, usually not less than 4 weeks [57]. Conversely, our systematic review encompassed a broader spectrum of study designs and inclusion criteria, thereby enhancing the generalizability of our analysis. Second, the studies included in our review exhibited substantial heterogeneity, mainly due to variations in the fasting time ranges and durations employed in TRE interventions. Viewed from this angle, these methodological differences may have influenced the outcomes to varying degrees. Nevertheless, despite this heterogeneity, the statistical significance of our results remained intact.

Clinical benefits are typically observed with a 3–5% reduction in body weight [17]. In terms of duration, our findings suggest that a minimum of five weeks of TRE strategy implementation is necessary to achieve a 3 Kg weight loss (e.g. 3.75% reduction in an 80 Kg adult), with longer periods resulting in greater reductions. Current meta-analyses yield conflicting conclusions regarding the correlation between the duration of TRE and its effectiveness in weight loss. For instance, Lili Liu’s (2022) study revealed that the TRE led to greater weight loss in the short term (<12 weeks) at -1.91 Kg (95% CI: -3.47 to -0.36) compared to the long term (≥12 weeks) at -1.29 Kg (95% CI: -1.96 to -0.61) [57]. Conversely, Jing-Chao Sun (2023) found that following TRE for a long-term period resulted in more significant weight loss (-1.46 kg, 95% CI: -1.89 to -1.03) compared to a short-term period (-0.88 kg, 95% CI: -2.19 to 0.42) [59]. In terms of fasting windows, our findings indicate that TRE regimens specifying nighttime fasting are associated with greater weight loss effectiveness compared to studies allowing participants to choose their TRE periods freely. Previous research indicates that the impact of TRF may vary based on the specific timing of food intake, although the exact time windows explored so far have not been clearly specified [6]. One study supports our findings that self-selected TRE periods are less effective at improving metabolic health markers [4]. However, the research on TRE with late-day eating shows inconsistent results. Some studies have highlighted benefits from late-day TRE, including reductions in body weight, insulin resistance, and oxidative stress [60]. However, other research suggests that these advantages may not be as pronounced as those observed with TRE conducted in the early or mid-day [6, 58]. In terms of health status, our analyses highlight a stratified effect of TRE on weight loss outcomes, with individuals presenting with chronic conditions and those classified within overweight or obese categories experiencing enhanced weight reduction in comparison to their normal-weight counterparts. Earlier meta-analyses support our results, showing TRE is an effective strategy for individuals with excess weight, as it significantly reduces body weight and improves metabolic parameters associated with cardiometabolic health. Moreover, other meta-analyses confirm that TRE promotes weight reduction in normal-weight individuals, though the improvements in obese individuals are not statistically significant [57]. Collectively, the variation in study durations and fasting windows from previous research demonstrates that TRE is effective over a wide range of time frames, showcasing its flexibility to adapt to individual health objectives and lifestyle choices. Given the lack of a universally optimal TRE guideline, this underscores the importance of personalized approaches based on individual preferences and health outcomes.

The concept of TRF for animals and TRE for humans stems from studies investigating meal timing effects on rodent circadian systems. Based on circadian rhythm theories, TRF enhances metabolism in animal models at least in part by acting through the molecular circadian clock [61]. This mechanism is under thorough investigation in animal models, with studies on TRE in humans now starting to emerge [62]. Restricting the eating window exhibits the potential to sustain circadian rhythms and enhance metabolism through the elongation of the daily fasting period [61, 63]. This, in turn, activates cellular pathways implicated in mediating the benefits associated with calorie restriction [61]. Indeed, previous research in animal models indicates that TRF durations of less than 8 hours can inadvertently reduce calorie intake in rodents [61]. However, even though TRE is effective for weight loss in humans, its impact on changing dietary patterns, especially in reducing calorie intake, remain to be fully elucidated [64]. Although the TRE does not specifically aim for calorie reduction [61, 65], our findings indicate that this diet might simultaneously lead to decreased overall calorie consumption and subsequent weight loss improvements. This result is observed in the methodology of the included studies, where participants were not explicitly instructed to cut calories. Nonetheless, participants reduced their caloric intake either unintentionally or voluntarily from the start to the conclusion of the study period, suggesting an indirect effect of TRE on energy consumption patterns. We theorize that the observed weight loss may be explained by the principle of negative energy balance, where weight loss initiates from reduced energy intake and/or increased energy expenditure. TRE appears to influence both components of this energy equation, thereby contributing to its effectiveness in weight loss [63, 66]. Consequently, forthcoming investigations are required to diligently assess eating behaviors, particularly focusing on the precise quantification of caloric intake changes throughout the intervention period.

### Strengths and limitations of this review

Our approach to conducting literature searches across multiple electronic databases was a strength, ensuring the inclusion of relevant studies and minimizing the risk of overlooking crucial evidence. Moreover, we employed validated tools to assess the methodological quality and evidence quality of all identified associations, thereby enhancing the reliability of our findings. Additionally, we conducted extensive subgroup and sensitivity analyses, which further strengthened the robustness of our analysis. To mitigate bias in our review, we implemented various measures, such as the recalculation of all meta-analyses using a random effect method and the independent conduct of the review process by two authors. Our study has also deepened the understanding of how the TRE facilitates weight loss, partly through the decrease in calorie intake. Leveraging these findings, future research could enhance the knowledge of TRE’s effectiveness and its role in weight management.

Our review is not without its limitations. Firstly, the differences between our findings and previous reviews may be due to the varied methodological approaches used. Previous reviews have narrowly focused on study designs such as RCTs targeting weight loss and metabolic-related parameters in adults [10, 57], limiting their inclusion criteria to specific health conditions like overweight or obesity [10] and requiring follow-up periods of at least 4 weeks [57]. In contrast, our systematic review includes a broader range of study designs and criteria, leading to a more comprehensive analysis. Specifically, there has been some controversy surrounding the use of single-group studies to assess effectiveness [67, 68], presenting a potential limitation in our study. Mowever, the inclusion of non-RCTs (e.g. pilot and feasibility studies) warrants careful interpretation. Compared to RCTs, these studies often have less robust designs, lack control groups, and typically involve smaller sample sizes, which can introduce biases and reduce the overall strength and generalizability of our findings. The inclusion of such studies may impact the reliability of the conclusions drawn about the efficacy and impact of TRE interventions. Furthermore, significant heterogeneity among the included studies, mainly due to varying fasting windows and durations in the TRE interventions, may have influenced the reported outcomes. Despite these limitations, the statistical significance of our results remains intact.

Secondly, in our analysis of dietary programs, a notable limitation arose in terms of systematically incorporating adherence to the TRE. This limitation primarily stemmed from inconsistent reporting practices observed among the 36 eligible trials, which employed different methods for measuring and reporting adherence. The lack of standardized reporting hindered our ability to precisely account for adherence as a variable in our analysis. Furthermore, the validity of measures used to assess adherence in dietary program trials has been subject to questioning. Particularly, when reliable indicators for adherence are absent or not utilized in the context of these trials, concerns arise regarding the accuracy and reliability of adherence measures. Consequently, effect sizes might have been diminished, leading to a potential underestimation of the true effects of the TRE when adherence was suboptimal. Future research endeavors should aim to address these limitations by implementing standardized methods for measuring and reporting adherence to the TRE, thus enabling a more comprehensive understanding of the relationship between adherence to the TRE and its effects on health outcomes.

Thirdly, the large included studies often rely on self-reported dietary data collected through instruments such as food frequency questionnaires, which may be prone to measurement error and can potentially diminish the ability to detect the energy intake and macronutrient compositions. Additionally, the majority of included studies in our analysis assessed dietary habits at a single time point, failing to account for potential changes in diet quality over time that may impact body weight outcomes during the implementation of the TRE.

Finally, it is imperative to acknowledge that the predominant proportion of studies encompassed in this review were conducted within high-income countries, thus curbing the external validity of the findings to low- and middle-income countries. As such, a judicious interpretation and cautious utilization of the meta-analyses estimates are warranted, given the aforementioned limitations.

### Implications

In light of recent evidence, practitioners are encouraged to employ a personalized approach when recommending TRE for weight management. While TRE’s effectiveness can differ among individuals, the majority of recent studies highlight its success in promoting weight loss, especially in patients with chronic conditions or overweight/obesity. It is vital, however, to approach TRE as part of a comprehensive lifestyle intervention, emphasizing the importance of balanced nutrition and regular physical activity. Regular monitoring and adjustments based on the patient’s response to the TRE are essential to ensure the safety and effectiveness of the dietary strategy.

For future research, a focus on understanding the effects of TRE across diverse populations, considering variables like age, sex, and health status, is warranted. Exploring the synergy between TRE and various dietary compositions is crucial, as it allows for an assessment of how different nutritional profiles influence TRE’s efficacy. Long-term studies are also essential to assess the prolonged adherence, feasibility, and extended health outcomes of TRE before proposing any updates to national and international dietary guidelines.

## Conclusions

In conclusion, the present review provides compelling evidence supporting the efficacy of TRE as a promising therapeutic approach for facilitating weight loss in individuals with overweight and obesity, and concurrently suggests its potential utility in promoting weight maintenance among individuals with a healthy weight profile. Although the mechanisms underlying TRE remain incompletely elucidated in extant literature, our review revealed that diminished energy intake may contribute, at least in part, to the heterogeneity in weight loss observed across diverse populations. Practitioners should customize TRE recommendations for weight management, especially for patients with chronic conditions or obesity, while emphasizing comprehensive lifestyle intervention, including nutrition, physical activity, and ongoing monitoring for safety and effectiveness. Finally, there is a clear need for more rigorous human studies to thoroughly investigate the effectiveness, safety, long-term adherence, underlying mechanisms, and sustainability of TRE in diverse demographic cohorts and a wide range of medical conditions.

## Supporting information

S1 Checklist PRISMA 2020 checklist. (DOCX)

S1 Table Search strategy for article selection. (DOCX)

S2 Table Characteristics of the 36 eligible studies included in the review. (DOCX)

S3 Table The alterations in daily caloric intake among adults following the TRE. (DOCX)

S4 Table List of articles identified in the literature search with reasons for inclusion and exclusion. (DOCX)

S5 Table Assessment of bias risk in randomized controlled trials included in the meta-analysis using the Cochrane risk of bias 2 tool. (DOCX)

S1 Fig Leave-one-out sensitivity analysis of the effect of time-restricted eating on weight loss in general adults. (DOCX)

S2 Fig Funnel plot of risk of publication bias for the effect of time-restricted eating on weight loss in general adults. (DOCX)

S3 Fig Subgroup analyses of the meta-analysis based on diverse health conditions. (DOCX)

S4 Fig Subgroup analyses of the meta-analysis based on locations. (DOCX)

S5 Fig Subgroup analyses of the meta-analysis based on intervention durations. (DOCX)

S6 Fig Subgroup analyses of the meta-analysis based on time fasting. (DOCX)

S7 Fig Subgroup analyses of the meta-analysis based on research designs. (DOCX)

S8 Fig Forest plot illustrating the pooled mean caloric intake at baseline and the conclusion of the tre intervention period. (DOCX)

S9 Fig Univariate linear regression analysis of caloric intake (in 13 studies characterized by an ad libitum approach) and mean age in relation to mean differences in weight loss. (DOCX)

S10 Fig Changes in macronutrient compositions from baseline to the conclusion of the tre intervention period among adult participants on the TRE. (DOCX)

## Abbreviations

WHO: World Health Organization
TRE: Time-restricted eating
TRF: Time-restricted feeding
RCTs: Randomized Controlled Trials
PRISMA: Preferred Reporting Items for Systematic Reviews and Meta-Analyses
PROSPERO: International Prospective Register of Systematic Reviews
CENTRAL: Cochrane Central Register of Controlled Trials
MeSH: Medical Subject Heading
CIs: Confidence intervals
SDs: Standard deviations
SEs: Standard errors
MDs: Mean differences
