# Supplementary material for: Partially unraveling mechanistic underpinning and weight loss effects of time-restricted eating across diverse adult populations: A systematic review and meta-analyses of prospective studies
Source: PLoS One. 2025 Jan 15;20(1):e0314685. doi: 10.1371/journal.pone.0314685 (PMC11734929; doi:10.1371/journal.pone.0314685)
Supplement: S1 Table — (DOCX) [file pone.0314685.s002.docx]

| **Supplementary S1.** Search strategy for article selection | | |
| --- | --- | --- |
| **Database** | **Search strategy** | **Number of results** |
| Pubmed | **("intermittent fasting" or "time restricted feeding" or "time restricted eating" or "alternate day fasting" or "time restricted feedings" or "time restricted fastings" or "time restricted fasting" or "time‐restricted feeding" or "time‐restricted diet" or "time‐restricted meal" or "time‐restricted fasting" or "time‐restricted eating") and ("body mass index" or "adiposity" or "overweight" or "obesity" or "weight" or "body weight") Filters: Humans, English, Exclude preprints**  (("intermittent fasting"[All Fields] OR "time-restricted feeding"[All Fields] OR "time-restricted eating"[All Fields] OR "alternate day fasting"[All Fields] OR "time restricted feedings"[All Fields] OR (("time"[MeSH Terms] OR "time"[All Fields]) AND ("restrict"[All Fields] OR "restricted"[All Fields] OR "restricting"[All Fields] OR "restriction"[All Fields] OR "restrictions"[All Fields] OR "restrictive"[All Fields] OR "restrictiveness"[All Fields] OR "restricts"[All Fields]) AND ("fasted"[All Fields] OR "fasting"[MeSH Terms] OR "fasting"[All Fields] OR "fastings"[All Fields] OR "fasts"[All Fields])) OR "time-restricted fasting"[All Fields] OR "time-restricted feeding"[All Fields] OR "time-restricted diet"[All Fields] OR ("time restricted"[All Fields] AND ("meals"[MeSH Terms] OR "meals"[All Fields] OR "meal"[All Fields])) OR "time-restricted fasting"[All Fields] OR "time-restricted eating"[All Fields]) AND ("body mass index"[All Fields] OR "adiposity"[All Fields] OR "overweight"[All Fields] OR "obesity"[All Fields] OR "weight"[All Fields] OR "body weight"[All Fields])) AND ((excludepreprints[Filter]) AND (humans[Filter]) AND (english[Filter])) | **298** |
| Scopus | TITLE-ABS-KEY(("intermittent fasting" or "time restricted feeding" or "time restricted eating" or "alternate day fasting" or "time restricted feedings" or "time restricted fastings" or "time restricted fasting" or "time‐restricted feeding" or "time‐restricted diet" or "time‐restricted meal" or "time‐restricted fasting" or "time‐restricted eating") and ("body mass index" or "adiposity" or "overweight" or "obesity" or "weight" or "body weight")) AND ( LIMIT-TO ( DOCTYPE,"ar" ) ) AND ( LIMIT-TO ( LANGUAGE,"English" ) ) AND ( LIMIT-TO ( EXACTKEYWORD,"Article" ) OR LIMIT-TO ( EXACTKEYWORD,"Human" ) ) AND ( LIMIT-TO ( PUBSTAGE,"final" ) ) | **896** |
| Web of Science | (“intermittent fasting” or “time restricted feeding” or “time restricted eating” or “alternate day fasting” or “time restricted feedings” or “time restricted fastings” or “time restricted fasting” or “time‐restricted feeding” or “time‐restricted diet” or “time‐restricted meal” or “time‐restricted fasting” or “time‐restricted eating”) and (“body mass index” or “adiposity” or “overweight” or “obesity” or “weight” or “body weight”) | **776** |
| Cochrane Central Register of Controlled Trials | (“intermittent fasting” or “time restricted feeding” or “time restricted eating” or “alternate day fasting” or “time restricted feedings” or “time restricted fastings” or “time restricted fasting” or “time‐restricted feeding” or “time‐restricted diet” or “time‐restricted meal” or “time‐restricted fasting” or “time‐restricted eating”) and (“body mass index” or “adiposity” or “overweight” or “obesity” or “weight” or “body weight”) | **604** |
| **Total** | | **2,574** |
