# Supplementary material for: Partially unraveling mechanistic underpinning and weight loss effects of time-restricted eating across diverse adult populations: A systematic review and meta-analyses of prospective studies
Source: PLoS One. 2025 Jan 15;20(1):e0314685. doi: 10.1371/journal.pone.0314685 (PMC11734929; doi:10.1371/journal.pone.0314685)
Supplement: S2 Table — (DOCX) [file pone.0314685.s003.docx]

**Supplementary S2.** Characteristics of the 36 Eligible Studies Included in the Review

| **Author (Year of publication)*** | **Conditions** | **Design** | **Duration** | **Weeks** | **Fasting hours** | **Night fasting** | **Sample size** | **Age** | **SD age** | **Effect (kg)** | **CI low** | **CI up** | **P value** |
| --- | --- | --- | --- | --- | --- | --- | --- | --- | --- | --- | --- | --- | --- |
| Xueyun Wei (2023) [1] | Chronic diseases | Randomized clinical trial | 6 months | 24 | 16 | Night | 45 | 32.3 | 10.5 | -9.8 | -11.7 | -7.9 | 0.94 |
| Xueyun Wei (2023) [1] | Chronic diseases | Randomized clinical trial | 12 months | 48 | 16 | Night | 45 | 32.3 | 10.5 | -8.4 | -10.3 | -6.4 | 0.69 |
| Amy A. Kirkham (2023) [2] | Chronic diseases | A single-arm feasibility study | 8 weeks | 8 | 16 | Night | 22 | 66 | 5 | -1 | -2.3 | 0.2 | N/A |
| Humaira Jamshed (2022) [3] | Overweight/Obesity | Parallel-arm randomized controlled trial | 14 weeks | 14 | 16 | Night | 29 | 45 | 10 | -6.3 | -7.4 | -5.2 | P < .001 |
| Nicholas Edward Phillips (2021) [4] | Chronic diseases | A pragmatic randomised controlled trial | 6 months | 24 | 12 | Unrestricted frame time fasting | 28 | ﻿44.3 | ﻿12.8 | -1.6 | -2.1 | -1.1 | 0.01 |
| Brooks C. Wingo (2022) [5] | Chronic diseases | Prospective single-group pilot study | 8 weeks | 8 | 16 | Night | 12 | 46 | 10 | -0.4 | -3.3 | 3.3 | 0.12 |
| Bei-ni Lao (2023) [6] | Chronic diseases | A prospective, non-randomized controlled short-term clinical trial | 12 weeks | 12 | 16 | Night | 13 | 51.8 | 7.7 | -2.8 | -5.7 | 0.1 | 0.004 |
| JinA Kim (2023a) [7] | Healthy adults | A single-arm feasibility study | 4 weeks | 4 | 16 | Night | 21 | 23.4 | 2.9 | -1.4 | -2.1 | -0.7 | <0.0001 |
| JinA Kim (2023b) [7] | Healthy adults | A single-arm feasibility study | 4 weeks | 12 | 16 | Night | 13 | 23.4 | 2.9 | -0.6 | -0.7 | -0.5 | 0.2109 |
| Hamed Kord-Varkaneh (2023) [8] | Chronic diseases | A randomized clinical trial | 12 weeks | 12 | 16 | Unrestricted frame time fasting | 22 | 41.36 | 10.5 | -3.21 | -3.8 | -2.62 | ˂0.001 |
| Charlotte Andriessen (2022) [9] | Chronic diseases | A randomised crossover study | 3 weeks | 3 | 14 | Night | 14 | 67.5 | 5.2 | -1 | -1.3 | -0.7 | p<0.01 |
| ﻿Selicia T. Mayra (2022) [10] | Healthy adults | ﻿A randomized study | 8 weeks | 8 | 18 | Night | 8 | 25.1 | 4.1 | -1.1 | -2.7 | 0.5 | ﻿0.016 |
| Gabrielle M. Turner-McGrievy (2022a) [11] | Overweight/Obesity | ﻿A randomized study | 6 weeks | 6 | 18 | Unrestricted frame time fasting | 29 | 45.7 | 12.2 | -1.7 | -3.9 | 0.5 | 0.157 |
| Gabrielle M. Turner-McGrievy (2022b) [11] | Overweight/Obesity | ﻿A randomized study | 6 weeks | 6 | 18 | Night | 33 | 45.7 | 12.2 | -1.1 | -3 | 0.8 | 0.157 |
| Ilario Ferrocino (2022) [12] | Overweight/Obesity | A non-randomized controlled study | 12 weeks | 12 | 12 | Night | 25 | 58.6 | 7.9 | -4 | -4.7 | -2.3 | 0.038 |
| Gabriela Batitucci (2022a) [13] | Overweight/Obesity | A randomized study | 8 weeks | 8 | 18 | Unrestricted frame time fasting | 10 | 30 | 5 | -1 | -1.3 | -0.7 | N/A |
| Gabriela Batitucci (2022b) [13] | Overweight/Obesity | A randomized study | 8 weeks | 8 | 18 | Unrestricted frame time fasting | 15 | 32 | 4.4 | -1.5 | -1.8 | -1.2 | N/A |
| Elizabeth A. Thomas (2022) [14] | Overweight/Obesity | A randomized trial | 12 weeks | 12 | 14 | Night | 33 | 38.3 | 7.9 | -6.2 | -10.3 | -2.1 | p<0.001 |
| Deying Liu (2022) [15] | Overweight/Obesity | A randomized trial | 12 months | 48 | 16 | Night | 69 | 31.6 | 9.3 | -8 | -9.6 | -6.4 | 0.11 |
| Lijun Zhao (2022) [16] | Overweight/Obesity | A single-arm, pre-post trial | 8 weeks | 8 | 14 | Night | 15 | 63 | 4 | -2.3 | -3.7 | -0.9 | 0.015 |
| Sofia Cienfuegos (2022a) [17] | Overweight/Obesity | A randomized parallel-arm trial | 10 weeks | 10 | 20 | Night | 16 | 49 | 2 | -3.9 | -4.3 | -3.5 | N/A |
| Sofia Cienfuegos (2022b) [17] | Overweight/Obesity | A randomized parallel-arm trial | 10 weeks | 10 | 18 | Night | 19 | 46 | 3 | -3.4 | -3.8 | -3 | N/A |
| Yan-Ju Lin (2022) [18] | Overweight/Obesity | A single-center, randomized, open-label, parallel-group design. | 8 weeks | 8 | 16 | Night | 30 | 50.1 | 7.5 | -4.1 | -6.9 | -1.3 | 0.012 |
| Tatiana Moro (2021a) [19] | Healthy adults | A single-blind randomized study | 2 months | 8 | 16 | Night | 10 | 29.94 | 4.07 | -0.33 | -0.45 | -0.21 | N/A |
| Tatiana Moro (2021b) [19] | Healthy adults | A single-blind randomized study | 12 months | 48 | 16 | Night | 10 | 29.94 | 4.07 | -2.89 | -4.17 | -1.61 | N/A |
| Tingting Che (2021) [20] | Chronic diseases | A randomised controlled trial | 12 weeks | 12 | 14 | Night | 60 | 48.21 | 9.32 | -2.98 | -3.41 | -2.55 | < 0.001 |
| Rebecca A.G. Christensen (2021) [21] | Overweight/Obesity | A unidirectional case crossover design | 6 months | 24 | 18 | Night | 83 | 51.2 | 0.8 | -3.6 | -4.3 | -2.9 | P < 0.05 |
| Eduard Isenmann (2021) [22] | Overweight/Obesity | A randomised controlled trial | 14 weeks | 14 | 16 | Night | 18 | 27.9 | 5.3 | -3.8 | -5.9 | -1.7 | < 0.0001 |
| Su-Jeong Park (2021a) [23] | Healthy adults | A feasibility study | 4 weeks | 4 | 16 | Unrestricted frame time fasting | 33 | 22.5 | 2.8 | -1 | -2.4 | 0.4 | 0.0001 |
| Su-Jeong Park (2021b) [23] | Healthy adults | A feasibility study | 2 weeks | 2 | 16 | Unrestricted frame time fasting | 33 | 22.5 | 2.8 | -1 | -2.2 | 0.2 | <0.0001 |
| Malini Prasad (2021) [24] | Overweight/Obesity | An open label, non-randomized, prospective intervention | 12 weeks | 12 | 14 | Night | 28 | 50 | 11 | -1.5 | -3.5 | 0.5 | 0.017 |
| Pamela M. Peeke (2021) [25] | Overweight/Obesity | A randomized, comparator-controlled, clinical trial | 8 weeks | 8 | 12 | Night | 30 | 44 | 11 | -8.9 | -10.4 | -7.3 | <0.0001 |
| Pamela M. Peeke (2021) [25] | Overweight/Obesity | A randomized, comparator-controlled, clinical trial | 8 weeks | 8 | 14 | Night | 30 | 44 | 11 | -10.7 | -12.3 | -9.2 | <0.0001 |
| Andrea J. Lobene (2021) [26] | Overweight/Obesity | A randomized controlled trial | 12 weeks | 12 | 16 | Unrestricted frame time fasting | 11 | 46.5 | 3.7 | -3.7 | -4.2 | -3.2 | < 0.05 |
| Naseer Ahmed (2020) [27] | Chronic diseases | A quasi-experimental clinical study | 6 weeks | 6 | 12 | Daytime | 15 | 37.8 | 12.25 | -3.1 | -3.29 | -2.91 | 0.0001 |
| Daiani Evangelista Ribeiro (2021) [28] | Overweight/Obesity | A randomized study | 8 weeks | 8 | 16 | Night | 8 | 32.4 | 5.5 | -5.7 | -6.7 | -4.7 | N/A |
| Dorothea Kesztyüs (2021) (29) | Healthy adults | A pre-post design | 3 months | 12 | 16 | Unrestricted frame time fasting | 63 | 47.8 | 10.5 | -1.3 | -3.6 | 1 | N/A |
| Tatiana Moro (2020) (30) | Healthy adults | A randomized controlled trial | 4 weeks | 4 | 16 | Night | 8 | 19.38 | 2.39 | -1.26 | -1.36 | -1.16 | N/A |
| ﻿Dylan A. Lowe (2021) (31) | Overweight/Obesity | A randomized controlled trial | 12 weeks | 12 | 16 | Night | 22 | 46.8 | 10.8 | -1.7 | -2.56 | -0.83 | N/A |
| Yasemin Ergul Kunduraci (2020) (32) | Overweight/Obesity | A randomized controlled trial | 12 weeks | 12 | 16 | Night | 32 | 47.44 | 2.17 | -8.27 | -8.68 | -7.86 | 0.029 |
| Robert Jones (2020) (33) | Healthy adults | A randomized controlled trial | 2 weeks | 2 | 16 | Night | 8 | 23 | 1 | -1.04 | -1.29 | -0.79 | 0.01 |
| Lisa S. Chow (2020) (34) | Overweight/Obesity | A Feasibility Study | 12 weeks | 12 | 16 | Unrestricted frame time fasting | 11 | 46.5 | 12.4 | -3.03 | -4.93 | -1.13 | ﻿<.0001 |
| Stephen D. Anton (2019) (35) | Chronic diseases | A Pilot Study | 4 weeks | 4 | 16 | Unrestricted frame time fasting | 10 | 77.1 | N/A | -2.6 | -3.3 | -1.9 | 0.009 |
| Kelsey Gabel (2018) (36) | Overweight/Obesity | A randomized controlled trial | 12 weeks | 12 | 16 | Night | 23 | 50 | 2 | -3 | -3.15 | -2.85 | <0.001 |
| Abbreviations: SD: Standard deviation; CIs = confidence intervals.  * Name of data extractors: LCL, PNQ, NDK, and VTQC. The included studies were extracted between February 5 and March 15, 2024. | | | | | | | | | | | | | |

**References**

1. Wei X, Lin B, Huang Y, Yang S, Huang C, Shi L, et al. Effects of Time-Restricted Eating on Nonalcoholic Fatty Liver Disease: The TREATY-FLD Randomized Clinical Trial. JAMA Netw Open. 2023;6: E233513. doi:10.1001/jamanetworkopen.2023.3513

2. Kirkham AA, Ford KL, Ramos Da Silva B, Topolnyski J, Prado CM, Joy AA, et al. Implementation of weekday time-restricted eating to improve metabolic health in breast cancer survivors with overweight/obesity. Obesity. 2023;31: 150–160. doi:10.1002/oby.23654

3. Jamshed H, Steger FL, Bryan DR, Richman JS, Warriner AH, Hanick CJ, et al. Effectiveness of Early Time-Restricted Eating for Weight Loss, Fat Loss, and Cardiometabolic Health in Adults With Obesity A Randomized Clinical Trial. JAMA Intern Med. 2022;182: 953–962. doi:10.1001/jamainternmed.2022.3050

4. Phillips NE, Mareschal J, Schwab N, Manoogian ENC, Borloz S, Ostinelli G, et al. The Effects of Time-Restricted Eating versus Standard Dietary Advice on Weight, Metabolic Health and the Consumption of Processed Food: A Pragmatic Randomised Controlled Trial in Community-Based Adults. Nutrients. 2021;13. doi:10.3390/nu13031042 WE  - Science Citation Index Expanded (SCI-EXPANDED)

5. Wingo BC, Rinker JR 2nd, Green K, Peterson CM. Feasibility and acceptability of time-restricted eating in a group of adults with  multiple sclerosis. Front Neurol. 2022;13: 1087126. doi:10.3389/fneur.2022.1087126

6. Lao BN, Luo JH, Xu XY, Fu LZ, Tang F, Ouyang WW, et al. Time-restricted feeding’s effect on overweight and obese patients with chronic kidney disease stages 3-4: A prospective non-randomized control pilot study. Front Endocrinol (Lausanne). 2023;14. doi:10.3389/fendo.2023.1096093 WE  - Science Citation Index Expanded (SCI-EXPANDED)

7. Kim J, Song Y. Early Time-Restricted Eating Reduces Weight and Improves Glycemic Response in Young Adults: A Pre-Post Single-Arm Intervention Study. Obes Facts. 2023;16: 69–81. doi:10.1159/000527838

8. Kord-Varkaneh H, Salehi-Sahlabadi A, Tinsley GM, Santos HO, Hekmatdoost A. Effects of time-restricted feeding (16/8) combined with a low-sugar diet on the management of non-alcoholic fatty liver disease: A randomized controlled trial. NUTRITION. 2023;105. doi:10.1016/j.nut.2022.111847 WE  - Science Citation Index Expanded (SCI-EXPANDED)

9. Andriessen C, Fealy CE, Veelen A, van Beek SMM, Roumans KHM, Connell NJ, et al. Three weeks of time-restricted eating improves glucose homeostasis in adults with type 2 diabetes but does not improve insulin sensitivity: a randomised crossover trial. Diabetologia. 2022;65: 1710–1720. doi:10.1007/s00125-022-05752-z

10. Mayra ST, Chondropoulos K, De Leon A, Kravat N, Johnston CS. The feasibility and preliminary efficacy of early time-restricted eating on diet quality in college students: A randomized study. Obes Res Clin Pract. 2022;16: 413–420. doi:10.1016/j.orcp.2022.08.009

11. Turner-McGrievy GM, Wirth MD, Bernhart JA, Aydin H. The Fasting and Shifted Timing (FAST) of Eating Study: A pilot feasibility randomized crossover intervention assessing the acceptability of three different fasting diet approaches. Appetite. 2022;176. doi:10.1016/j.appet.2022.106135

12. Ferrocino I, Pelleagrini M, D’eusebio C, Goitre I, Ponzo V, Fadda M, et al. The Effects of Time‐Restricted Eating on Metabolism and Gut Microbiota: A Real‐Life Study. Nutrients. 2022;14. doi:10.3390/nu14132569

13. Batitucci G, Faria Junior E V, Nogueira JE, Brandão CFC, Abud GF, Ortiz GU, et al. Impact of Intermittent Fasting Combined With High-Intensity Interval Training on Body Composition, Metabolic Biomarkers, and Physical Fitness in Women With Obesity. Front Nutr. 2022;9. doi:10.3389/fnut.2022.884305

14. Thomas EA, Zaman A, Sloggett KJ, Steinke S, Grau L, Catenacci VA, et al. Early time-restricted eating compared with daily caloric restriction: A randomized trial in adults with obesity. Obesity. 2022;30: 1027–1038. doi:10.1002/oby.23420

15. Liu DY, Huang Y, Huang CSH, Yang SY, Wei XY, Zhang PZ, et al. Calorie Restriction with or without Time-Restricted Eating in Weight Loss. NEW ENGLAND JOURNAL OF MEDICINE. 2022;386: 1495–1504. doi:10.1056/NEJMoa2114833 WE  - Science Citation Index Expanded (SCI-EXPANDED)

16. Zhao L, Hutchison AT, Liu B, Yates CL, Teong XT, Wittert GA, et al. Time-restricted eating improves glycemic control and dampens energy-consuming pathways in human adipose tissue. Nutrition. 2022;96. doi:10.1016/j.nut.2021.111583

17. Cienfuegos S, Gabel K, Kalam F, Ezpeleta M, Pavlou V, Lin S, et al. The effect of 4-h versus 6-h time restricted feeding on sleep quality, duration, insomnia severity and obstructive sleep apnea in adults with obesity. Nutr Health. 2022;28: 5–11. doi:10.1177/02601060211002347

18. Lin YJ, Wang YT, Chan LC, Chu NF. Effect of time-restricted feeding on body composition and cardiometabolic risk in middle-aged women in Taiwan. NUTRITION. 2022;93. doi:10.1016/j.nut.2021.111504

19. Moro T, Tinsley G, Pacelli FQ, Marcolin G, Bianco A, Paoli A. Twelve Months of Time-restricted Eating and Resistance Training Improves Inflammatory Markers and Cardiometabolic Risk Factors. Med Sci Sports Exerc. 2021;53: 2577–2585. doi:10.1249/MSS.0000000000002738

20. Che T, Yan C, Tian D, Zhang X, Liu X, Wu Z. Time-restricted feeding improves blood glucose and insulin sensitivity in overweight patients with type 2 diabetes: a randomised controlled trial. Nutr Metab (Lond). 2021;18. doi:10.1186/s12986-021-00613-9

21. Christensen RAG, High S, Wharton S, Kamran E, Dehlehhosseinzadeh M, Fung M, et al. Sequential diets and weight loss: Including a low-carbohydrate high-fat diet with and without time-restricted feeding. Nutrition. 2021;91–92. doi:10.1016/j.nut.2021.111393

22. Isenmann E, Dissemond J, Geisler S. The effects of a macronutrient-based diet and time-restricted feeding (16:8) on body composition in physically active individuals—a 14-week randomised controlled trial. Nutrients. 2021;13. doi:10.3390/nu13093122

23. Park SJ, Yang JW, Song YJ. The Effect of Four Weeks Dietary Intervention with 8-Hour Time-Restricted Eating on Body Composition and Cardiometabolic Risk Factors in Young Adults. Nutrients. 2021;13. doi:10.3390/nu13072164 WE  - Science Citation Index Expanded (SCI-EXPANDED)

24. Prasad M, Fine K, Gee A, Nair N, Popp CJ, Cheng B, et al. A smartphone intervention to promote time restricted eating reduces body weight and blood pressure in adults with overweight and obesity: A pilot study. Nutrients. 2021;13. doi:10.3390/nu13072148

25. Peeke PM, Greenway FL, Billes SK, Zhang DC, Fujioka K. Effect of time restricted eating on body weight and fasting glucose in participants with obesity: results of a randomized, controlled, virtual clinical trial. Nutr Diabetes. 2021;11. doi:10.1038/s41387-021-00149-0 WE  - Science Citation Index Expanded (SCI-EXPANDED)

26. Lobene AJ, Panda S, Mashek DG, Manoogian ENC, Gallant KMH, Chow LS. Time-Restricted Eating for 12 Weeks Does Not Adversely Alter Bone Turnover in Overweight Adults. Nutrients. 2021;13. doi:10.3390/nu13041155 WE  - Science Citation Index Expanded (SCI-EXPANDED)

27. Ahmed N, Farooq J, Siddiqi HS, Meo SA, Kulsoom B, Laghari AH, et al. Impact of Intermittent Fasting on Lipid Profile-A Quasi-Randomized Clinical  Trial. Frontiers in nutrition. Switzerland; 2020. p. 596787. doi:10.3389/fnut.2020.596787

28. Ribeiro DE, Santiago AF, De Abreu WC. Continuous energy restriction (CER) plus 16/8 time-restricted feeding improve body composition and metabolic parameters in overweight and obese, but no more than CER alone. Nutr Healthy Aging. 2021;6: 147–156. doi:10.3233/NHA-200106

29. Kesztyüs D, Vorwieger E, Schönsteiner D, Gulich M, Kesztyüs T. Applicability of time-restricted eating for the prevention of lifestyle-dependent diseases in a working population: Results of a pilot study in a pre-post design . GMS German Medical Science. 2021;19. doi:10.3205/000291

30. Moro T, Tinsley G, Longo G, Grigoletto D, Bianco A, Ferraris C, et al. Time-restricted eating effects on performance, immune function, and body composition in elite cyclists: a randomized controlled trial. J Int Soc Sports Nutr. 2020;17. doi:10.1186/s12970-020-00396-z WE  - Science Citation Index Expanded (SCI-EXPANDED)

31. Lowe DA, Wu N, Rohdin-Bibby L, Moore AH, Kelly N, Liu YE, et al. Effects of Time-Restricted Eating on Weight Loss and Other Metabolic Parameters in Women and Men With Overweight and Obesity The TREAT Randomized Clinical Trial. JAMA Intern Med. 2020;180: 1491–1499. doi:10.1001/jamainternmed.2020.4153

32. Kunduraci YE, Ozbek H. Does the Energy Restriction Intermittent Fasting Diet Alleviate Metabolic Syndrome Biomarkers? A Randomized Controlled Trial. Nutrients. 2020;12. doi:10.3390/nu12103213 WE  - Science Citation Index Expanded (SCI-EXPANDED)

33. Jones R, Pabla P, Mallinson J, Nixon A, Taylor T, Bennett A, et al. Two weeks of early time-restricted feeding (eTRF) improves skeletal muscle insulin and anabolic sensitivity in healthy men. AMERICAN JOURNAL OF CLINICAL NUTRITION. 2020;112: 1015–1028. doi:10.1093/ajcn/nqaa192 WE  - Science Citation Index Expanded (SCI-EXPANDED)

34. Chow LS, Manoogian ENC, Alvear A, Fleischer JG, Thor H, Dietsche K, et al. Time-Restricted Eating Effects on Body Composition and Metabolic Measures in Humans who are Overweight: A Feasibility Study. OBESITY. 2020;28: 860–869. doi:10.1002/oby.22756 WE  - Science Citation Index Expanded (SCI-EXPANDED)

35. Anton SD, Lee SA, Donahoo WT, McLaren C, Manini T, Leeuwenburgh C, et al. The effects of time restricted feeding on overweight, older adults: A pilot study. Nutrients. 2019;11. doi:10.3390/nu11071500

36. Gabel K, Hoddy KK, Haggerty N, Song J, Kroeger CM, Trepanowski JF, et al. Effects of 8-hour time restricted feeding on body weight and metabolic disease risk factors in obese adults: A pilot study. Nutr Healthy Aging. 2018;4: 345–353. doi:10.3233/NHA-170036
