# Supplementary material for: Partially unraveling mechanistic underpinning and weight loss effects of time-restricted eating across diverse adult populations: A systematic review and meta-analyses of prospective studies
Source: PLoS One. 2025 Jan 15;20(1):e0314685. doi: 10.1371/journal.pone.0314685 (PMC11734929; doi:10.1371/journal.pone.0314685)
Supplement: S3 Table — (DOCX) [file pone.0314685.s004.docx]

**Supplementary S12.** The Alterations in Daily Caloric Intake among Adults Following the TRE.

| **Energy intake (kcal/day)** | **Chronic diseases** | | **Healthy adults** | **Overweight/Obesity** | | **Overall** | |
| --- | --- | --- | --- | --- | --- | --- | --- |
|  | **< -3 kg**  **(N = 5)** | **> = -3 kg**  **(N = 1)** | **< -3 kg**  **(N = 5)** | **<= -3 kg**  **(N = 3)** | **> = -3 kg**  **(N = 9)** | **< -3 kg**  **(N = 11)** | **> = -3 kg**  **(N = 10)** |
| **At baseline** | | | | | | | |
| Mean ( SD ) | 1740 ( 262 ) | 2540 ( NA) | 2140 ( 162 ) | 2130 ( 212 ) | 1880 (206) | 2010 ( 431 ) | 1970 ( 360 ) |
| **At the end of intervention** | | | | | | | |
| Mean ( SD ) | 1410 ( 89.2 ) | 2540 ( NA) | 2010 ( 567 ) | 2020 ( 206 ) | 1450 ( 187 ) | 1870 ( 474 ) | 1570 ( 414 ) |
| **Mean change** | | | | | | | |
| Mean ( SD ) | 325 ( 243 ) | 198 ( NA ) | 106 ( 163 ) | 111 ( 97.8 ) | 424 ( 210 ) | 167 ( 186 ) | 401 ( 210 ) |
| Abbreviations: SD, Standard deviation; N, number; kg, kilogram. | | | | | | | |
