# Supplementary material for: Partially unraveling mechanistic underpinning and weight loss effects of time-restricted eating across diverse adult populations: A systematic review and meta-analyses of prospective studies
Source: PLoS One. 2025 Jan 15;20(1):e0314685. doi: 10.1371/journal.pone.0314685 (PMC11734929; doi:10.1371/journal.pone.0314685)
Supplement: S4 Table — (DOCX) [file pone.0314685.s005.docx]

**Supplementary S14.** List of Articles Identified in the Literature Search with Reasons for Inclusion and Exclusion

| **No.** | **Titles** | **Reasons for Excluding and Including Articles** |
| --- | --- | --- |
| **A. Title & Abstract Screening** | | |
| 1 | Intermittent fasting: Describing engagement and associations with eating disorder behaviors and psychopathology among Canadian adolescents and young adults | Under-18 year old participants |
| 2 | A time-restricted feeding intervention in children and adolescents with obesity: The TRansForm study protocol | Under-18 year old participants |
| 3 | Continuous Glucose Monitoring in Adolescents With Obesity: Monitoring of Glucose Profiles, Glycemic Excursions, and Adherence to Time Restricted Eating Programs | Under-18 year old participants |
| 4 | Time-limited eating and continuous glucose monitoring in adolescents with obesity: A pilot study | Under-18 year old participants |
| 5 | Management of adolescent obesity What works and how we define success | Under-18 year old participants |
| 6 | Intermittent energy restriction is a feasible, effective, and acceptable intervention to treat adolescents with obesity | Under-18 year old participants |
| 7 | Intermittent fasting during Ramadan causes a transient increase in total, LDL, and HDL cholesterols and hs-CRP in ethnic obese adolescents | Under-18 year old participants |
| 8 | Weight reduction in adolescents | Under-18 year old participants |
| 9 | Comparative dietary effectiveness of a modified government-recommended diet with avoidance of ultra-processed foods on weight and metabolic management in children and adolescents: An open-label, randomized study | Under-18 year old participants |
| 10 | Calorically restricted diets decrease PCSK9 in overweight adolescents | Under-18 year old participants |
| 11 | A placebo-controlled pilot study of adjunctive olanzapine for adolescents with anorexia nervosa | Under-18 year old participants |
| 12 | Early vs. Late Time Restricted Eating in Adolescents With Obesity at Risk for Diabetes | Under-18 year old participants |
| 13 | Time Limited Eating in Adolescents With Type 2 Diabetes | Under-18 year old participants |
| 14 | Time Limited Eating in Adolescents With Diabetes (Time LEAD): a Pilot Study | Under-18 year old participants |
| 15 | Time-Restricted Feeding in Children and Adolescents With Obesity | Under-18 year old participants |
| 16 | Social media and the change in the eating behavior of adolescents | Under-18 year old participants |
| 17 | Caloric restriction as a possible pitfall for persistent acromegaly follow-up – case report | Case reports |
| 18 | Time restricted eating facilitates weight loss and improves cardiometabolic profile in a female veteran with multiple sclerosis: A case report | Case reports |
| 19 | Annular prurigo pigmentosa with sensory dysthesia with ketogenic diet: A case report | Case reports |
| 20 | Treating Diabetes Utilizing a Low Carbohydrate Ketogenic Diet and Intermittent Fasting Without Significant Weight Loss: A Case Report | Case reports |
| 21 | History of dietary treatment: Guelpa & Marie first report of intermittent fasting for epilepsy in 1911 | Case reports |
| 22 | Effect of intermittent vs. daily calorie restriction on changes in weight and patient-reported outcomes in people with multiple sclerosis | Case reports |
| 23 | Fluctuation of blood glucose levels in an infant with an ileostomy on continuous glucose monitoring: A case report | Case reports |
| 24 | The influence of intermittent fasting on the circadian pattern of melatonin while controlling for caloric intake, energy expenditure, light exposure, and sleep schedules: A preliminary report | Case reports |
| 25 | Comparison of the effect of modified intermittent fasting and daily calorie restriction on sleep quality, anthropometric data, and body composition in women with obesity or overweight: study protocol of a randomized controlled trial | Protocol |
| 26 | Comparison of weight loss induced by daily caloric restriction versus intermittent fasting (DRIFT) in individuals with obesity: study protocol for a 52-week randomized clinical trial | Protocol |
| 27 | Effect of time restricted feeding on anthropometric measures, eating behavior, stress, and brain-derived neurotrophic factor (BDNF) and lipopolysaccharide-binding protein (LBP) levels in women with overweight/obesity and food addiction: a study protocol for a randomized clinical trial | Protocol |
| 28 | Exploratory analysis of one versus two-day intermittent fasting protocols on the gut microbiome and plasma metabolome in adults with overweight/obesity | Protocol |
| 29 | Efficacy of time-restricted eating and behavioural economic interventions in reducing fasting plasma glucose, HbA1c and cardiometabolic risk factors compared with time-restricted eating alone or usual care in patients with impaired fasting glucose: Protocol for an open-label randomised controlled trial | Protocol |
| 30 | Time-restricted eating to improve cardiometabolic health: The New York time-restricted eating randomized clinical trial – Protocol overview | Protocol |
| 31 | Cardiometabolic and Anthropometric Outcomes of Intermittent Fasting Among Civil Servants With Overweight and Obesity: Study Protocol for a Nonrandomized Controlled Trial | Protocol |
| 32 | INTERmittent FASTing in people with insulin-treated type 2 diabetes mellitus – the INTERFAST-2 study protocol | Protocol |
| 33 | Study protocol for the Shifting Weight using Intermittent Fasting in night shift workers (SWIFt) study: A three-arm randomised controlled trial comparing three weight loss strategies in night shift workers with obesity | Protocol |
| 34 | Effect of intermittent fasting after ST-elevation myocardial infarction on left ventricular function: study protocol of a pilot randomised controlled trial (INTERFAST-MI) | Protocol |
| 35 | Effects of Early vs. Late Time-Restricted Eating on Cardiometabolic Health, Inflammation, and Sleep in Overweight and Obese Women: A Study Protocol for the ChronoFast Trial | Protocol |
| 36 | Protocol for a randomised controlled trial on the feasibility and effects of 10-hour time-restricted eating on cardiometabolic disease risk among career firefighters doing 24-hour shift work: The Healthy Heroes Study | Protocol |
| 37 | Intermittent versus continuous low-energy diet in patients with type 2 diabetes: Protocol for a pilot randomized controlled trial | Protocol |
| 38 | Isolated and combined effects of high-intensity interval training and time-restricted eating on glycaemic control in reproductive-aged women with overweight or obesity: Study protocol for a four-armed randomised controlled trial | Protocol |
| 39 | Imperial Satiety Protocol: A new non-surgical weight-loss programme, delivered in a health care setting, produces improved clinical outcomes for people with obesity | Protocol |
| 40 | Protocol for a single-centre, parallel-group, randomised, controlled, superiority trial on the effects of time-restricted eating on body weight, behaviour and metabolism in individuals at high risk of type 2 diabetes: The REStricted Eating Time (RESET) study | Protocol |
| 41 | Rationale and protocol for a randomized controlled trial comparing daily calorie restriction versus intermittent fasting to improve glycaemia in individuals at increased risk of developing type 2 diabetes | Protocol |
| 42 | A delayed morning and earlier evening time-restricted feeding protocol for improving glycemic control and dietary adherence in men with overweight/obesity: A randomized controlled trial | Protocol |
| 43 | Intermittent Fasting (Alternate Day Fasting) in Healthy, Non-obese Adults: Protocol for a Cohort Trial with an Embedded Randomized Controlled Pilot Trial | Protocol |
| 44 | Intermittent fasting, energy balance and associated health outcomes in adults: Study protocol for a randomised controlled trial | Protocol |
| 45 | Determining how best to support overweight adults to adhere to lifestyle change: Protocol for the SWIFT study | Protocol |
| 46 | Changes in NPY and POMC, but not serotonin transporter, following a restricted feeding/repletion protocol in rats | Protocol |
| 47 | Mediterranean versus vegetarian diet for cardiovascular disease prevention (the CARDIVEG study): study protocol for a randomized controlled trial | Protocol |
| 48 | Time-restricted eating to improve cardiometabolic health: the New York time-restricted eating randomized clinical trial ‚Äì Protocol overview | Protocol |
| 49 | Early Time-Restricted Eating for Weight Loss and Metabolic Health: a Secondary Per-Protocol Analysis | Protocol |
| 50 | Monitoring During Different Intermittent Fasting Protocols in Non-Obese Adults | Protocol |
| 51 | Intermittent fasting & performance: the iFast clinical trial protocol | Protocol |
| 52 | Phase angle is associated with muscle health and cardiorespiratory fitness in older breast cancer survivors | Animals |
| 53 | Possible homeostatic, glucose uptake mechanisms and hepato-pancreatic histological effects of intermittent fasting, exercise, starvation, and honey in streptozotocin-induced diabetes in rats | Animals |
| 54 | Pu-erh tea intake enhances the anti-obesity effect of intermittent fasting via modulating follicle-stimulating hormone and gut dysbacteriosis in female high-fat-diet mice | Animals |
| 55 | A long-term obesogenic high-fat diet in mice partially dampens the anti-frailty benefits of late-life intermittent fasting | Animals |
| 56 | Effects of Intermittent Fasting on Hypothalamus–Pituitary–Thyroid Axis, Palatable Food Intake, and Body Weight in Stressed Rats | Animals |
| 57 | Obesity and Overweight: Probing Causes, Consequences, and Novel Therapeutic Approaches Through the American Heart Association’s Strategically Focused Research Network | Animals |
| 58 | Nutritional and supplementation strategies of Spanish natural elite bodybuilders in precontest | Animals |
| 59 | Depot-Dependent Impact of Time-Restricted Feeding on Adipose Tissue Metabolism in High Fat Diet-Induced Obese Male Mice | Animals |
| 60 | Time-restricted feeding ameliorates dextran sulfate sodium-induced colitis via reducing intestinal inflammation | Animals |
| 61 | Time-restricted feeding improves metabolic and endocrine profiles in mice with polycystic ovary syndrome | Animals |
| 62 | High-Fat-Diet-Evoked Disruption of the Rat Dorsomedial Hypothalamic Clock Can Be Prevented by Restricted Nighttime Feeding | Animals |
| 63 | Caffeine suppresses high-fat diet-induced body weight gain in mice depending on feeding timing | Animals |
| 64 | Alternate-day fasting prevents non-alcoholic fatty liver disease and working memory impairment in diet-induced obese mice | Animals |
| 65 | Intermittent fasting attenuates lipopolysaccharide-induced acute lung injury in mice by modulating macrophage polarization | Animals |
| 66 | Time-restricted feeding rescues circadian disruption-aggravated progression of Alzheimer's disease in diabetic mice | Animals |
| 67 | Chronic activation of cardiac Atg-5 and pancreatic Atg-7 by intermittent fasting alleviates acute myocardial infarction in old rats | Animals |
| 68 | Effect of intermittent fasting and complete fasting on biochemical and histological parameters in high fat high sugar induced rat model of obesity | Animals |
| 69 | Intermittent Fasting Is Associated With a Decreased Risk of Age-Related Macular Degeneration | Animals |
| 70 | A qualitative exploration of facilitators and barriers of adherence to time-restricted eating | Animals |
| 71 | Time restricted feeding decreases renal innate immune cells and blood pressure in hypertensive mice | Animals |
| 72 | Alternate-day fasting, a high-sucrose/caloric diet and praziquantel treatment influence biochemical and behavioral parameters during Schistosoma mansoni infection in male BALB/c mice | Animals |
| 73 | Intermittent fasting activates markers of autophagy in mouse liver, but not muscle from mouse or humans | Animals |
| 74 | AMPK-PPARγ-Cidec Axis Drives the Fasting-Induced Lipid Droplet Aggregation in the Liver of Obese Mice | Animals |
| 75 | Sex-specific differences in metabolic outcomes after sleeve gastrectomy and intermittent fasting in obese middle-aged mice | Animals |
| 76 | Intermittent fasting, high-intensity interval training, or a combination of both have beneficial effects in obese mice with nonalcoholic fatty liver disease | Animals |
| 77 | Alternate Day Fasting Improves Endothelial Function in Type 2 Diabetic Mice: Role of Adipose-Derived Hormones | Animals |
| 78 | Time-restricted feeding prevents metabolic diseases through the regulation of galanin/GALR1 expression in the hypothalamus of mice | Animals |
| 79 | Adipocyte-derived PGE2 is required for intermittent fasting-induced Treg proliferation and improvement of insulin sensitivity | Animals |
| 80 | 2D DIGE proteomic analysis reveals fasting-induced protein remodeling through organ-specific transcription factor(s) in mice | Animals |
| 81 | Time-Restricted Feeding Restored Insulin-Growth Hormone Balance and Improved Substrate and Energy Metabolism in MC4RKO Obese Mice | Animals |
| 82 | Intermittent fasting with a high-protein diet mitigated osteoarthritis symptoms by increasing lean body mass and reducing inflammation in osteoarthritic rats with Alzheimer's disease-like dementia | Animals |
| 83 | Histological Effects of Intermittent Fasting Concomitant with Levothyroxine Administration on the Thyroid Gland Structure of Adult Male Albino Rats | Animals |
| 84 | Alternate-Day High Fat-Normal Chow Diet Ameliorates HFD-Induced Obesity and Restores Intestinal Immunity | Animals |
| 85 | Impact of intermittent fasting on laboratory, radiological, and anthropometric parameters in NAFLD patients | Animals |
| 86 | The Effect of Calorie Restriction and Intermittent Fasting on Impaired Cognitive Function in High-Fat Diet-Induced Obesity Started Post-Weaning in Male Wistar Rat | Animals |
| 87 | The rationale and design of a Mediterranean diet accompanied by time restricted feeding to optimise the management of type 2 diabetes: The MedDietFast randomised controlled trial | Animals |
| 88 | Differential effects of time-restricted feeding on circadian locomotor activity, food intake and body weight gain in BALB/cJ and C57BL/6J mice | Animals |
| 89 | Delivery method matters: omega-3 supplementation by restricted feeding period and oral gavage has a distinct impact on corticosterone secretion and anxious behavior in adolescent rats | Animals |
| 90 | A low-glucose eating pattern improves biomarkers of postmenopausal breast cancer risk: An exploratory secondary analysis of a randomized feasibility trial | Animals |
| 91 | Intermittent Fasting Attenuates High-Fat Diet-Induced Cerebellar Changes in Rats: Involvement of TNF-α, Autophagy, and Oxidative Stress | Animals |
| 92 | Intermittent Fasting Reshapes the Gut Microbiota and Metabolome and Reduces Weight Gain More Effectively Than Melatonin in Mice | Animals |
| 93 | Time-restricted feeding restores obesity-induced alteration in adipose tissue immune cell phenotype | Animals |
| 94 | The ketone body β-hydroxybutyrate mitigates the senescence response of glomerular podocytes to diabetic insults | Animals |
| 95 | Distinct Metabolic States Are Observed in Hypoglycemia Induced in Mice by Ricin Toxin or by Fasting | Animals |
| 96 | Time-restricted feeding during puberty ameliorates adiposity and prevents hepatic steatosis in a mouse model of childhood obesity | Animals |
| 97 | Does intermittent fasting associated with aerobic training influence parameters related to the gut-brain axis of Wistar rats? | Animals |
| 98 | Fibroblast growth factor 21 as a potential biomarker for improved locomotion and olfaction detection ability after weight reduction in obese mice | Animals |
| 99 | Independent of Calorie Intake, Short-term Alternate-day Fasting Alleviates NASH, With Modulation of Markers of Lipogenesis, Autophagy, Apoptosis, and Inflammation in Rats | Animals |
| 100 | Fasting and refeeding cycles alter subcutaneous white depot growth dynamics and the morphology of brown adipose tissue in female rats | Animals |
| 101 | Effects of Daytime Dry Fasting on Hydration, Glucose Metabolism and Circadian Phase: A Prospective Exploratory Cohort Study in Bahá'í Volunteers | Animals |
| 102 | Effect of intermittent and continuous caloric restriction on Sirtuin1 concentration depends on sex and body mass index | Animals |
| 103 | Maternal intermittent fasting during pregnancy induces fetal growth restriction and down-regulated placental system A amino acid transport in the rat | Animals |
| 104 | Time-restricted feeding drives periods of rapid food consumption in rats fed a high-fat diet with liquid sucrose | Animals |
| 105 | Intermittent fasting compromises the performance of eutrophic rats submitted to resistance training | Animals |
| 106 | Effects of feeding time on markers of muscle metabolic flexibility following acute aerobic exercise in trained mice undergoing time restricted feeding | Animals |
| 107 | Intermittent Fasting: Clinical Considerations | Animals |
| 108 | Dynamic observation of the progression of chronic gastritis to gastric cancer in a disease–TCM pattern rat model | Animals |
| 109 | Effects of christian orthodox fasting versus time‐restricted eating on plasma irisin concentrations among overweight metabolically healthy individuals | Animals |
| 110 | Intermittent Fasting Alleviates Cognitive Impairments and Hippocampal Neuronal Loss but Enhances Astrocytosis in Mice with Subcortical Vascular Dementia | Animals |
| 111 | An exploratory investigation of the impact of ‘fast’ and ‘feed’ days during intermittent energy restriction on free-living energy balance behaviours and subjective states in women with overweight/obesity | Animals |
| 112 | Health Effects of Alternate Day Fasting Versus Pair-Fed Caloric Restriction in Diet-Induced Obese C57Bl/6J Male Mice | Animals |
| 113 | Intermittent fasting, nutritional supplementation, and resistance training as strategy for body weight loss: An update [Ayuno intermitente, suplementación nutricional y entrenamiento de resistencia como estrategia para la pérdida de peso corporal: Una actualización] | Animals |
| 114 | Time-restricted feeding mice a high-fat diet induces a unique lipidomic profile | Animals |
| 115 | Changes in subjective measures of appetite during 6 months of alternate day fasting with a low carbohydrate diet | Animals |
| 116 | The effects of calorie restriction and time-restricted feeding on igf1 serum level and lipid profile in male wister rats with previous obesity | Animals |
| 117 | Chronic stress accelerates the process of gastric precancerous lesions in rats | Animals |
| 118 | Time-restricted feeding in dark phase of circadian cycle and/or westernized diet cause mixed hyperlipidemia in rats [La alimentación con restricción de tiempo en la fase oscura del ciclo circadiano y/o la dieta occidentalizada causan hiperlipidemia mixta en las ratas] | Animals |
| 119 | Mice held at an environmental photic cycle oscillating at their tau-like period length do not show the high-fat diet-induced obesity that develops under the 24-hour photic cycle | Animals |
| 120 | Alternate day fasting combined with a low carbohydrate diet: Effect on sleep quality, duration, insomnia severity and risk of obstructive sleep apnea in adults with obesity | Animals |
| 121 | Metabolism and Metabolic Disorders and the Microbiome: The Intestinal Microbiota Associated With Obesity, Lipid Metabolism, and Metabolic Health—Pathophysiology and Therapeutic Strategies | Animals |
| 122 | Early or delayed time-restricted feeding prevents metabolic impact of obesity in mice | Animals |
| 123 | Intermittent fasting ameliorated high-fat diet-induced memory impairment in rats via reducing oxidative stress and glial fibrillary acidic protein expression in brain | Animals |
| 124 | Intermittent fasting improves lipid metabolism through changes in gut microbiota in diet-induced obese mice | Animals |
| 125 | Time restricted feeding provides a viable alternative to alternate day fasting when evaluated in terms of redox homeostasis in rats | Animals |
| 126 | Therapeutic time-restricted feeding reduces renal tumor bioluminescence in mice but fails to improve anti-CTLA-4 efficacy | Animals |
| 127 | Intermittent Fasting Attenuates Apoptosis, Modulates Autophagy and Preserves Telocytes in Doxorubicin Induced Cardiotoxicity in Albino Rats: A Histological Study | Animals |
| 128 | Ketone production by ketogenic diet and by intermittent fasting has different effects on the gut microbiota and disease progression in an Alzheimer’s disease rat model | Animals |
| 129 | Intermittent fasting, adipokines, insulin sensitivity, and hypothalamic neuropeptides in a dietary overload with high-fat or high-fructose diet in mice | Animals |
| 130 | Time-restricted feeding improves body weight gain, lipid profiles, and atherogenic indices in cafeteria-diet-fed rats: Role of browning of inguinal white adipose tissue | Animals |
| 131 | Intermittent fasting and high-intensity exercise elicit sexual-dimorphic and tissue-specific adaptations in diet-induced obese mice | Animals |
| 132 | The Effects of Age and Fasting Models on Blood Pressure, Insulin/Glucose Profile, and Expression of Longevity Proteins in Male Rats | Animals |
| 133 | Time-restricted feeding ameliorates maternal high-fat diet-induced fetal lung injury | Animals |
| 134 | Long-term feeding of a high-fat diet ameliorated age-related phenotypes in SAMP8 mice | Animals |
| 135 | Intermittent fasting for twelve weeks leads to increases in fat mass and hyperinsulinemia in young female wistar rats | Animals |
| 136 | Intermittent fasting benefits on alpha- and beta-cell arrangement in diet-induced obese mice pancreatic islet | Animals |
| 137 | Dietary weight loss strategies for self and patients: A cross-sectional survey of female physicians | Animals |
| 138 | Time of feeding alters obesity-associated parameters and gut bacterial communities, but not fungal populations, in C57BL/6 male mice | Animals |
| 139 | Central administration of REV-ERBα agonist promotes opposite responses on energy balance in fasted and fed states | Animals |
| 140 | Alternate day fasting combined with a low-carbohydrate diet for weight loss, weight maintenance, and metabolic disease risk reduction | Animals |
| 141 | Estradiol regulates daily rhythms underlying diet-induced obesity in female mice | Animals |
| 142 | Diurnal influences of fasted and non-fasted brisk walking on gastric emptying rate, metabolic responses, and appetite in healthy males | Animals |
| 143 | Intermittent fasting improves metabolic flexibility in short-term high-fat dietfed mice | Animals |
| 144 | Intermittent fasting increases energy expenditure and promotes adipose tissue browning in mice | Animals |
| 145 | Alternate-day feeding leads to improved glucose regulation on fasting days without significant weight loss in genetically obese mice | Animals |
| 146 | The effect of intermittent fasting diet on the hippocampus of adult male mouse after inducing demyelination by ethidium bromide injection [Efecto de la dieta de ayuno intermitente en el hipocampo de ratón macho adulto después de inducir la desmielinización por inyección de bromuro de etidio] | Animals |
| 147 | Comparison of glycemic improvement between intermittent calorie restriction and continuous calorie restriction in diabetic mice | Animals |
| 148 | Time-Restricted Feeding Improves Glucose Tolerance in Rats, but Only When in Line With the Circadian Timing System | Animals |
| 149 | Pancreatic adipocytes mediate hypersecretion of insulin in diabetes-susceptible mice | Animals |
| 150 | The role of time of food intake on upcoming liver disease in male Wistar rat | Animals |
| 151 | Time-restricted feeding attenuates high-fat diet-enhanced spontaneous metastasis of lewis lung carcinoma in mice | Animals |
| 152 | Intermittent fasting increases the expressions of SODs and catalase in granule and polymorphic cells and enhances neuroblast dendrite complexity and maturation in the adult gerbil dentate gyrus | Animals |
| 153 | Intermittent fasting exerts beneficial metabolic effects on blood pressure and cardiac structure by modulating local renin-angiotensin system in the heart of mice fed high-fat or high-fructose diets | Animals |
| 154 | Time-restricted feeding causes irreversible metabolic disorders and gut microbiota shift in pediatric mice | Animals |
| 155 | A comparison of dietary and caloric restriction models on body composition, physical performance, and metabolic health in young mice | Animals |
| 156 | Melanocortin-3 Receptors Expressed on Agouti-Related Peptide Neurons Inhibit Feeding Behavior in Female Mice | Animals |
| 157 | Intermittent administration of a fasting-mimicking diet intervenes in diabetes progression, restores β cells and reconstructs gut microbiota in mice | Animals |
| 158 | Short-term time-restricted feeding during the resting phase is sufficient to induce leptin resistance that contributes to development of obesity and metabolic disorders in mice | Animals |
| 159 | Time-restricted feeding mitigates high-fat diet–enhanced mammary tumorigenesis in MMTV-PyMT mice | Animals |
| 160 | Restructuring of the gut microbiome by intermittent fasting prevents retinopathy and prolongs survival in db/db mice | Animals |
| 161 | Different ketogenesis strategies lead to disparate seizure outcomes | Animals |
| 162 | Restricted feeding for 9 h in the active period partially abrogates the detrimental metabolic effects of a Western diet with liquid sugar consumption in mice | Animals |
| 163 | Intermittent food restriction in female rats induces SREBP high expression in hypothalamus and immediately postfasting hyperphagia | Animals |
| 164 | Intermittent fasting with or without exercise prevents weight gain and improves lipids in diet-induced obese mice | Animals |
| 165 | Eating behavior traits of successful weight losers during 12 months of alternate-day fasting: An exploratory analysis of a randomized controlled trial | Animals |
| 166 | A pilot feasibility study exploring the effects of a moderate time-restricted feeding intervention on energy intake, adiposity and metabolic physiology in free-living human subjects | Animals |
| 167 | Time-restricted feeding of a high-fat diet in male C57BL/6 mice reduces adiposity but does not protect against increased systemic inflammation | Animals |
| 168 | Structural changes to immune organs in rats after intermittent fasting following a high carb and fat diet | Animals |
| 169 | Differential effects of diet composition and timing of feeding behavior on rat brown adipose tissue and skeletal muscle peripheral clocks | Animals |
| 170 | Effects of caloric restriction on peroxisome proliferator-activated receptors and positive transcription elongation factor b expression in obese rats | Animals |
| 171 | Effects of A One-week Fasting Therapy in Patients with Type-2 Diabetes Mellitus and Metabolic Syndrome - A Randomized Controlled Explorative Study | Animals |
| 172 | Long-Term Energy Deficit in Mice Causes Long-Lasting Hypothalamic Alterations after Recovery | Animals |
| 173 | Intermittent fasting pretreatment prevents cognitive impairment in a rat model of chronic cerebral hypoperfusion | Animals |
| 174 | Nutritional adequacy of diets for adolescents with overweight and obesity: Considerations for dietetic practice | Animals |
| 175 | Intermittent fasting reduces body fat but exacerbates hepatic insulin resistance in young rats regardless of high protein and fat diets | Animals |
| 176 | Effect of alternate day fasting on markers of bone metabolism: An exploratory analysis of a 6-month randomized controlled trial | Animals |
| 177 | Prepuberal light phase feeding induces neuroendocrine alterations in adult rats | Animals |
| 178 | Time-restricted feeding improves insulin resistance and hepatic steatosis in a mouse model of postmenopausal obesity | Animals |
| 179 | The effects of intermittent calorie restriction on metabolic health: Rationale and study design of the HELENA Trial | Animals |
| 180 | Alternate-day fasting protects the livers of mice against high-fat diet-induced inflammation associated with the suppression of Toll-like receptor 4/nuclear factor κB signaling | Animals |
| 181 | Gender differences in response to prolonged every-other-day feeding on the proliferation and apoptosis of hepatocytes in mice | Animals |
| 182 | Intermittent fasting favored the resolution of Salmonella typhimurium infection in middle-aged BALB/c mice | Animals |
| 183 | Time-restricted feeding reduces adiposity in mice fed a high-fat diet | Animals |
| 184 | Altered appetite-mediating hormone concentrations precede compensatory overeating after severe, short-term energy deprivation in healthy adults | Animals |
| 185 | Middle age onset short-term intermittent fasting dietary restriction prevents brain function impairments in male Wistar rats | Animals |
| 186 | Chronic high fat diet induces cardiac hypertrophy and fibrosis in mice | Animals |
| 187 | Meal feeding improves oral glucose tolerance in male rats and causes adaptations in postprandial islet hormone secretion that are independent of plasma incretins or glycemia | Animals |
| 188 | Food restriction by intermittent fasting induces diabetes and obesity and aggravates spontaneous atherosclerosis development in hypercholesterolaemic mice | Animals |
| 189 | Xiaochaihu Decoction attenuates the vicious circle between the oxidative stress and the ALP inactivation through LPS-catecholamines interactions in gut, liver and brain during CCI4+ethanol-induced mouse HCC | Animals |
| 190 | Intermittent fasting modulation of the diabetic syndrome in streptozotocin-injected rats | Animals |
| 191 | Assessment of enhanced endothelium - dependent vasodilation by intermittent fasting in Wistar albino rats | Animals |
| 192 | Intermittent fasting in mice does not improve hindlimb motor performance after spinal cord injury | Animals |
| 193 | Effect of intermittent fasting on prostate cancer tumor growth in a mouse model | Animals |
| 194 | Chronic alternate-day fasting results in reduced diastolic compliance and diminished systolic reserve in rats | Animals |
| 195 | Intermittent administration of a leucine-deprived diet is able to intervene in type 2 diabetes in db/db mice | Animals |
| 196 | Adult-onset calorie restriction and fasting delay spontaneous tumorigenesis in p53-deficient mice | Animals |
| 197 | The effects of daily fasting hours on shaping gut microbiota in mice | Animals |
| 198 | Effects of Fasting and Phoenix dactylifera on the Expression of Major Drug-Metabolizing Enzymes in the Mouse Livers | Animals |
| 199 | Physiological and behavioral responses to intermittent starvation in C57BL/6J mice | Animals |
| 200 | Effects of modified alternate-day fasting regimens on adipocyte size, triglyceride metabolism, and plasma adiponectin levels in mice | Animals |
| 201 | Circadian clock network desynchrony promotes weight gain and alters glucose homeostasis in mice | Animals |
| 202 | Effectiveness of Ramadan diurnal intermittent fasting and concurrent training in the management of obesity: is the combination worth the weight? | Religious fasting |
| 203 | Ramadan diurnal intermittent fasting is associated with significant plasma metabolomics changes in subjects with overweight and obesity: A prospective cohort study | Religious fasting |
| 204 | Nigerian Muslim’s Perceptions of Changes in Diet, Weight, and Health Status during Ramadan: A Nationwide Cross-Sectional Study | Religious fasting |
| 205 | The effect of Ramadan fasting on nasal mucociliary activity and peak nasal inspiratory flow | Religious fasting |
| 206 | Changes in dietary intake, chronotype and sleep pattern upon Ramadan among healthy adults in Jeddah, Saudi Arabia: A prospective study | Religious fasting |
| 207 | Is Ramadan fasting associated with low scores of Healthy Eating Index? [¿Está asociado el ayuno de Ramadán con puntuaciones bajas en el Índice de Alimentación Saludable?] | Religious fasting |
| 208 | Haptoglobin polymorphism modulates cardiometabolic impacts of four consecutive weeks, dawn to sunset Ramadan intermittent fasting among subjects with overweight/obesity | Religious fasting |
| 209 | Seven decades of Ramadan intermittent fasting research: Bibliometrics analysis, global trends, and future directions | Religious fasting |
| 210 | Exergaming During Ramadan Intermittent Fasting Improve Body Composition as Well as Physiological and Psychological Responses to Physical Exercise in Adolescents With Obesity | Religious fasting |
| 211 | Impact of Ramadan intermittent fasting on metabolic and inflammatory profiles in type 2 diabetic patients | Religious fasting |
| 212 | Ramadan Intermittent Fasting Is Associated with Changes in Circulating Proprotein Convertase Subtilisin/Kexin Type 9 (PCSK9) in Metabolically Healthy Obese Subjects | Religious fasting |
| 213 | Ramadan Diurnal Intermittent Fasting Is Associated With Attenuated FTO Gene Expression in Subjects With Overweight and Obesity: A Prospective Cohort Study | Religious fasting |
| 214 | Effect of intermittent fasting and chronotherapy on blood pressure control in hypertensive patients during Ramadan | Religious fasting |
| 215 | Effects of Diurnal Ramadan Intermittent Fasting on Cardiometabolic Risk Factors and Sleep Quality in Healthy Turkish Adults | Religious fasting |
| 216 | Clinical implications of intermittent Ramadan fasting on stable plaque psoriasis: A prospective observational study | Religious fasting |
| 217 | Ramadan fasting improves body composition without exacerbating depression in males with diagnosed major depressive disorders | Religious fasting |
| 218 | Remodeling of the gut microbiome during Ramadan-associated intermittent fasting | Religious fasting |
| 219 | A rare instance of Wilkie's syndrome in a young male during the holy month of Ramadan | Religious fasting |
| 220 | Intermittent Fasting During Ramadan Improves Insulin Sensitivity and Anthropometric Parameters in Healthy Young Muslim Men | Religious fasting |
| 221 | Impact of ramadan intermittent fasting on the heart rate variability and cardiovascular parameters of patients with controlled hypertension | Religious fasting |
| 222 | Effects of Ramadan intermittent fasting and pattern of nutrients intake on BMI and MUAC of a population consisting of Indian Muslims | Religious fasting |
| 223 | Effects of time-restricted feeding during ramadan on dietary intake, body composition and metabolic outcomes | Religious fasting |
| 224 | Effects of ramadan intermittent fasting on gut hormones and body composition in males with obesity | Religious fasting |
| 225 | Study of Beneficial Impact on Specific Biomarkers in Type 2 Diabetes During Ramadan Fasting (Unintentional Intermittent Fasting) | Religious fasting |
| 226 | Impact of Ramadan on Physical Activity and Sleeping Patterns in Individuals with Type 2 Diabetes: The First Study Using Fitbit Device | Religious fasting |
| 227 | Ramadan diurnal intermittent fasting modulates SOD2, TFAM, Nrf2, and sirtuins (SIRT1, SIRT3) gene expressions in subjects with overweight and obesity | Religious fasting |
| 228 | The safety and impact of a model of intermittent, time-restricted circadian fasting (“ramadan fasting”) on hidradenitis suppurativa: Insights from a multicenter, observational, cross-over, pilot, exploratory study | Religious fasting |
| 229 | Intermittent Fasting during Ramadan and Its Effects in Individuals with Metabolic Syndrome | Religious fasting |
| 230 | Effect of Ramadan diurnal fasting on visceral adiposity and serum adipokines in overweight and obese individuals | Religious fasting |
| 231 | The effects of Ramadan intermittent fasting on the underlying mechanisms of force production capacity during maximal isometric voluntary contraction | Religious fasting |
| 232 | The impact of intermittent fasting (Ramadan fasting) on psoriatic arthritis disease activity, enthesitis, and dactylitis: A multicentre study | Religious fasting |
| 233 | Effects of intermittent fasting during ramadan on insulin-like growth factor-1, interleukin 2, and lipid profile in healthy muslims | Religious fasting |
| 234 | Ramadan model of intermittent fasting for 28 d had no major effect on body composition, glucose metabolism, or cognitive functions in healthy lean men | Religious fasting |
| 235 | The effect of Ramadan intermittent fasting on lipid peroxidation in healthy young men while controlling for diet and sleep: A pilot study | Religious fasting |
| 236 | Effects of Ramadan fasting on glucose homeostasis and adiponectin levels in healthy adult males | Religious fasting |
| 237 | A Prospective Study of the Physiological and Neurobehavioral Effects of Ramadan Fasting in Preteen and Teenage Boys | Religious fasting |
| 238 | EFFECTS OF RAMADAN FASTING ON BLOOD PRESSURE IN NORMOTENSIVE MALES | Religious fasting |
| 239 | Critical analysis of the published literature about the effects of Ramadan intermittent fasting on healthy children’s physical capacities | Religious fasting |
| 240 | The effect of fasting on erectile function and sexual desire on men in the month of ramadan | Religious fasting |
| 241 | The effect of Ramadan intermittent fasting on dynamic postural control in judo athletes | Religious fasting |
| 242 | Effect of Ramadan intermittent fasting on body composition and neuromuscular performance in young athletes: A pilot study | Religious fasting |
| 243 | Effect of fasting in Ramadan on body composition and nutritional intake: A prospective study | Religious fasting |
| 244 | Effects of Ramadan intermittent fasting on postural control in judo athletes | Religious fasting |
| 245 | Impact of ramadan intermittent fasting on oxidative stress measured by urinary 15- F 2t -isoprostane | Religious fasting |
| 246 | Intermittent fasting during Ramadan attenuates proinflammatory cytokines and immune cells in healthy subjects | Religious fasting |
| 247 | Effect of Ramadan intermittent fasting on aerobic and anaerobic performance and perception of fatigue in male elite judo athletes. | Religious fasting |
| 248 | Effects of Ramadan intermittent fasting on inflammatory and biochemical biomarkers in males with obesity | Religious fasting |
| 249 | Ramadan intermittent fasting and physical training in management of obesity and overweight | Religious fasting |
| 250 | Intermittent fasting during Ramadan: does it affect sleep? | Religious fasting |
| 251 | Structural changes in gut microbiome after Ramadan fasting: a pilot study | Religious fasting |
| 252 | Effect of Ramadan intermittent fasting on inflammatory markers, disease severity, depression, and quality of life in patients with inflammatory bowel diseases: A prospective cohort study | Religious fasting |
| 253 | Metagenomic analysis of distal gut microbiota of the obesity during Intermittent fasting | Gut microbiome |
| 254 | Intermittent fasting promotes repair of rotator cuff injury in the early postoperative period by regulating the gut microbiota | Gut microbiome |
| 255 | Intermittent Fasting Promotes White Adipose Browning and Decreases Obesity by Shaping the Gut Microbiota | Gut microbiome |
| 256 | Effects of an Intermittent Fasting 5:2 Plus Program on Body Weight in Chinese Adults with Overweight or Obesity: A Pilot Study | The 5:2 diet |
| 257 | Effects of the 5:2 intermittent fasting diet on non-alcoholic fatty liver disease: A randomized controlled trial | The 5:2 diet |
| 258 | 5:2 intermittent fasting tapers food intake in the refeeding state and ameliorates metabolic disturbances in mice exposed to olanzapine | The 5:2 diet |
| 259 | Effect of four weeks of 5:2 intermittent fasting on energy intake and body mass index among obese male students aged 18-25 | The 5:2 diet |
| 260 | Intermittent fasting 5:2 diet: What is the macronutrient and micronutrient intake and composition? | The 5:2 diet |
| 261 | Effect of intermittent fasting 5:2 on body composition and nutritional intake among employees with obesity in Jakarta: a randomized clinical trial | The 5:2 diet |
| 262 | Effects of intermittent (5:2) or continuous energy restriction on basal and postprandial metabolism: a randomised study in normal-weight, young participants | The 5:2 diet |
| 263 | Impact of intermittent fasting (5:2) on ketone body production in healthy female subjects | The 5:2 diet |
| 264 | The Quest for Welfare-Friendly Feeding of Broiler Breeders: Effects of Daily vs. 5:2 Feed Restriction Schedules | The 5:2 diet |
| 265 | The Effects of Isocaloric Intermittent Fasting vs Daily Caloric Restriction on Weight Loss and Metabolic Risk Factors for Noncommunicable Chronic Diseases: A Systematic Review of Randomized Controlled or Comparative Trials | Meta-analysis/Literature review/Umbrella review/ Scoping Review |
| 266 | User Engagement and Weight Loss Facilitated by a Mobile App: Retrospective Review of Medical Records | Meta-analysis/Literature review/Umbrella review/ Scoping Review |
| 267 | Effects of dietary restriction on cognitive function: a systematic review and meta-analysis | Meta-analysis/Literature review/Umbrella review/ Scoping Review |
| 268 | Metabolic Efficacy of Time-Restricted Eating in Adults: A Systematic Review and Meta-Analysis of Randomized Controlled Trials | Meta-analysis/Literature review/Umbrella review/ Scoping Review |
| 269 | JPEN Journal Club 69. Umbrella reviews | Meta-analysis/Literature review/Umbrella review/ Scoping Review |
| 270 | The Effect of Early Time-Restricted Feeding on Glycemic Profile in Adults: A Systematic Review of Interventional Studies | Meta-analysis/Literature review/Umbrella review/ Scoping Review |
| 271 | Effects of intermittent fasting on cardiometabolic risk factors in patients with metabolic syndrome: A systematic review and meta-analysis of randomized controlled trials | Meta-analysis/Literature review/Umbrella review/ Scoping Review |
| 272 | Intermittent Fasting and Obesity-Related Health Outcomes: An Umbrella Review of Meta-analyses of Randomized Clinical Trials | Meta-analysis/Literature review/Umbrella review/ Scoping Review |
| 273 | The effects of Ramadan intermittent fasting on liver function in healthy adults: A systematic review, meta-analysis, and meta-regression | Meta-analysis/Literature review/Umbrella review/ Scoping Review |
| 274 | Effect of early time-restricted feeding on the metabolic profile of adults with excess weight: A systematic review with meta-analysis | Meta-analysis/Literature review/Umbrella review/ Scoping Review |
| 275 | Impact of Ramadan Diurnal Intermittent Fasting on Hypoglycemic Events in Patients With Type 2 Diabetes: A Systematic Review of Randomized Controlled Trials and Observational Studies | Meta-analysis/Literature review/Umbrella review/ Scoping Review |
| 276 | Metabolic Impact of Intermittent Fasting in Patients with Type 2 Diabetes Mellitus: A Systematic Review and Meta-analysis of Interventional Studies | Meta-analysis/Literature review/Umbrella review/ Scoping Review |
| 277 | AGA Clinical Practice Update on Lifestyle Modification Using Diet and Exercise to Achieve Weight Loss in the Management of Nonalcoholic Fatty Liver Disease: Expert Review | Meta-analysis/Literature review/Umbrella review/ Scoping Review |
| 278 | Food Timing, Circadian Rhythm and Chrononutrition: A Systematic Review of Time-Restricted Eating's Effects on Human Health | Meta-analysis/Literature review/Umbrella review/ Scoping Review |
| 279 | Time-restricted feeding and potential for type 2 diabetes mellitus: A narrative review | Meta-analysis/Literature review/Umbrella review/ Scoping Review |
| 280 | Beneficial effects of the ketogenic diet on nonalcoholic fatty liver disease: A comprehensive review of the literature | Meta-analysis/Literature review/Umbrella review/ Scoping Review |
| 281 | The Relationship Between Feasting Periods and Weight Gain: a Systematic Scoping Review | Meta-analysis/Literature review/Umbrella review/ Scoping Review |
| 282 | The effectiveness of intermittent fasting to reduce body mass index and glucose metabolism: A systematic review and meta-analysis | Meta-analysis/Literature review/Umbrella review/ Scoping Review |
| 283 | Dietary patterns and management of type 2 diabetes: A systematic review of randomised clinical trials | Meta-analysis/Literature review/Umbrella review/ Scoping Review |
| 284 | Comparison of time-restricted feeding and islamic fasting: A scoping review [Comparaison entre la prise alimentaire limitée dans le temps et le jeûne islamique: étude de portée] | Meta-analysis/Literature review/Umbrella review/ Scoping Review |
| 285 | Food intake rhythm and its implication on obesity, and related comorbidities among adolescents: a mini review | Meta-analysis/Literature review/Umbrella review/ Scoping Review |
| 286 | Translating Mechanism-Based Strategies to Break the Obesity−Cancer Link: A Narrative Review | Meta-analysis/Literature review/Umbrella review/ Scoping Review |
| 287 | Intermittent fasting interventions for treatment of overweight and obesity in adults: a systematic review and meta-analysis | Meta-analysis/Literature review/Umbrella review/ Scoping Review |
| 288 | Implications of Ramadan intermittent fasting on maternal and fetal health and nutritional status: A review | Meta-analysis/Literature review/Umbrella review/ Scoping Review |
| 289 | Time-restricted feeding and risk of metabolic disease: A review of human and animal studies | Meta-analysis/Literature review/Umbrella review/ Scoping Review |
| 290 | Time-restricted eating for patients with diabetes and prediabetes: A systematic review | Meta-analysis/Literature review/Umbrella review/ Scoping Review |
| 291 | Maintain host health with time-restricted eating and phytochemicals: A review based on gut microbiome and circadian rhythm | Meta-analysis/Literature review/Umbrella review/ Scoping Review |
| 292 | Intermittent fasting and caloric restriction for weight loss: a systematic review | Meta-analysis/Literature review/Umbrella review/ Scoping Review |
| 293 | Effect of Different Types of Intermittent Fasting on Biochemical and Anthropometric Parameters among Patients with Metabolic-Associated Fatty Liver Disease (MAFLD)-A Systematic Review | Meta-analysis/Literature review/Umbrella review/ Scoping Review |
| 294 | Impact of intermittent fasting duration on diabetes mellitus type 2: a systematic review | Meta-analysis/Literature review/Umbrella review/ Scoping Review |
| 295 | The Implication of Physiological Ketosis on The Cognitive Brain: A Narrative Review | Meta-analysis/Literature review/Umbrella review/ Scoping Review |
| 296 | Beneficial Effects of Time-Restricted Eating on Metabolic Diseases: A Systemic Review and Meta-Analysis | Meta-analysis/Literature review/Umbrella review/ Scoping Review |
| 297 | The Window Matters: A Systematic Review of Time Restricted Eating Strategies in Relation to Cortisol and Melatonin Secretion | Meta-analysis/Literature review/Umbrella review/ Scoping Review |
| 298 | Is time-restricted eating (8/16) beneficial for body weight and metabolism of obese and overweight adults? A systematic review and meta-analysis of randomized controlled trials | Meta-analysis/Literature review/Umbrella review/ Scoping Review |
| 299 | Intermittent fasting and weight loss: Systematic review | Meta-analysis/Literature review/Umbrella review/ Scoping Review |
| 300 | The Effect of Time-Restricted Eating on Insulin Levels and Insulin Sensitivity in Patients with Polycystic Ovarian Syndrome: A Systematic Review | Meta-analysis/Literature review/Umbrella review/ Scoping Review |
| 301 | Effects of Time-Restricted Feeding and Ramadan Fasting on Body Weight, Body Composition, Glucose Responses, and Insulin Resistance: A Systematic Review of Randomized Controlled Trials | Meta-analysis/Literature review/Umbrella review/ Scoping Review |
| 302 | The Effectiveness of Intermittent Fasting, Time Restricted Feeding, Caloric Restriction, a Ketogenic Diet and the Mediterranean Diet as Part of the Treatment Plan to Improve Health and Chronic Musculoskeletal Pain: A Systematic Review | Meta-analysis/Literature review/Umbrella review/ Scoping Review |
| 303 | Efficacy of Ketogenic Diets on Type 2 Diabetes: a Systematic Review | Meta-analysis/Literature review/Umbrella review/ Scoping Review |
| 304 | Intermittent Fasting and the Possible Benefits in Obesity, Diabetes, and Multiple Sclerosis: A Systematic Review of Randomized Clinical Trials | Meta-analysis/Literature review/Umbrella review/ Scoping Review |
| 305 | Effects of Intermittent Fasting on Specific Exercise Performance Outcomes: A Systematic Review Including Meta-Analysis | Meta-analysis/Literature review/Umbrella review/ Scoping Review |
| 306 | Health effects of intermittent fasting: hormesis or harm? A systematic review | Meta-analysis/Literature review/Umbrella review/ Scoping Review |
| 307 | Effects of time-restricted feeding with different feeding windows on metabolic health: A systematic review of human studies | Meta-analysis/Literature review/Umbrella review/ Scoping Review |
| 308 | Effect of Intermittent Fasting Diet on Glucose and Lipid Metabolism and Insulin Resistance in Patients with Impaired Glucose and Lipid Metabolism: A Systematic Review and Meta-Analysis | Meta-analysis/Literature review/Umbrella review/ Scoping Review |
| 309 | Effects of intermittent fasting combined with resistance training on body composition: a systematic review and meta-analysis | Meta-analysis/Literature review/Umbrella review/ Scoping Review |
| 310 | Effects of Intermittent Fasting in Human Compared to a Non-intervention Diet and Caloric Restriction: A Meta-Analysis of Randomized Controlled Trials | Meta-analysis/Literature review/Umbrella review/ Scoping Review |
| 311 | Intermittent food restriction upregulates critical hypothalamic genes involved in energy regulation imbalance | Different topic of interesting |
| 312 | Designing a Co-created Intervention to Promote Motivation and Maintenance of Time-Restricted Eating in Individuals With Overweight and Type 2 Diabetes | Different topic of interesting |
| 313 | Molecular mechanisms underlying the beneficial effects of exercise and dietary interventions in the prevention of cardiometabolic diseases | Different topic of interesting |
| 314 | Monitoring body composition change for intervention studies with advancing 3D optical imaging technology in comparison to dual-energy X-ray absorptiometry | Different topic of interesting |
| 315 | Intermittent fasting with ketogenic diet: A combination approach for management of chronic diseases | Different topic of interesting |
| 316 | Effects of different weight loss dietary interventions on body mass index and glucose and lipid metabolism in obese patients | Different topic of interesting |
| 317 | A Large-Scale Observational Analysis of Social Media Data Reveals Major Public Misperception of the Attainability of Drastic Weight Loss by Dieting | Different topic of interesting |
| 318 | Alternate-Day Fasting Combined with Exercise: Effect on Sleep in Adults with Obesity and NAFLD | Different topic of interesting |
| 319 | Screening the effective components in treating dampness stagnancy due to spleen deficiency syndrome and elucidating the potential mechanism of Poria water extract | Different topic of interesting |
| 320 | Circadian-mediated regulation of cardiometabolic disorders and aging with time-restricted feeding | Different topic of interesting |
| 321 | Impact of early time-restricted eating on diet quality, meal frequency, appetite, and eating behaviors: A randomized trial | Different topic of interesting |
| 322 | Intermittent fasting reverses the declining quality of aged oocytes | Different topic of interesting |
| 323 | Early time-restricted feeding improves high-density lipoprotein amount and function in nonhuman primates, without effects on body composition | Different topic of interesting |
| 324 | Effect of Islamic Alternate-Day Fasting (Daud Fasting) on Body Weight, Body Fat, and Skeletal Muscle in Male and Female Obese Young Adults in Dramaga, Bogor, Indonesia | Different topic of interesting |
| 325 | Examination of The Relationship Between Intermittent Fasting and Irisine Levels In Rats Fed A High-Fat Diet | Different topic of interesting |
| 326 | Intermittent fasting in type 2 diabetes: from fundamental science to clinical applications | Different topic of interesting |
| 327 | Intermittent fasting increases growth differentiation factor 15 in females with overweight or obesity but not associated with food intake | Different topic of interesting |
| 328 | Sociocultural influences on dietary behavior and meal timing among Native Hawaiian and Pacific Islander women at risk of endometrial cancer: a qualitative investigation | Different topic of interesting |
| 329 | The effects of three weight management methods on body composition and serum lipids of overweight and obese people | Different topic of interesting |
| 330 | Time-restricted feeding reduces monocyte production by controlling hematopoietic stem and progenitor cells in the bone marrow during obesity | Different topic of interesting |
| 331 | Metabolic effects of alternate-day fasting in males with obesity with or without type 2 diabetes | Different topic of interesting |
| 332 | Slimmer’s palsy following an intermittent fasting diet | Different topic of interesting |
| 333 | The basis and design for time-restricted eating compared with daily calorie restriction for weight loss and colorectal cancer risk reduction trial (TRE-CRC trial) | Different topic of interesting |
| 334 | Compliance of participants undergoing a ‘5-2’ intermittent fasting diet and impact on body weight | Different topic of interesting |
| 335 | Is time-restricted eating a robust eating regimen during periods of disruptions in daily life? A qualitative study of perspectives of people with overweight during COVID-19 | Different topic of interesting |
| 336 | Antipsychotic-induced weight gain and metabolic effects show diurnal dependence and are reversible with time restricted feeding | Different topic of interesting |
| 337 | Considering intermittent fasting among Saudis: insights into practices | Different topic of interesting |
| 338 | Efficacy and mechanism of intermittent fasting in metabolic associated fatty liver disease based on ultraperformance liquid chromatography-tandem mass spectrometry | Different topic of interesting |
| 339 | Time restricted feeding modifies leukocyte responsiveness and improves inflammation outcome | Different topic of interesting |
| 340 | Food odor perception promotes systemic lipid utilization | Different topic of interesting |
| 341 | Multifaceted Effects of Intermittent Fasting on the Treatment and Prevention of Diabetes, Cancer, Obesity or Other Chronic Diseases | Different topic of interesting |
| 342 | Siddha fasting in obese acute decompensated heart failure may improve hospital outcomes through empowerment and natural ketosis | Different topic of interesting |
| 343 | Intermittent fasting and time-restricted eating role in dietary interventions and precision nutrition | Different topic of interesting |
| 344 | A comparison of dietary quality and nutritional adequacy of popular energy-restricted diets against the Australian Guide to Healthy Eating and the Mediterranean Diet | Different topic of interesting |
| 345 | Intermittent fasting and exercise therapy abates STZ-induced diabetotoxicity in rats through modulation of adipocytokines hormone, oxidative glucose metabolic, and glycolytic pathway | Different topic of interesting |
| 346 | Time-Restricted Feeding Could Not Reduce Rainbow Trout Lipid Deposition Induced by Artificial Night Light | Different topic of interesting |
| 347 | Intermittent Fasting—Short- and Long-Term Quality of Life, Fatigue, and Safety in Healthy Volunteers: A Prospective, Clinical Trial | Different topic of interesting |
| 348 | Retention, Fasting Patterns, and Weight Loss With an Intermittent Fasting App: Large-Scale, 52-Week Observational Study | Different topic of interesting |
| 349 | Dietary recommendations for fasting days in an alternate-day intermittent fasting pattern: A randomized controlled trial | Different topic of interesting |
| 350 | Dietary recommendations for persons with type 2 diabetes mellitus | Different topic of interesting |
| 351 | Differences in Glucose Readings Between Right Arm and Left Arm Using a Continuous Glucose Monitor | Different topic of interesting |
| 352 | Overexpression of Pregnancy Zone Protein in Fat Antagonizes Diet-Induced Obesity Under an Intermittent Fasting Regime | Different topic of interesting |
| 353 | The effect of different weight loss strategies to treat non-alcoholic fatty liver disease focusing on fibroblast growth factor 21 | Different topic of interesting |
| 354 | Bibliometric and visual analysis of time-restricted eating | Different topic of interesting |
| 355 | Developing and Piloting a Novel Ranking System to Assess Popular Dietary Patterns and Healthy Eating Principles | Different topic of interesting |
| 356 | Psychiatric Futility and Palliative Care for a Patient with Clozapine-resistant Schizophrenia | Different topic of interesting |
| 357 | The Effect of Personality on Chrononutrition during the COVID-19 Lockdown in Qatar | Different topic of interesting |
| 358 | TRH and TRH-like peptide levels covary with caloric restriction and oral metformin in rat heart and testis | Different topic of interesting |
| 359 | Time-Restricted Feeding Studies and Possible Human Benefit | Different topic of interesting |
| 360 | Ketogenic dietary interventions in autosomal dominant polycystic kidney disease- A retrospective case series study: First insights into feasibility, safety and effects | Different topic of interesting |
| 361 | Following the Flow of Nature: The Microbiome and Intermittent Fasting Through the Lens of Traditional Chinese Medicine | Different topic of interesting |
| 362 | Mind the Gap: Exploring Nutritional Health Compared with Weight Management Interests of Individuals with Osteoarthritis | Different topic of interesting |
| 363 | Dietary patterns in non-alcoholic fatty liver disease (NAFLD): Stay on the straight and narrow path! [Patrones alimentarios en la enfermedad del hígado graso no alcohólico (EHGNA): ¡siga por el buen camino!] | Different topic of interesting |
| 364 | Intermittent fasting and continuous energy restriction result in similar changes in body composition and muscle strength when combined with a 12 week resistance training program | Different topic of interesting |
| 365 | Intermittent Fasting as Possible Treatment for Heart Failure | Different topic of interesting |
| 366 | Perspective: Time-Restricted Eating - Integrating the What with the When | Different topic of interesting |
| 367 | Time-Restricted Eating Regimen Differentially Affects Circulatory miRNA Expression in Older Overweight Adults | Different topic of interesting |
| 368 | Intermittent Fasting versus Continuous Calorie Restriction: Which Is Better for Weight Loss? | Different topic of interesting |
| 369 | Intermittent fasting enhances hippocampal NPY expression to promote neurogenesis after traumatic brain injury | Different topic of interesting |
| 370 | DIet and Health From reGIstered Trials on ClinicalTrials.gov: The DIGIT Study | Different topic of interesting |
| 371 | Acceptability of Time-Limited Eating in Pediatric Weight Management | Different topic of interesting |
| 372 | The Use of SGLT-2 Inhibitors Coupled With a Strict Low-Carbohydrate Diet: A Set-Up for Inducing Severe Diabetic Ketoacidosis | Different topic of interesting |
| 373 | Intermittent Fasting May Improve Health Outcomes in Overweight Adults | Different topic of interesting |
| 374 | Comparison of the effects of different dietary regimens on susceptibility to experimental acute kidney injury: The roles of SIRT1 and TGF-β1 | Different topic of interesting |
| 375 | Intermittent fasting may optimize intestinal microbiota, adipocyte status and metabolic health | Different topic of interesting |
| 376 | Intermittent Leucine Deprivation Produces Long-lasting Improvement in Insulin Sensitivity by Increasing Hepatic Gcn2 Expression | Different topic of interesting |
| 377 | Circadian timing of eating and BMI among adults in the American Time Use Survey | Different topic of interesting |
| 378 | Intermittent fasting: Eating by the clock for health and exercise performance | Different topic of interesting |
| 379 | Timing of Food/Nutrient Intake and Its Health Benefits | Different topic of interesting |
| 380 | The protective effect of intermittent fasting and physical exercise on obesity through changes in muscle diameter | Different topic of interesting |
| 381 | Dietary practices and supplement use among CrossFit® participants | Different topic of interesting |
| 382 | Effect of intermittent fasting on cardiovascular parameters of young adult offspring of hypertensive parents | Different topic of interesting |
| 383 | Intermittent fasting implementation and association with eating disorder symptomatology | Different topic of interesting |
| 384 | Intermittent Fasting Improves High-Fat Diet-Induced Obesity Cardiomyopathy via Alleviating Lipid Deposition and Apoptosis and Decreasing m6A Methylation in the Heart | Different topic of interesting |
| 385 | Dissociation between corneal and cardiometabolic changes in response to a time-restricted feeding of a high fat diet | Different topic of interesting |
| 386 | Extracts of Poria cocos improve functional dyspepsia via regulating brain-gut peptides, immunity and repairing of gastrointestinal mucosa | Different topic of interesting |
| 387 | The effect of intermittent diet and/or physical therapy in patients with chronic low back pain: A single-blinded randomized controlled trial | Different topic of interesting |
| 388 | Temporal eating patterns and eating windows among adults with overweight or obesity | Different topic of interesting |
| 389 | Reversion to regular diet with alternate day fasting can cure grade-I non-alcoholic fatty liver disease (NAFLD) in high-fructose-intake-associated metabolic syndrome | Different topic of interesting |
| 390 | Why we eat too much, have an easier time gaining than losing weight, and expend too little energy: Suggestions for counteracting or mitigating these problems | Different topic of interesting |
| 391 | Wikipedia, google trends and diet: Assessment of temporal trends in the internet users’ searches in italy before and during covid-19 pandemic | Different topic of interesting |
| 392 | Hepatokine Pregnancy Zone Protein Governs the Diet-Induced Thermogenesis Through Activating Brown Adipose Tissue | Different topic of interesting |
| 393 | Periodic fasting prevents fat penalties in females | Different topic of interesting |
| 394 | Weight loss barriers and dietary quality of intermittent and continuous dieters in women with a history of gestational diabetes | Different topic of interesting |
| 395 | To fast or not to fast — that is the question | Different topic of interesting |
| 396 | Effects of concurrent training and intermittent fasting on structural, functional, and morphological parameters of the heart [Efectos del entrenamiento concurrente y el ayuno intermitente sobre los parámetros estructurales, funcionales y morfológicos del corazón] | Different topic of interesting |
| 397 | Hepatic-Metabolite-Based Intermittent Fasting Enables a Sustained Reduction in Insulin Resistance in Type 2 Diabetes and Metabolic Syndrome | Different topic of interesting |
| 398 | Eight weeks of intermittent fasting versus calorie restriction does not alter eating behaviors, mood, sleep quality, quality of life and cognitive performance in women with overweight | Different topic of interesting |
| 399 | Differential weight loss with intermittent fasting or daily calorie restriction in low- and high-fitness phenotypes | Different topic of interesting |
| 400 | Late-life intermittent fasting decreases aging-related frailty and increases renal hydrogen sulfide production in a sexually dimorphic manner | Different topic of interesting |
| 401 | Nutritional basis of type 2 diabetes remission | Different topic of interesting |
| 402 | A relationship between mortality and eating breakfast and fiber | Different topic of interesting |
| 403 | Time-Restricted Feeding in Commercial Layer Chickens Improves Egg Quality in Old Age and Points to Lack of Adipostat Activity in Chickens | Different topic of interesting |
| 404 | Intermittent Fasting: Can It Help Optimize Human Performance? | Different topic of interesting |
| 405 | Treatment of NAFLD with intermittent calorie restriction or low-carb high-fat diet – a randomised controlled trial | Different topic of interesting |
| 406 | Weight loss, hypertension and mental well-being improvements during COVID-19 with a multicomponent health promotion programme on Zoom: A service evaluation in primary care | Different topic of interesting |
| 407 | Watching, keeping and squeezing time to lose weight: Implications of time-restricted eating in daily life | Different topic of interesting |
| 408 | The alternate-day fasting diet is a more effective approach than a calorie restriction diet on weight loss and hs-CRP levels | Different topic of interesting |
| 409 | An intermittent fasting mimicking nutrition bar extends physiologic ketosis in time restricted eating: A randomized, controlled, parallel-arm study | Different topic of interesting |
| 410 | Fasting: How to guide | Different topic of interesting |
| 411 | Article identification and validation of nutrient state-dependent serum protein mediators of human cd4+ t cell responsiveness | Different topic of interesting |
| 412 | Multiomic Predictors of Short-Term Weight Loss and Clinical Outcomes During a Behavioral-Based Weight Loss Intervention | Different topic of interesting |
| 413 | Protective Effect of Intestinal Helminthiasis Against Tuberculosis Progression Is Abrogated by Intermittent Food Deprivation | Different topic of interesting |
| 414 | Intermittent fasting on the ekadashiday and the role of spiritual nutrition | Different topic of interesting |
| 415 | Eating Timing: Associations with Dietary Intake and Metabolic Health | Different topic of interesting |
| 416 | Intermittent Fasting and Muscle Lipid Metabolism | Different topic of interesting |
| 417 | Physiology of weight regain: Lessons from the classic Minnesota Starvation Experiment on human body composition regulation | Different topic of interesting |
| 418 | Early versus late time-restricted feeding in adults at increased risk of developing type 2 diabetes: Is there an optimal time to eat for metabolic health? | Different topic of interesting |
| 419 | Does the weight loss efficacy of alternate day fasting differ according to sex and menopausal status? | Different topic of interesting |
| 420 | Pilot clinical trial of time-restricted eating in patients with metabolic syndrome | Different topic of interesting |
| 421 | Effect of Intermittent Fasting (18/6) on Energy Expenditure, Nutritional Status, and Body Composition in Healthy Adults | Different topic of interesting |
| 422 | Probiotics and high fiber-rich diets have anti-inflammatory properties and decline chronic kidney disease progression [Probiotici i dijetalna prehrana bogata vlaknima uz protuupalne učinke usporava i progresiju kronične bubrežne bolesti] | Different topic of interesting |
| 423 | Short-term fasting reshapes fat tissue | Different topic of interesting |
| 424 | Later meal and sleep timing predicts higher percent body fat | Different topic of interesting |
| 425 | Dietary and Pharmacological Interventions That Inhibit Mammalian Target of Rapamycin Activity Alter the Brain Expression Levels of Neurogenic and Glial Markers in an Age-and Treatment-Dependent Manner | Different topic of interesting |
| 426 | Associations of time-restricted eating with health-related quality of life and sleep in adults: a secondary analysis of two pre-post pilot studies | Different topic of interesting |
| 427 | Optimising intermittent fasting: Evaluating the behavioural and metabolic effects of extended morning and evening fasting | Different topic of interesting |
| 428 | Feasibility of weight loss in obese atrial fibrillation patients attending a specialist arrhythmia clinic and its impact on ablation outcomes | Different topic of interesting |
| 429 | Severely restricting energy intake for 24 h does not affect markers of bone metabolism at rest or in response to re-feeding | Different topic of interesting |
| 430 | Time-restricted eating alters food intake patterns, as prospectively documented by a smartphone application | Different topic of interesting |
| 431 | Organic Diet and Intermittent Fasting are Associated With Improved Erectile Function | Different topic of interesting |
| 432 | Continuous Glucose Monitoring of Glycemic Variability During Fasting Post-Sleeve Gastrectomy | Different topic of interesting |
| 433 | Alternate-day fasting alleviates diabetes-induced glycolipid metabolism disorders: roles of FGF21 and bile acids | Different topic of interesting |
| 434 | Food cravings: Associations with dietary intake and metabolic health | Different topic of interesting |
| 435 | Associations of number of daily eating occasions with type 2 diabetes risk in the women's health initiative dietary modification trial | Different topic of interesting |
| 436 | Intermittent fasting attenuates exercise training-induced cardiac remodeling [Dieta Intermitente Atenua a Remodelação Cardíaca Causada pelo Exercício Físico] | Different topic of interesting |
| 437 | Extending the overnight fast: Sex differences in acute metabolic responses to breakfast | Different topic of interesting |
| 438 | Therapeutic use of intermittent fasting and ketogenic diet as an alternative treatment for type 2 diabetes in a normal weight woman: A 14-month case study | Different topic of interesting |
| 439 | Free-Living Sleep, Food Intake, and Physical Activity in Night and Morning Shift Workers | Different topic of interesting |
| 440 | Metabolome of mammary tumors differs from normal mammary glands but is not altered by time-restricted feeding under obesogenic conditions | Different topic of interesting |
| 441 | Intermittent Fasting Does Not Uniformly Impact Genes Involved in Circadian Regulation in Women with Obesity | Different topic of interesting |
| 442 | Safety and feasibility of various fasting-mimicking diets among people with multiple sclerosis | Different topic of interesting |
| 443 | Calorie Restriction and Intermittent Fasting: Impact on Glycemic Control in People With Diabetes | Different topic of interesting |
| 444 | Small sided games vs repeated sprint training effects on agility in fasting basketball players [Jogos reduzidos vs sprints repetidos treinamento efietos na agilidade em jogadores de basquete em jejum] [Juegos reducidos vs sprints repetidos enternamiento efectos en la agilidad en jugadores de baloncesto en ayunas] | Different topic of interesting |
| 445 | Nonalcoholic Fatty Liver Disease in 2020 | Different topic of interesting |
| 446 | Effect of jain fasting on anthropometric, clinical and biochemical parameters | Different topic of interesting |
| 447 | Investigating Physical and Nutritional Changes During Prolonged Intermittent Fasting in Hemodialysis Patients: A Prospective Cohort Study | Different topic of interesting |
| 448 | Intermittent Fasting as a Trigger of Ketoacidosis in a Patient with Stable, Long-term Type 1 Diabetes | Different topic of interesting |
| 449 | Influence of Long-term Fasting and Intermittent Fasting on the Cognitive Abilities | Different topic of interesting |
| 450 | Philippine consensus statement on the use of ketogenic diet and intermittent fasting diet on adults for weight reduction | Different topic of interesting |
| 451 | Dieting, mindfulness and mindful eating: Exploring whether or not diets reinforce mindfulness and mindful eating practices | Different topic of interesting |
| 452 | Weight loss efficacy of alternate day fasting versus daily calorie restriction in subjects with subclinical hypothyroidism: A secondary analysis | Different topic of interesting |
| 453 | Voluntary exercise is motivated by ghrelin, possibly related to the central reward circuit | Different topic of interesting |
| 454 | Scientific evidence of diets for weight loss: Different macronutrient composition, intermittent fasting, and popular diets | Different topic of interesting |
| 455 | The role of intermittent fasting and meal timing in weight management and metabolic health | Different topic of interesting |
| 456 | Effects of alternate-day fasting on body weight and dyslipidaemia in patients with non-alcoholic fatty liver disease: A randomised controlled trial | Different topic of interesting |
| 457 | Mediterranean journal of rheumatology december 2019 highlights | Different topic of interesting |
| 458 | Modified alternate-day fasting vs. calorie restriction in the treatment of patients with metabolic syndrome: A randomized clinical trial | Different topic of interesting |
| 459 | New Zealand bitter hops extract reduces hunger during a 24 h water only fast | Different topic of interesting |
| 460 | Energy balance and obesity in individuals with cystic fibrosis | Different topic of interesting |
| 461 | Effects of day-time feeding on murine skeletal muscle growth and synthesis | Different topic of interesting |
| 462 | Beverage intake during alternate-day fasting: Relationship to energy intake and body weight | Different topic of interesting |
| 463 | Influence of intermittent fasting on myocardial infarction-induced cardiac remodeling | Different topic of interesting |
| 464 | Clinical management of intermittent fasting in patients with diabetes mellitus | Different topic of interesting |
| 465 | Effects of alternate day fasting and exercise on cholesterol metabolism in overweight or obese adults: A pilot randomized controlled trial | Different topic of interesting |
| 466 | Ketogenic diet rescues cognition in ApoE4+ patient with mild Alzheimer's disease: A case study | Different topic of interesting |
| 467 | Pilot study of novel intermittent fasting effects on metabolomic and trimethylamine N-oxide changes during 24-hour water-only fasting in the FEELGOOD trial | Different topic of interesting |
| 468 | New perspectives on chrononutrition | Different topic of interesting |
| 469 | Starvation ketoacidosis due to the ketogenic diet and prolonged fasting - A possibly dangerous diet trend | Different topic of interesting |
| 470 | Differential Effects of Alternate-Day Fasting Versus Daily Calorie Restriction on Insulin Resistance | Different topic of interesting |
| 471 | Intermittent Fasting - What Is It and Does It Work? | Different topic of interesting |
| 472 | Intermittent fasting increases SOD2 and catalase immunoreactivities in the hippocampus but does not protect from neuronal death following transient ischemia in gerbils | Different topic of interesting |
| 473 | Effects of alternate-day fasting or daily calorie restriction on body composition, fat distribution, and circulating adipokines: Secondary analysis of a randomized controlled trial | Different topic of interesting |
| 474 | 24-h severe energy restriction impairs postprandial glycaemic control in young, lean males | Different topic of interesting |
| 475 | Investigating of the effects of orthodox christian fasting on human health | Different topic of interesting |
| 476 | Effects of intermittent and continuous calorie restriction on body weight and metabolism over 50 wk: A randomized controlled trial | Different topic of interesting |
| 477 | Endogenous secretory RAGE increases with improvements in body composition and is associated with markers of adipocyte health | Different topic of interesting |
| 478 | Lysosomes Mediate Benefits of Intermittent Fasting in Cardiometabolic Disease: The Janitor Is the Undercover Boss | Different topic of interesting |
| 479 | Limiting feeding to the active phase reduces blood pressure without the necessity of caloric reduction or fat mass loss | Different topic of interesting |
| 480 | Gut flora shift caused by time-restricted feeding might protect the host from metabolic syndrome, inflammatory bowel disease and colorectal cancer | Different topic of interesting |
| 481 | Randomized trial of a high protein, partial meal replacement program with or without alternate day fasting: Similar effects on weight loss, retention status, nutritional, metabolic, and behavioral outcomes | Different topic of interesting |
| 482 | The effect of 10 days of intermittent fasting on Wingate anaerobic power and prolonged high-intensity time-to-exhaustion cycling performance | Different topic of interesting |
| 483 | Therapeutic use of intermittent fasting for people with type 2 diabetes as an alternative to insulin | Different topic of interesting |
| 484 | Intermittent fasting: Less weight, hardly any sacrifice? [Weniger gewicht, kaum verzicht?] | Different topic of interesting |
| 485 | The effects of diurnal intermittent fasting on the wake-promoting neurotransmitter orexin-A | Different topic of interesting |
| 486 | The effect of intermittent fasting on blood pressure variability in patients with newly diagnosed hypertension or prehypertension | Different topic of interesting |
| 487 | Intermittent fasting and cardiovascular disease: Current evidence and unresolved questions | Different topic of interesting |
| 488 | Bidirectional Regulation of Circadian Disturbance and Inflammation in Inflammatory Bowel Disease | Different topic of interesting |
| 489 | Fasting as possible complementary approach for polycystic ovary syndrome: Hope or hype? | Different topic of interesting |
| 490 | Effect of alternate-day fasting on weight loss, weight maintenance, and cardioprotection among metabolically healthy obese adults: A randomized clinical trial | Different topic of interesting |
| 491 | The Effect of Alternate-Day Fasting (ADF) on Weight Loss, Metabolic Parameters and Psychological Characteristics | Different topic of interesting |
| 492 | Intermittent food restriction initiated late in life prolongs lifespan and retards the onset of age-related markers in the annual fish Nothobranchius guentheri | Different topic of interesting |
| 493 | Comparison of intermittent fasting versus caloric restriction in obese subjects: A two year follow-up | Different topic of interesting |
| 494 | Is There an Optimal Diet for Weight Management and Metabolic Health? | Different topic of interesting |
| 495 | Relationship between FGF21 and UCP1 levels under time-restricted feeding and high-fat diet | Different topic of interesting |
| 496 | Serum polychlorinated biphenyls increase and oxidative stress decreases with a protein-pacing caloric restriction diet in obese men and women | Different topic of interesting |
| 497 | Effects of intermittent fasting and chronic swimming exercise on body composition and lipid metabolism | Different topic of interesting |
| 498 | Massive obesity treated by intermittent fasting. A metabolic and clinical study | Different topic of interesting |
| 499 | Early Time-Restricted Feeding Improves Insulin Sensitivity, Blood Pressure, and Oxidative Stress Even without Weight Loss in Men with Prediabetes | Different topic of interesting |
| 500 | Weight loss with a low-carbohydrate, Mediterranean, or low-fat diet | Different topic of interesting |
| 501 | Ten-Hour Time-Restricted Eating Reduces Weight, Blood Pressure, and Atherogenic Lipids in Patients with Metabolic Syndrome | Different topic of interesting |
| 502 | Randomized controlled trial for time-restricted eating in healthy volunteers without obesity | Different topic of interesting |
| 503 | Eight-hour time-restricted feeding improves endocrine and metabolic profiles in women with anovulatory polycystic ovary syndrome | Different topic of interesting |
| 504 | Effects of eight weeks of time-restricted feeding (16/8) on basal metabolism, maximal strength, body composition, inflammation, and cardiovascular risk factors in resistance-trained males | Different topic of interesting |
| 505 | Effects of time-restricted feeding in weight loss, metabolic syndrome and cardiovascular risk in obese women | Different topic of interesting |
| 506 | Prolonged, Controlled Daytime versus Delayed Eating Impacts Weight and Metabolism | Different topic of interesting |
| 507 | Time-restricted feeding alters lipid and amino acid metabolite rhythmicity without perturbing clock gene expression | Different topic of interesting |
| 508 | Effect of Intermittent Compared With Continuous Energy Restricted Diet on Glycemic Control in Patients With Type 2 Diabetes: A Randomized Noninferiority Trial | Different topic of interesting |
| 509 | Isocaloric-restricted Mediterranean Diet and Chinese Diets High or Low in Plants in Adults With Prediabetes | Different topic of interesting |
| 510 | Time-restricted eating with or without low-carbohydrate diet reduces visceral fat and improves metabolic syndrome: A randomized trial | Different topic of interesting |
| 511 | Intermittent fasting from dawn to sunset for four consecutive weeks induces anticancer serum proteome response and improves metabolic syndrome | Different topic of interesting |
| 512 | Time-restricted eating and exercise training improve HbA1c and body composition in women with overweight/obesity: A randomized controlled trial | Different topic of interesting |
| 513 | Effects of 1 year of exercise training versus combined exercise training and weight loss on body composition, low-grade inflammation and lipids in overweight patients with coronary artery disease: a randomized trial | Different topic of interesting |
| 514 | Influence of sleep restriction on weight loss outcomes associated with caloric restriction | Different topic of interesting |
| 515 | 2 years of calorie restriction and cardiometabolic risk (CALERIE): exploratory outcomes of a multicentre, phase 2, randomised controlled trial | Different topic of interesting |
| 516 | Efficacy and Safety of Intermittent Fasting in People With Insulin-Treated Type 2 Diabetes (INTERFAST-2)-A Randomized Controlled Trial | Different topic of interesting |
| 517 | Time-Restricted Feeding Improves Glucose Tolerance in Men at Risk for Type 2 Diabetes: A Randomized Crossover Trial | Different topic of interesting |
| 518 | Low versus high carbohydrate diet in type 1 diabetes: A 12-week randomized open-label crossover study | Different topic of interesting |
| 519 | Effect of intermittent compared to continuous energy restriction on weight loss and weight maintenance after 12 months in healthy overweight or obese adults | Different topic of interesting |
| 520 | Intermittent calorie restriction alters T cell subsets and metabolic markers in people with multiple sclerosis | Different topic of interesting |
| 521 | Associations between the timing of eating and weight-loss in calorically restricted healthy adults: Findings from the CALERIE study | Different topic of interesting |
| 522 | A Low-Carbohydrate Diet Realizes Medication Withdrawal: A Possible Opportunity for Effective Glycemic Control | Different topic of interesting |
| 523 | The Effects of a Macronutrient-Based Diet and Time-Restricted Feeding (16:8) on Body Composition in Physically Active Individuals-A 14-Week Randomised Controlled Trial | Different topic of interesting |
| 524 | Time restricted eating as a weight loss intervention in adults with obesity | Different topic of interesting |
| 525 | Adherence to Diet and Meal Timing in a Randomized Controlled Feeding Study of Time-Restricted Feeding | Different topic of interesting |
| 526 | Metabolic syndrome and the benefit of a physical activity intervention on lower-extremity function: Results from a randomized clinical trial | Different topic of interesting |
| 527 | A Low-Calorie Diet with or without Exercise Reduces Postprandial Aortic Waveform in Females with Obesity | Different topic of interesting |
| 528 | Non-Energy-Restricted Low-Carbohydrate Diet Combined with Exercise Intervention Improved Cardiometabolic Health in Overweight Chinese Females | Different topic of interesting |
| 529 | Effectiveness of restricted diet with a plate in patients with type 2 diabetes: A randomized controlled trial | Different topic of interesting |
| 530 | A randomized pilot study comparing zero-calorie alternate-day fasting to daily caloric restriction in adults with obesity | Alternate-Day Fasting |
| 531 | Determinants of weight loss success with alternate day fasting | Alternate-Day Fasting |
| 532 | Effects of intermittent fasting on body composition and clinical health markers in humans | Alternate-Day Fasting |
| 533 | Short-term modified alternate-day fasting: A novel dietary strategy for weight loss and cardioprotection in obese adults | Alternate-Day Fasting |
| 534 | Safety of alternate day fasting and effect on disordered eating behaviors | Alternate-Day Fasting |
| 535 | Meal timing during alternate day fasting: Impact on body weight and cardiovascular disease risk in obese adults | Alternate-Day Fasting |
| 536 | Alternate day fasting for weight loss in normal weight and overweight subjects: A randomized controlled trial | Alternate-Day Fasting |
| 537 | Alternate day fasting with or without exercise: Effects on endothelial function and adipokines in obese humans | Alternate-Day Fasting |
| 538 | Alternate day fasting and endurance exercise combine to reduce body weight and favorably alter plasma lipids in obese humans | Alternate-Day Fasting |
| 539 | Alternate day fasting increases LDL particle size independently of dietary fat content in obese humans | Alternate-Day Fasting |
| 540 | Benefit of a low-fat over high-fat diet on vascular health during alternate day fasting | Alternate-Day Fasting |
| 541 | The effects of modified alternate-day fasting diet on weight loss and CAD risk factors in overweight and obese women | Alternate-Day Fasting |
| 542 | Alternate day fasting (ADF) with a high-fat diet produces similar weight loss and cardio-protection as ADF with a low-fat diet | Alternate-Day Fasting |
| 543 | Improvements in coronary heart disease risk indicators by alternate-day fasting involve adipose tissue modulations | Alternate-Day Fasting |
| 544 | Improvements in body fat distribution and circulating adiponectin by alternate-day fasting versus calorie restriction | Alternate-Day Fasting |
| 545 | Modified alternate-day fasting and cardioprotection: relation to adipose tissue dynamics and dietary fat intake | Alternate-Day Fasting |
| 546 | Alternate-day fasting in nonobese subjects: Effects on body weight, body composition, and energy metabolism | Alternate-Day Fasting |
| 547 | Effect of alternate day fasting combined with aerobic exercise on non-alcoholic fatty liver disease: A randomized controlled trial | Alternate-Day Fasting |
| 548 | Sustained alternate-day fasting potentiates doxorubicin cardiotoxicity | Alternate-Day Fasting |
| 549 | Alternate Day Fasting Improves Physiological and Molecular Markers of Aging in Healthy, Non-obese Humans | Alternate-Day Fasting |
| 550 | Oral iron supplements increase hepcidin and decrease iron absorption from daily or twice-daily doses in iron-depleted young women | Alternate-Day Fasting |
| 551 | Effects of weight loss via high fat vs. low fat alternate day fasting diets on free fatty acid profiles | Alternate-Day Fasting |
| 552 | The effect of alternate-day caloric restriction on the metabolic consequences of 8 days of bed rest in healthy lean men: a randomized trial | Alternate-Day Fasting |
| 553 | A Randomised Controlled Trial on the Effectiveness and Adherence of Modified Alternate-day Calorie Restriction in Improving Activity of Non-Alcoholic Fatty Liver Disease | Alternate-Day Fasting |
| 554 | Effect of Airborne Low Intensity Multi frequency ultrasound (ALIMFUS) on glycemic control, lipid profile and markers of inflammation in patients with uncontrolled type 2 diabetes: A multicentre proof of concept, randomized double blind Placebo controlled study | Alternate-Day Fasting |
| 555 | Effect of 24-h severe energy restriction on appetite regulation and ad libitum energy intake in lean men and women | Alternate-Day Fasting |
| 556 | Outcomes of Desidustat Treatment in People with Anemia and Chronic Kidney Disease: A Phase 2 Study | Alternate-Day Fasting |
| 557 | Investigation into the acute effects of total and partial energy restriction on postprandial metabolism among overweight/obese participants | Alternate-Day Fasting |
| 558 | Intermittent fasting for the prevention of cardiovascular disease | Alternate-Day Fasting |
| 559 | Effect of Various Types of Intermittent Fasting (IF) on Weight Loss and Improvement of Diabetic Parameters in Human | Alternate-Day Fasting |
| 560 | Fasting for weight loss: an effective strategy or latest dieting trend? | Alternate-Day Fasting |
| 561 | Intermittent fasting vs daily calorie restriction for type 2 diabetes prevention: a review of human findings | Alternate-Day Fasting |
| 562 | Effect of modified alternate day fasting diet on the severity of premenstrual syndrome and health-related quality of life in women with overweight or obesity: a trial study protocol | Alternate-Day Fasting |
| 563 | Alternate-day fasting diet improves fructose-induced insulin resistance in mice | Alternate-Day Fasting |
| 564 | Corrigendum to "Effects of alternate-day fasting, time-restricted fasting and intermittent energy restriction DSS-induced on colitis and behavioral disorders" [Redox Biology 32, 2020, 101535] | Alternate-Day Fasting |
| 565 | Changes in hunger and fullness in relation to gut peptides before and after 8 weeks of alternate day fasting | Alternate-Day Fasting |
| 566 | Sample Size Matters When Drawing Conclusions on Alternate-Day Fasting Diet | Alternate-Day Fasting |
| **B. Full text screening** | | |
| 1 | Macronutrient composition and its effect on body composition changes during weight loss therapy in patients with non-alcoholic fatty liver disease: Secondary analysis of a randomized controlled trial | The 5:2 diet and other diets |
| 2 | Design and Implementation of a Time-Restricted Eating Intervention in a Randomized, Controlled Eating Study | Different topic of interesting |
| 3 | Feasibility and outcomes from using a commitment devices and text message reminders to increase adherence to time-restricted eating: A randomized trial | Inappropriate data |
| 4 | Time-restricted eating improves measures of daily glycaemic control in people with type 2 diabetes | Inappropriate data |
| 5 | Group differences in binge eating, impulsivity, and intuitive and mindful eating among intermittent fasters and non-fasters | Different topic of interesting |
| 6 | Association of Eating and Sleeping Intervals With Weight Change Over Time: The Daily24 Cohort | Different topic of interesting |
| 7 | An Intervention of Four Weeks of Time-Restricted Eating (16/8) in Male Long-Distance Runners Does Not Affect Cardiometabolic Risk Factors | Inappropriate data |
| 8 | Efficacy and Safety of Intermittent Fasting in People With Insulin-Treated Type 2 Diabetes (INTERFAST-2)—A Randomized Controlled Trial | Different topic of interesting |
| 9 | Intermittent fasting and protein pacing are superior to caloric restriction for weight and visceral fat loss | Different topic of interesting |
| 10 | Eight-hour time-restricted eating does not lower daily myofibrillar protein synthesis rates: A randomized control trial | Inappropriate data |
| 11 | Time-restricted eating did not alter insulin sensitivity or β-cell function in adults with obesity: A randomized pilot study | Inappropriate data |
| 12 | Early time-restricted eating affects weight, metabolic health, mood, and sleep in adherent completers: A secondary analysis | Duplicated population |
| 13 | The effects of time-restricted eating and weight loss on bone metabolism and health: a 6-month randomized controlled trial | Inappropriate data |
| 14 | Effect of time-restricted eating on sex hormone levels in premenopausal and postmenopausal females | Different topic of interesting |
| 15 | Time–restricted eating alters the 24-hour profile of adipose tissue transcriptome in men with obesity | Inappropriate data |
| 16 | The Value of Intermittent Fasting and Low Carbohydrate Diet in Prediabetic Patients for the Prevention of Cardiovascular Diseases [Papel do Jejum Intermitente e da Dieta Restrita em Carboidratos na Prevenção de Doenças Cardiovasculares em Pacientes Pré-Diabéticos] | Different topic of interesting |
| 17 | Fasting and weight loss: mobile application-based approach | Different topic of interesting |
| 18 | Early time-restricted eating may favorably impact cognitive acuity in university students: a randomized pilot study | Different topic of interesting |
| 19 | Craving for carbs: food craving and disordered eating in low-carb dieters and its association with intermittent fasting | Different topic of interesting |
| 20 | Intermittent fasting two days versus one day per week, matched for total energy intake and expenditure, increases weight loss in overweight/obese men and women | Different topic of interesting |
| 21 | The impact of a self-selected time restricted eating intervention on eating patterns, sleep, and late-night eating in individuals with obesity | Different topic of interesting |
| 22 | The effect of intermittent fasting diets on body weight and composition | Different topic of interesting |
| 23 | ORIGINAL ARTICLE Effects of Intermittent Fasting on Weight Loss in Asian Indian Adults with Obesity | Inappropriate data |
| 24 | Effectiveness of Early Time-Restricted Eating for Weight Loss, Fat Loss, and Cardiometabolic Health in Adults with Obesity: A Randomized Clinical Trial | Duplicated population |
| 25 | Intermittent fasting positively modulates human gut microbial diversity and ameliorates blood lipid profile | Different topic of interesting |
| 26 | The Effects of Intermittent Fasting and Continuous Energy Restriction with Exercise on Cardiometabolic Biomarkers, Dietary Compliance, and Perceived Hunger and Mood: Secondary Outcomes of a Randomised, Controlled Trial | The 5:2 diet and other diets |
| 27 | Effects of Intermittent Energy Restriction Alone and in Combination with Sprint Interval Training on Body Composition and Cardiometabolic Biomarkers in Individuals with Overweight and Obesity | The 5:2 diet and other diets |
| 28 | What happens after a weight loss intervention? A qualitative study of drivers and challenges of maintaining time-restricted eating among people with overweight at high risk of type 2 diabetes | Different topic of interesting |
| 29 | Time-Restricted Eating to Reduce Cardiovascular Risk Among Older Breast Cancer Survivors: A Single-Arm Feasibility Study | Inappropriate data |
| 30 | Effects of Time-Restricted Feeding on Energy Balance: A Cross-Over Trial in Healthy Subjects | Different topic of interesting |
| 31 | Fasting-Mimicking Diet Reduces Trimethylamine N-Oxide Levels and Improves Serum Biochemical Parameters in Healthy Volunteers | Different topic of interesting |
| 32 | Intermittent fasting in weight loss and cardiometabolic risk reduction: A randomized controlled trial | Inappropriate data |
| 33 | Differential Effects of One Meal per Day in the Evening on Metabolic Health and Physical Performance in Lean Individuals | Different topic of interesting |
| 34 | Time-restricted feeding and brisk walking in overweight and obese adults | Inappropriate data |
| 35 | Early or delayed onset of food intake in time‐restricted eating: Associations with markers of obesity in a secondary analysis of two pilot studies | Different topic of interesting |
| 36 | The effect of intermittent energy restriction on weight loss and diabetes risk markers in women with a history of gestational diabetes: a 12-month randomized control trial | Different topic of interesting |
| 37 | Effects of time-restricted feeding on supramaximal exercise performance and body composition: A randomized and counterbalanced crossover study in healthy men | Different topic of interesting |
| 38 | A randomized controlled trial to isolate the effects of fasting and energy restriction on weight loss and metabolic health in lean adults | Different topic of interesting |
| 39 | Effect of high-intensity interval training and intermittent fasting on body composition and physical performance in active women | Different topic of interesting |
| 40 | Seven-day fasting as a multimodal complex intervention for adults with type 1 diabetes: Feasibility, benefit and safety in a controlled pilot study | Different topic of interesting |
| 41 | Time-restricted eating and concurrent exercise training reduces fat mass and increases lean mass in overweight and obese adults | Inappropriate data |
| 42 | Time-restricted feeding and metabolic outcomes in a cohort of Italian adults | Inappropriate data |
| 43 | Time-restricted eating improves quality of life measures in overweight humans | Different topic of interesting |
| 44 | Intermittent and continuous energy restriction result in similar weight loss, weight loss maintenance, and body composition changes in a 6 month randomized pilot study | Different topic of interesting |
| 45 | New obesity treatment: Fasting, exercise and low carb diet - The NOT-FED study | Inappropriate data |
| 46 | Effects of Intermittent Fasting or Calorie Restriction on Markers of Lipid Metabolism in Human Skeletal Muscle | Different topic of interesting |
| 47 | Effects of time-restricted feeding on body weight, body composition and vital signs in low-income women with obesity: A 12-month randomized clinical trial | Inappropriate data |
| 48 | ERGO2: A Prospective, Randomized Trial of Calorie-Restricted Ketogenic Diet and Fasting in Addition to Reirradiation for Malignant Glioma | Different topic of interesting |
| 49 | Profast: A randomized trial assessing the effects of intermittent fasting and lacticaseibacillus rhamnosus probiotic among people with prediabetes | The 5:2 diet and other diets |
| 50 | Time-restricted eating as a nutrition strategy for individuals with type 2 diabetes: A feasibility study | Inappropriate data |
| 51 | The impact of time-restricted diet on sleep and metabolism in obese volunteers | Different topic of interesting |
| 52 | Intermittent fasting enhanced the cognitive function in older adults with mild cognitive impairment by inducing biochemical and metabolic changes: A 3-year progressive study | Different topic of interesting |
| 53 | Acute effects of time-restricted feeding in low-income women with obesity placed on hypoenergetic diets: Randomized trial | Inappropriate data |
| 54 | Efficacy of an intermittent energy restriction diet in a primary care setting | Different topic of interesting |
| 55 | Effect of a six-week intermittent fasting intervention program on the composition of the human body in women over 60 years of age | Inappropriate data |
| 56 | Intermittent energy restriction is comparable to continuous energy restriction for cardiometabolic health in adults with central obesity: A randomized controlled trial; the Met-IER study | Different topic of interesting |
| 57 | Short-term time-restricted feeding is safe and feasible in non-obese healthy midlife and older adults | Inappropriate data |
| 58 | Determinants of adherence in time-restricted feeding in older adults: Lessons from a pilot study | Different topic of interesting |
| 59 | Matched Weight Loss Through Intermittent or Continuous Energy Restriction Does Not Lead to Compensatory Increases in Appetite and Eating Behavior in a Randomized Controlled Trial in Women with Overweight and Obesity | Inappropriate data |
| 60 | Intermittent fasting, Paleolithic, or Mediterranean diets in the real world: Exploratory secondary analyses of a weight-loss trial that included choice of diet and exercise | Different topic of interesting |
| 61 | Energy restriction enhances adult hippocampal neurogenesis-associated memory after four weeks in an adult human population with central obesity; a randomized controlled trial | Different topic of interesting |
| 62 | Time-restricted feeding improves markers of cardiometabolic health in physically active college-age men: a 4-week randomized pre-post pilot study | Inappropriate data |
| 63 | Adherence to time-restricted feeding and impact on abdominal obesity in primary care patients: Results of a pilot study in a pre–post design | Duplicated population |
| 64 | Time-restricted feeding plus resistance training in active females: A randomized trial | Different topic of interesting |
| 65 | Early Time-Restricted Feeding Reduces Appetite and Increases Fat Oxidation But Does Not Affect Energy Expenditure in Humans | Different topic of interesting |
| 66 | Markers of adipose tissue inflammation are transiently elevated during intermittent fasting in women who are overweight or obese | Different topic of interesting |
| 67 | Early time-restricted feeding improves 24-hour glucose levels and affects markers of the circadian clock, aging, and autophagy in humans | Inappropriate data |
| 68 | The effect of intermittent compared with continuous energy restriction on glycaemic control in patients with type 2 diabetes: 24-month follow-up of a randomised noninferiority trial | The 5:2 diet and other diets |
| 69 | The differential response to intermittent fasting diet versus low calorie diet with exercise based on -866 G/A UCP2 gene variation in adults with overweight/obesity | Different topic of interesting |
| 70 | Safety of 8-h time restricted feeding in adults with obesity | Inappropriate data |
| 71 | Effects of Intermittent Versus Continuous Energy Intakes on Insulin Sensitivity and Metabolic Risk in Women with Overweight | Different topic of interesting |
| 72 | The safety and efficacy of weight loss via intermittent fasting or standard daily energy restriction in adults with type 1 diabetes and overweight or obesity: A pilot study | Different topic of interesting |
| 73 | Effect of 5:2 Fasting Diet on Liver Fat Content in Patients with Type 2 Diabetic with Nonalcoholic Fatty Liver Disease | The 5:2 diet and other diets |
| 74 | Effect of intermittent versus continuous energy restriction on weight loss, maintenance and cardiometabolic risk: A randomized 1-year trial | The 5:2 diet and other diets |
| 75 | Intermittent v. continuous energy restriction: Differential effects on postprandial glucose and lipid metabolism following matched weight loss in overweight/obese participants | The 5:2 diet and other diets |
| 76 | A nonrandomized controlled clinical pilot trial on 8 wk of intermittent fasting (24 h/wk) | The 5:2 diet and other diets |
| 77 | Comparison of high-protein, intermittent fasting low-calorie diet and heart healthy diet for vascular health of the obese | The 5:2 diet and other diets |
| 78 | Intermittent fasting combined with calorie restriction is effective for weight loss and cardio-protection in obese women | Different topic of interesting |
| 79 | Improvement in coronary heart disease risk factors during an intermittent fasting/calorie restriction regimen: Relationship to adipokine modulations | Different topic of interesting |
| 80 | Intermittent fasting does not affect whole-body glucose, lipid, or protein metabolism | Different topic of interesting |
| 81 | A controlled trial of reduced meal frequency without caloric restriction in healthy, normal-weight, middle-aged adults | Different topic of interesting |
| 82 | Time-Restricted Eating Without Calorie Counting for Weight Loss in a Racially Diverse Population | Articles can not get full text |
| 83 | Can intermittent, time-restricted circadian fasting modulate cutaneous severity of dermatological disorders? Insights from a multicenter, observational, prospective study | Articles can not get full text |
| **C. Included studies** | | |
| 1 | Effects of Time-Restricted Eating on Nonalcoholic Fatty Liver Disease: The TREATY-FLD Randomized Clinical Trial | Fulfill selection criteria |
| 2 | Implementation of weekday time-restricted eating to improve metabolic health in breast cancer survivors with overweight/obesity | Fulfill selection criteria |
| 3 | Effectiveness of Early Time-Restricted Eating for Weight Loss, Fat Loss, and Cardiometabolic Health in Adults With Obesity | Fulfill selection criteria |
| 4 | The Effects of Time-Restricted Eating versus Standard Dietary Advice on Weight, Metabolic Health and the Consumption of Processed Food: A Pragmatic Randomised Controlled Trial in Community-Based Adults | Fulfill selection criteria |
| 5 | Feasibility and acceptability of time-restricted eating in a group of adults with multiple sclerosis | Fulfill selection criteria |
| 6 | Time-restricted feeding’s effect on overweight and obese patients with chronic kidney disease stages 3-4: A prospective non-randomized control pilot study | Fulfill selection criteria |
| 7 | Early Time-Restricted Eating Reduces Weight and Improves Glycemic Response in Young Adults: A Pre-Post Single-Arm Intervention Study | Fulfill selection criteria |
| 8 | Effects of time-restricted feeding (16/8) combined with a low-sugar diet on the management of non-alcoholic fatty liver disease: A randomized controlled trial | Fulfill selection criteria |
| 9 | Three weeks of time-restricted eating improves glucose homeostasis in adults with type 2 diabetes but does not improve insulin sensitivity: a randomised crossover trial | Fulfill selection criteria |
| 10 | The feasibility and preliminary efficacy of early time-restricted eating on diet quality in college students: A randomized study | Fulfill selection criteria |
| 11 | The Fasting and Shifted Timing (FAST) of Eating Study: A pilot feasibility randomized crossover intervention assessing the acceptability of three different fasting diet approaches | Fulfill selection criteria |
| 12 | The Effects of Time‐Restricted Eating on Metabolism and Gut Microbiota: A Real‐Life Study | Fulfill selection criteria |
| 13 | Impact of Intermittent Fasting Combined With High-Intensity Interval Training on Body Composition, Metabolic Biomarkers, and Physical Fitness in Women With Obesity | Fulfill selection criteria |
| 14 | Early time-restricted eating compared with daily caloric restriction: A randomized trial in adults with obesity | Fulfill selection criteria |
| 15 | Calorie Restriction with or without Time-Restricted Eating in Weight Loss | Fulfill selection criteria |
| 16 | Time-restricted eating improves glycemic control and dampens energy-consuming pathways in human adipose tissue | Fulfill selection criteria |
| 17 | The effect of 4-h versus 6-h time restricted feeding on sleep quality, duration, insomnia severity and obstructive sleep apnea in adults with obesity | Fulfill selection criteria |
| 18 | Effect of time-restricted feeding on body composition and cardio-metabolic risk in middle-aged women in Taiwan | Fulfill selection criteria |
| 19 | Twelve Months of Time-restricted Eating and Resistance Training Improves Inflammatory Markers and Cardiometabolic Risk Factors | Fulfill selection criteria |
| 20 | Time-restricted feeding improves blood glucose and insulin sensitivity in overweight patients with type 2 diabetes: a randomised controlled trial | Fulfill selection criteria |
| 21 | Sequential diets and weight loss: Including a low-carbohydrate high-fat diet with and without time-restricted feeding | Fulfill selection criteria |
| 22 | The effects of a macronutrient-based diet and time-restricted feeding (16:8) on body composition in physically active individuals—a 14-week randomised controlled trial | Fulfill selection criteria |
| 23 | The effect of four weeks dietary intervention with 8-hour time-restricted eating on body composition and cardiometabolic risk factors in young adults | Fulfill selection criteria |
| 24 | A smartphone intervention to promote time restricted eating reduces body weight and blood pressure in adults with overweight and obesity: A pilot study | Fulfill selection criteria |
| 25 | Effect of time restricted eating on body weight and fasting glucose in participants with obesity: results of a randomized, controlled, virtual clinical trial | Fulfill selection criteria |
| 26 | Time-restricted eating for 12 weeks does not adversely alter bone turnover in overweight adults | Fulfill selection criteria |
| 27 | Impact of Intermittent Fasting on Lipid Profile–A Quasi-Randomized Clinical Trial | Fulfill selection criteria |
| 28 | Continuous energy restriction (CER) plus 16/8 time-restricted feeding improve body composition and metabolic parameters in overweight and obese, but no more than CER alone | Fulfill selection criteria |
| 29 | Applicability of time-restricted eating for the prevention of lifestyle-dependent diseases in a working population: Results of a pilot study in a pre-post design [Anwendbarkeit von zeitlich begrenzter nahrungsaufnahme zur prävention lebensstilabhängiger krankheiten in einer berufstätigen population: Ergebnisse einer pilotstudie im prä-post-design] | Fulfill selection criteria |
| 30 | Time-restricted eating effects on performance, immune function, and body composition in elite cyclists: a randomized controlled trial | Fulfill selection criteria |
| 31 | Effects of Time-Restricted Eating on Weight Loss and Other Metabolic Parameters in Women and Men with Overweight and Obesity: The TREAT Randomized Clinical Trial | Fulfill selection criteria |
| 32 | Does the energy restriction intermittent fasting diet alleviate metabolic syndrome biomarkers? A randomized controlled trial | Fulfill selection criteria |
| 33 | Two weeks of early time-restricted feeding (eTRF) improves skeletal muscle insulin and anabolic sensitivity in healthy men | Fulfill selection criteria |
| 34 | Time-Restricted Eating Effects on Body Composition and Metabolic Measures in Humans who are Overweight: A Feasibility Study | Fulfill selection criteria |
| 35 | The effects of time restricted feeding on overweight, older adults: A pilot study | Fulfill selection criteria |
| 36 | Effects of 8-hour time restricted feeding on body weight and metabolic disease risk factors in obese adults: A pilot study | Fulfill selection criteria |
