# Supplementary material for: Partially unraveling mechanistic underpinning and weight loss effects of time-restricted eating across diverse adult populations: A systematic review and meta-analyses of prospective studies
Source: PLoS One. 2025 Jan 15;20(1):e0314685. doi: 10.1371/journal.pone.0314685 (PMC11734929; doi:10.1371/journal.pone.0314685)
Supplement: S5 Table — (DOCX) [file pone.0314685.s006.docx]

**Supplementary S15. Assessment of bias risk in randomized controlled trials included in the meta-analysis using the Cochrane Risk of Bias 2 tool ^1^**

| **Study (Year)** | **1. Bias due to the randomization process** | **2. Deviation from intended intervention** | **3. Missing outcome data** | **4. Measurement of outcomes** | **5. Selection of the reported result** | **Overall risk of bias** |
| --- | --- | --- | --- | --- | --- | --- |
| Xueyun Wei (2023) | Low | Low | Low | Low | Low | Low |
| Amy A. Kirkham (2023) | High | Low | Low | Low | Low | Some concerns |
| Humaira Jamshed (2022) | Low | Low | Some concerns | Some concerns | Low | Some concerns |
| Nicholas Edward Phillips (2021) | Low | Low | Low | Low | Low | Low |
| Brooks C. Wingo (2022) | High | Low | Low | Low | Low | High |
| Bei-ni Lao (2023) | Low | Low | Low | Low | Low | Low |
| JinA Kim (2023) | Some concerns | Low | Low | Low | Low | Some concerns |
| Hamed Kord-Varkaneh (2023) | Low | Low | Low | Low | Low | Low |
| Charlotte Andriessen (2022) | Low | Low | Low | Low | Low | Low |
| ﻿Selicia T. Mayra (2022) | Low | Low | Low | Low | Low | Low |
| Gabrielle M. Turner-McGrievy (2022) | Low | Low | Low | Low | Low | Low |
| Ilario Ferrocino (2022) | High | Some concerns | Low | Low | Low | High |
| Gabriela Batitucci (2022a) | Low | Low | Low | Low | Low | Low |
| Elizabeth A. Thomas (2022) | Low | Low | Some concerns | Low | Low | Some concerns |
| Deying Liu (2022) | Low | Low | Low | Low | Low | Low |
| Lijun Zhao (2022) | High | Low | Some concerns | Low | Low | High |
| Sofia Cienfuegos (2022) | Low | Some concerns | Some concerns | Low | Low | Some concerns |
| Yan-Ju Lin (2022) | Some concerns | Low | Low | Low | Low | Low |
| Tatiana Moro (2021) | Low | Low | Low | Low | Low | Low |
| Tingting Che (2021) | Low | Low | Low | Low | Low | Low |
| Rebecca A.G. Christensen (2021) | High | High | Low | Low | Low | High |
| Eduard Isenmann (2021) | Low | Low | Low | Low | Low | Low |
| Su-Jeong Park (2021) | Low | Low | Low | Low | Low | Low |
| Malini Prasad (2021) | Low | High | Some concerns | Low | Low | High |
| Pamela M. Peeke (2021) | Some concerns | Low | Low | High | Low | High |
| Andrea J. Lobene (2021) | Some concerns | Low | Some concerns | Low | Low | Some concerns |
| Naseer Ahmed (2020) | High | High | Low | Low | Low | High |
| Daiani Evangelista Ribeiro (2021) | High | Low | Low | Low | Low | Low |
| Dorothea Kesztyüs (2021) | Low | Some concerns | Low | Low | Low | Low |
| Tatiana Moro (2020) | Low | Low | Low | Low | Low | Low |
| ﻿Dylan A. Lowe (2021) | Low | Low | Low | Low | Low | Low |
| Yasemin Ergul Kunduraci (2020) | Low | Some concerns | Some concerns | Low | Low | Low |
| Robert Jones (2020) | High | Low | Low | Low | Low | High |
| Lisa S. Chow (2020) | Low | Low | Low | Low | Low | Low |
| Stephen D. Anton (2019) | Low | Some concerns | Low | Low | Low | Low |
| Kelsey Gabel (2018) | Low | Low | Low | Low | Low | Low |

^1^ Sterne JAC, Savović J, Page MJ, Elbers RG, Blencowe NS, Boutron I, Cates CJ, Cheng H-Y, Corbett MS, Eldridge SM, Hernán MA, Hopewell S, Hróbjartsson A, Junqueira DR, Jüni P, Kirkham JJ, Lasserson T, Li T, McAleenan A, Reeves BC, Shepperd S, Shrier I, Stewart LA, Tilling K, White IR, Whiting PF, Higgins JPT. RoB 2: a revised tool for assessing risk of bias in randomised trials. *BMJ* 2019; **366**: l4898
