# Supplementary material for: Partially unraveling mechanistic underpinning and weight loss effects of time-restricted eating across diverse adult populations: A systematic review and meta-analyses of prospective studies
Source: PLoS One. 2025 Jan 15;20(1):e0314685. doi: 10.1371/journal.pone.0314685 (PMC11734929; doi:10.1371/journal.pone.0314685)
Supplement: S2 Fig — (DOCX) [file pone.0314685.s008.docx]

**Supplementary S4.** Funnel plot of risk of publication bias for the effect of time-restricted eating on weight loss in general adults


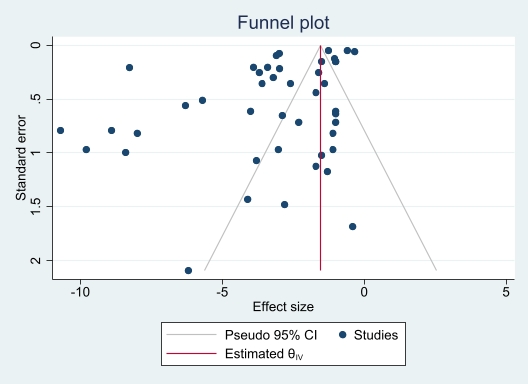


Abbreviations: CIs, confidence intervals
