# Supplementary material for: Partially unraveling mechanistic underpinning and weight loss effects of time-restricted eating across diverse adult populations: A systematic review and meta-analyses of prospective studies
Source: PLoS One. 2025 Jan 15;20(1):e0314685. doi: 10.1371/journal.pone.0314685 (PMC11734929; doi:10.1371/journal.pone.0314685)
Supplement: S8 Fig — (DOCX) [file pone.0314685.s014.docx]

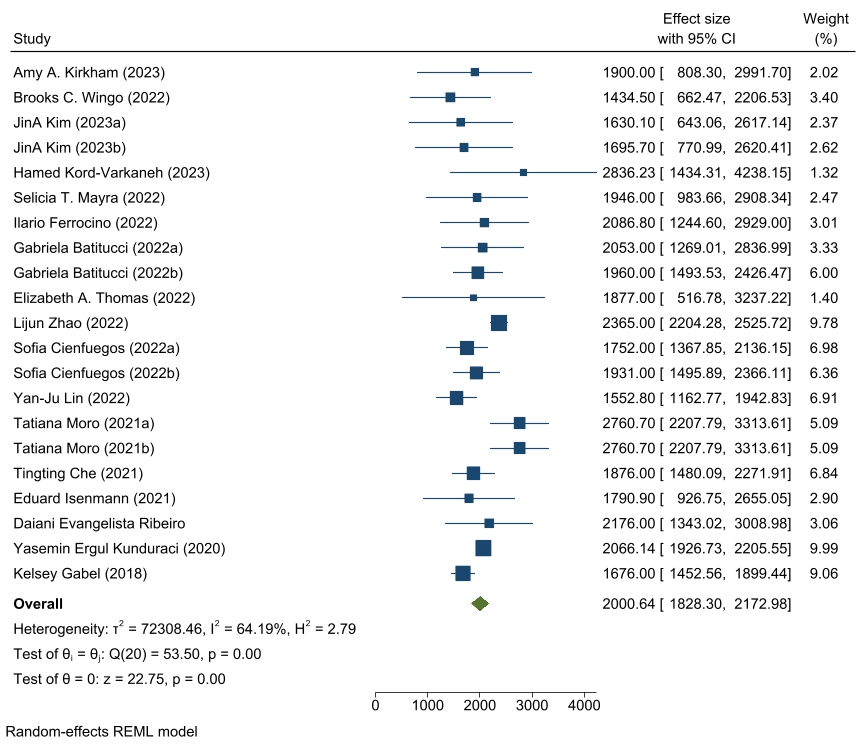
**Supplementary S10.** Forest Plot Illustrating the Pooled Mean Caloric Intake at Baseline and the Conclusion of the TRE Intervention Period.

**Supplementary S10.1.** The analysis was performed using a random-effects model. Squares depict the effect size for each study, summarized as the mean difference (MD) with corresponding 95% CIs. Individual study-specific MDs and 95% CIs are represented by blue squares and horizontal lines, respectively. Green diamond represents overall effect size and 95% CIs. Abbreviations: CIs, confidence intervals; REML: The restricted maximum likelihood method.


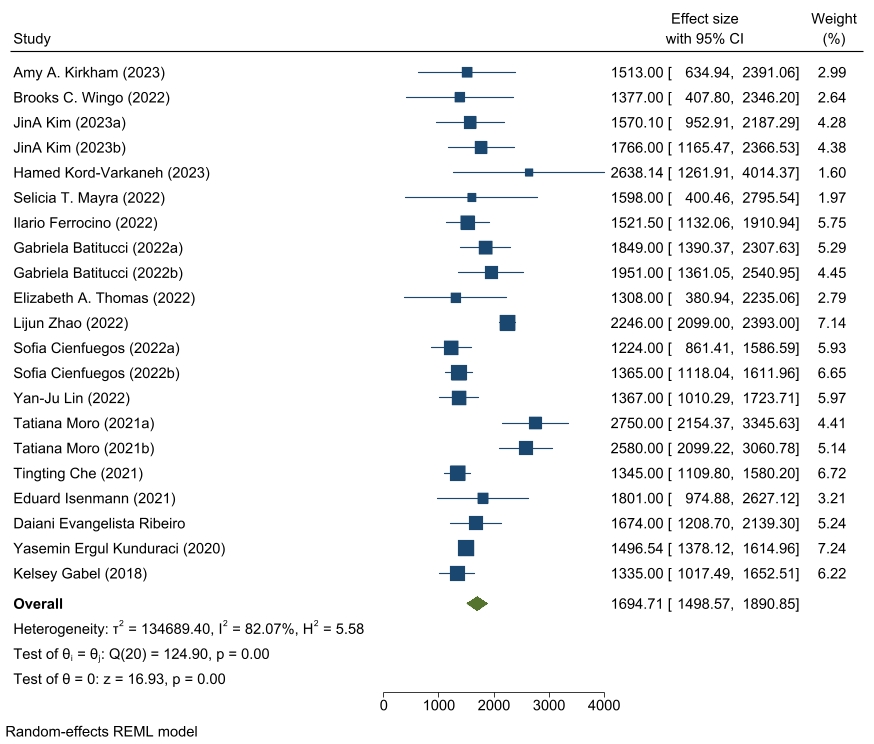
 **Supplementary S10.2.** The analysis was performed using a random-effects model. Squares depict the effect size for each study, summarized as the mean difference (MD) with corresponding 95% CIs. Individual study-specific MDs and 95% CIs are represented by blue squares and horizontal lines, respectively. Green diamond represents overall effect size and 95% CIs. Abbreviations: CIs, confidence intervals; REML: The restricted maximum likelihood method.
