# Supplementary material for: Partially unraveling mechanistic underpinning and weight loss effects of time-restricted eating across diverse adult populations: A systematic review and meta-analyses of prospective studies
Source: PLoS One. 2025 Jan 15;20(1):e0314685. doi: 10.1371/journal.pone.0314685 (PMC11734929; doi:10.1371/journal.pone.0314685)
Supplement: S9 Fig — (DOCX) [file pone.0314685.s015.docx]

**Supplementary S11.** Univariate Linear Regression Analysis of Caloric Intake (in 13 Studies Characterized by an Ad Libitum Approach) and Mean Age in Relation to Mean Differences in Weight Loss.


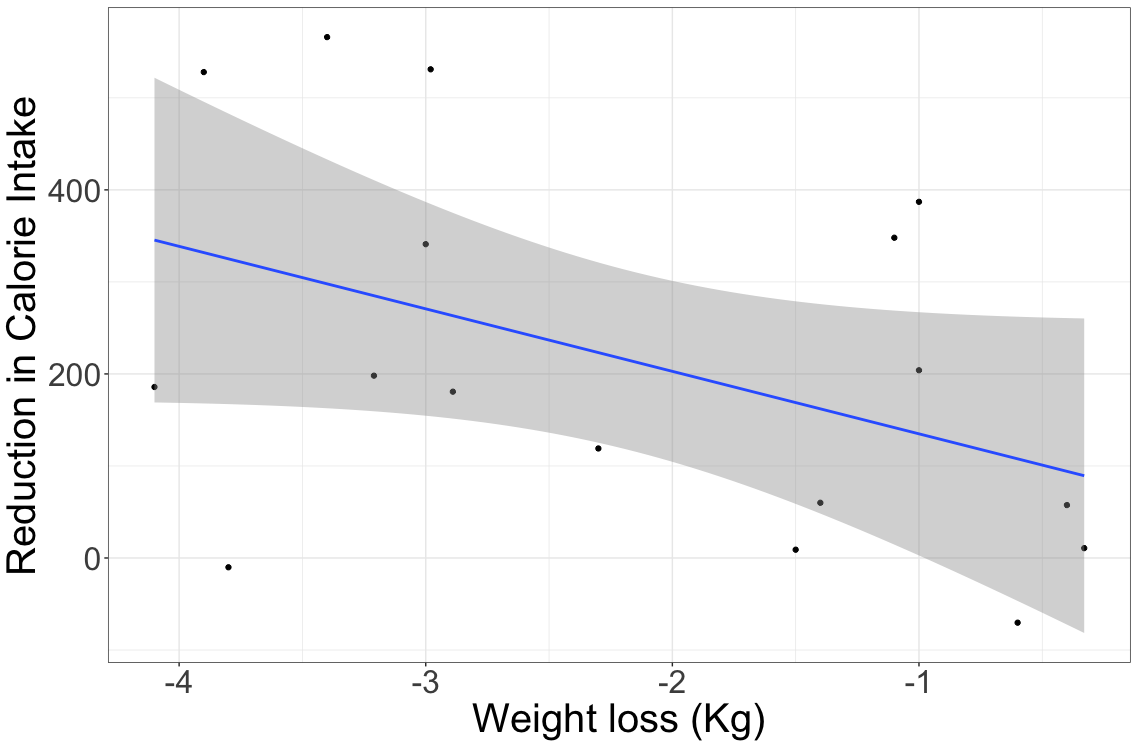


**Supplementary S11.1. Univariate Linear Regression Analysis of Caloric Intake (in 13 Studies Characterized by an Ad Libitum Approach) in Relation to Mean Differences in Weight Loss.** Abbreviations: kg, kilogram.


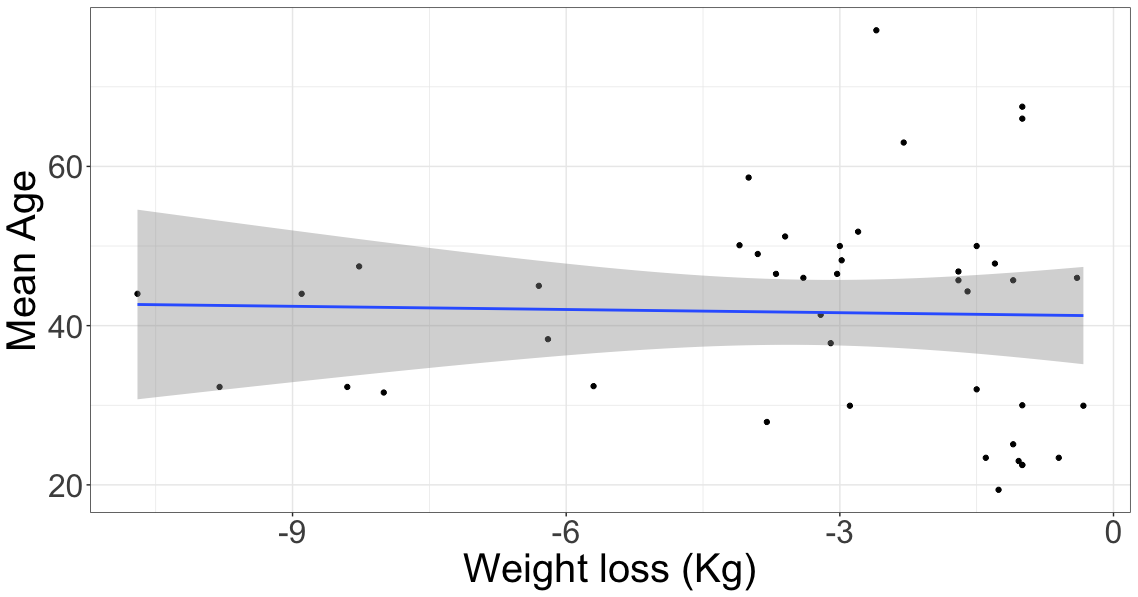


**Supplementary S11.2. Univariate Linear Regression Analysis of Mean Age in Relation to Mean Differences in Weight Loss.** Abbreviations: kg, kilogram.
